# Management of plantar heel pain: a best practice guide informed by a systematic review, expert clinical reasoning and patient values

**DOI:** 10.1136/bjsports-2019-101970

**Published:** 2021-03-30

**Authors:** Dylan Morrissey, Matthew Cotchett, Ahmed Said J'Bari, Trevor Prior, Ian B Griffiths, Michael Skovdal Rathleff, Halime Gulle, Bill Vicenzino, Christian J Barton

**Affiliations:** 1 Sports and Exercise Medicine, William Harvey Research Institute, Queen Mary University of London, London, UK; 2 Physiotherapy Department, Barts Health NHS Trust, London, UK; 3 Department of Physiotherapy, Podiatry, Prosthetics and Orthotics, La Trobe University, Melbourne, Victoria, Australia; 4 Department of Health Science and Technology, Faculty of Medicine, Aalborg University, Aalborg, Denmark; 5 University of Queensland, School of Health and Rehabilitation Sciences: Physiotherapy, St Lucia, Queensland, Australia; 6 Department of Surgery, St Vincent’s Hospital, University of Melbourne, Melbourne, Victoria, Australia

**Keywords:** qualitative, rehabilitation, effectiveness, foot

## Abstract

**Objective:**

To develop a best practice guide for managing people with plantar heel pain (PHP).

**Methods:**

Mixed-methods design including systematic review, expert interviews and patient survey.

**Data sources:**

Medline, Embase, CINAHL, SPORTDiscus, Cochrane Central Register of Controlled Trials, trial registries, reference lists and citation tracking. Semi-structured interviews with world experts and a patient survey.

**Eligibility criteria:**

Randomised controlled trials (RCTs) evaluating any intervention for people with PHP in any language were included subject to strict quality criteria. Trials with a sample size greater than n=38 were considered for proof of efficacy. International experts were interviewed using a semi-structured approach and people with PHP were surveyed online.

**Results:**

Fifty-one eligible trials enrolled 4351 participants, with 9 RCTs suitable to determine proof of efficacy for 10 interventions. Forty people with PHP completed the online survey and 14 experts were interviewed resulting in 7 themes and 38 subthemes. There was good agreement between the systematic review findings and interview data about taping (SMD: 0.47, 95% CI 0.05 to 0.88) and plantar fascia stretching (SMD: 1.21, 95% CI 0.78 to 1.63) for first step pain in the short term. Clinical reasoning advocated combining these interventions with education and footwear advice as the core self-management approach. There was good expert agreement with systematic review findings recommending stepped care management with focused shockwave for first step pain in the short-term (OR: 1.89, 95% CI 1.18 to 3.04), medium-term (SMD 1.31, 95% CI 0.61 to 2.01) and long-term (SMD 1.67, 95% CI 0.88 to 2.45) and radial shockwave for first step pain in the short term (OR: 1.66, 95% CI 1.00 to 2.76) and long term (OR: 1.78, 95% CI 1.07 to 2.96). We found good agreement to ‘step care’ using custom foot orthoses for general pain in the short term (SMD: 0.41, 95% CI 0.07 to 0.74) and medium term (SMD: 0.55, 95% CI 0.09 to 1.02).

**Conclusion:**

Best practice from a mixed-methods study synthesising systematic review with expert opinion and patient feedback suggests core treatment for people with PHP should include taping, stretching and individualised education. Patients who do not optimally improve may be offered shockwave therapy, followed by custom orthoses.

## Background

Plantar heel pain (PHP), which affects 4%–7.0% of the community,^1–4^ is associated with impaired health-related quality of life including social isolation, a poor perception of health status and reduced functional capabilities.^5^ PHP predominantly affects sedentary middle-aged and older adults, and is estimated to account for 8.0% of all injuries related to running.^6^ The condition is characterised by first step pain and pain during weight-bearing tasks, particularly after periods of rest.^7^

How should clinicians treat pain and improve function in people with PHP? The published literature is dominated by systematic reviews, guidelines and meta-analyses^7–9^ that include low-quality trials with small sample sizes, which may inflate effect sizes and lead to incorrect interpretation.^10^ Two previously published Clinical Practice Guides for PHP, based on variable quality evidence, do not recommend one treatment over another.^7 8^ A recent network meta-analysis included low-quality studies and limited the analysis to studies of extracorporeal shockwave therapy (ESWT), exercise, corticosteroid injections, non-steroidal anti-inflammatory drug (NSAID) injections, oral NSAIDs and orthoses. This paper failed to capture the effect of all possible interventions, for example, not considering taping, dry needling and a range of other interventions.^9^ We aim to guide management of PHP based on high-quality evidence for any intervention. We augmented published efficacy study findings with expert reasoning and patient experience.

In short, we synthesised findings from high-quality level II randomised controlled trials (RCTs) with clinical reasoning from clinician-researchers and the patient voice, to develop a best practice guide (BPG) to the clinical management of PHP.

## Methods

The systematic review element was registered on PROSPERO (CRD42018102227), without any deviation from the published protocol. Funding was received from the Private Physiotherapy Education Fund, in the UK, who took no part in the design, conduct or reporting of the research. The review adhered to the guidelines for good reporting of a mixed-methods study (online supplemental file 1) and Preferred Reporting Items for Systematic Reviews and Meta-Analyses statement for systematic reviews.

10.1136/bjsports-2019-101970.supp1 Supplementary data

### Search methods

The final search was October 2019 of Ovid MEDLINE (1950 to date), Ovid EMBASE (from 1988 to date), CINAHL (1982 to date). SPORTDiscus, Cochrane Central Register of Controlled Trials and Web of Science were searched from inception (see online supplemental file 1 for details). In addition, we also searched ClinicalTrials.gov (http://clinicaltrials.gov/), the WHO International Clinical Trials Registry Platform (http://apps.who.int/trialsearch/) and ControlledTrials.com (http://www.isrctn.com/) for ongoing or completed trials that met the eligibility criteria. Finally, references of included studies and citing articles were used to identify trials not located through the database search. No language restrictions were applied.

### Criteria for selecting studies

#### Type of studies

Published RCTs evaluating the efficacy or effectiveness of any intervention for PHP were considered for inclusion. No language restrictions were imposed. All other designs including quasi-experimental studies, letters to the editor, opinion pieces, editorials and conference abstracts were considered ineligible.

#### Characteristics of participants

An RCT was included if the participants were clinically diagnosed with PHP with explicit reference to pain on the underside of the heel that was most noticeable on weight-bearing after periods of rest but also worse following prolonged weight bearing. All participants were over the age of 16 years and had experienced symptoms of any duration. RCTs were excluded if participants’ PHP was related to fractures, tumours or infections or confounded by other conditions that might be rheumatological, neural, vascular or dermatological in origin.

#### Types of interventions

Any RCT that investigated the effectiveness or efficacy of an intervention compared with another intervention, placebo, sham or wait and see was included, provided there was a follow-up of at least 2 weeks.

#### Outcome measures

Studies that reported at least one of the three outcome measures—patient-reported pain, first step pain or foot-related function—were included. Where multiple methods of quantifying the same outcome were reported, the author’s main prespecified outcome metric was accepted, or that of the metric with the lower level of effect. This was done to avoid making recommendations based on type 1 error with the rationale that an intervention which results in a meaningful effect size for a given outcome should be consistent irrespective of the measure used.

Short term was defined as 1 week to 3 months, medium term as >3 and up to 6 months and long term as >6 months. Where multiple follow-ups within a time window were reported, the outcome at the latest time point within short and medium term, and closest to 1 year for long term was selected.

### Data collection and analysis

#### Selection of studies and assessment of quality

The results of the database searches were exported to EndNote X8 (Thomson Reuters, New York, USA) and duplicates removed. Titles were initially screened against the eligibility criteria. Relevant titles and abstracts were independently assessed by pairs of reviewers with disagreements resolved by MC and DM. The PEDro scale^11^ is an 11-point scale that was used to evaluate internal validity and statistical interpretability with higher scores indicating greater quality. The PEDro database was screened to determine if a study had previously been reviewed and a quality score assigned. If a study had been graded, one reviewer independently rated the study, else two reviewers performed the rating with any disagreements from either method being resolved by a third reviewer. A study that scored ≥8/10 on the PEDro scale was considered to be of high quality and retained,^12^ as a strong correlation exists between the PEDro scale and the Cochrane risk of bias (RoB) tool, indicating evidence for strong convergent validity.^13^

For all RCTs that scored ≥8/10, the RoB was evaluated at study level using six specific items of the PEDro scale,^14^ chosen based on a review of the Cochrane Collaboration tool for assessing RoB^15^ and those factors considered to influence internal validity and the size of the effect in RCTs.^16^ The items were random sequence generation (item 2), allocation concealment (item 3), baseline comparability (item 4), blinding of outcome assessors (item 7), adequate follow-up (item 8) and intention-to-treat analysis (item 9). Studies that scored <5 out of 6 were considered to have a high RoB and excluded from the review. These factors were used as inclusion criteria to enter the review, rather than being used to weight the review findings. Furthermore, in response to recent recommendations in *British Journal of Sports Medicine*,^17 18^ a subsequent RoB check was made of the 10 studies informing the determination of primary or secondary proof of efficacy using the RoB-2 tool^19^ to determine whether the methods we had employed had been robust in selecting out studies with an acceptable RoB.

#### Data extraction and management

A data extraction form was used to record outcome data related to pain, first step pain and function, by pairs of authors and checked by MC. The mean and SD, median and IQR or difference in outcomes from baseline was extracted for continuous data. In addition, dichotomous data were extracted such as successful outcome. Data were extracted for each outcome at all time points and entered into RevMan (V.5.3; Copenhagen: The Nordic Centre, The Cochrane Collaboration, 2014).

#### Dealing with missing data

For studies that did not report the number of participants at specific time points, or where participants were lost to follow-up, the meta-analyses were based on the published intention-to-treat data. Where data were missing, or not in a usable format, we attempted to contact the corresponding author of the study. If a request for data was not provided, we attempted to calculate SDs from SEs, CIs or p values, according to the Cochrane Handbook for Systematic Reviews of Interventions.^15^

For studies that reported median and range data, we estimated the mean and variance from the median, range and the size of a sample. Where the sample size was >25, the median value was used to estimate the mean. To estimate the variance for samples 15<n≤70 and >70, we used the formula range/4 and range/6, respectively to calculate the SD.^20^

#### Evaluating efficacy and the strength of the evidence

An intervention was considered to demonstrate efficacy if adequately powered included trials demonstrated (i) primary proof of superiority compared with sham or placebo or (ii) secondary proof of superiority compared with another treatment of proven efficacy or (iii) secondary proof of equivalence results to another treatment of proven efficacy, with the previously proven treatment yielding similar results to the initial RCT proving its efficacy. If an intervention was not found to be superior when compared with sham or placebo, it was deemed to be ineffective and if it was compared with another unproven intervention, and no difference in effect noted, then it was regarded as not being adequately tested. Where there were conflicting results between different studies for any outcomes at any time-point, efficacy was resolved by meta-analysis.

Adequate sample size was calculated using G*Power (Universitat Dusseldorf, http://www.gpower.hhu.de/en.html) at a power of 80%, a minimum important difference of 19 on a visual analogue scale (VAS) score for first step pain,^21^ an SD of 28^22^ and 5% alpha level defining a minimum sample size of 38 per group. This outcome measure was chosen as it is arguably the pathognomonic feature of PHP. In addition, 38 per group also represented the lowest calculated sample size for all our prespecified outcome measures (ie, overall pain and function) that we evaluated. If studies did not have an adequate sample size to be considered for primary or secondary proof of efficacy, they were included in meta-analysis where their results could be pooled with an adequately powered study.

To evaluate the overall strength of the evidence for each intervention, we used the levels of evidence system designed by van Tulder *et al*,^23^ which was adapted to reflect study inclusion being limited to high-quality studies and applied for both positive and negative findings. Evidence was rated as:

- *Strong evidence/Positive effect*: meta-analysis revealed multiple high-quality trials demonstrated efficacy/a positive effect in favour of the intervention.
- *Moderate evidence/Positive effect*: analysis revealed one high-quality trial demonstrated efficacy/a positive effect in favour of the intervention.
- *Limited evidence/Positive effect*: analysis revealed one high-quality trial, which did not meet the required sample size, demonstrated efficacy/a positive effect in favour of the intervention.
- *Strong evidence/Neutral effect*: meta-analysis revealed multiple high-quality trials demonstrated no efficacy/evidence of no effect.
- *Moderate evidence/Neutral effect*: analysis revealed one high-quality trial demonstrated no efficacy/evidence of no effect.
- *Limited evidence/Neutral effect*: analysis revealed one high-quality trial, which did not meet the required sample size, demonstrated no efficacy/evidence of no effect.
- *Conflicting*: where there were conflicting between-study intervention results for any outcomes at any time-point, efficacy was resolved by meta-analysis.

#### Data synthesis

For studies reporting continuous data, we calculated standardised mean differences (SMD), irrespective of whether the outcomes were similar or different but ensuring only outcomes of a similar construct were combined. Effect sizes were set as 0.20–0.49 being small, 0.50–0.79 as medium and 0.80 or above as large.^24^ Final scores at each follow-up period were evaluated in preference to mean or median change from baseline values, unless only change scores were available^15^ as the use of follow-up scores is more conservative and are less likely to find significant results.^25^ For studies reporting dichotomous data, to represent treatment success, ORs were calculated. For studies that included three or more active treatment arms, and reported continuous outcomes, the active arms were combined and compared with the control group, to avoid a unit of analysis error,^26^ using accepted methods according to the Cochrane Handbook for Systematic Reviews of Interventions.^15^

Comparable studies, reporting continuous data, were pooled using an inverse variance weighting method within a random-effects model.^27^ For dichotomous outcome variables, the Mantel-Haenszel method was used to estimate an association between a treatment and outcome. The χ^2^ test and I^2^ statistic were used to evaluate statistical heterogeneity. Finally, to evaluate the overall outcome of an intervention of proven efficacy, individual studies that evaluated a specific intervention were combined to determine the size of within-group changes.

### Semi-structured interviews with international experts

#### Participants

International experts, defined as having a minimum of 5 years of experience in a given setting and specialty in which they regularly encountered significant numbers of people with PHP and who were actively involved in PHP research, were purposively recruited.^28^ Experts were identified by the authors through recommendations of researchers that publish in the field of PHP and snowball sampling. All experts were invited to participate via email.

In total, 14 expert clinicians (6 from Australia, 3 from Denmark, 2 from the UK, 2 from the USA and 1 from Canada) were recruited and interviewed, of which 7 were physiotherapists, 6 were podiatrists (including 2 podiatric surgeons) and 1 was a rheumatologist. Experts had a mean monthly exposure to 9 patients with PHP per month and had published an average of 51 publications (online supplemental file 1).

### Interview process

One of two interviewers (AH and DM) conducted each interview online or face-to-face lasting between 30 and 90 min. An interview topic guide, piloted before use, was constructed based on a preliminary literature search, discussions within the research team and emergent concepts from pilot interviews conducted (online supplemental file 1). Questions explored interviewees’ background, clinical reasoning when managing PHP, perceptions of the evidence and any gaps in the published literature on treatment of PHP. An online graphic containing descriptors of possible interventions was presented at mid-interview as a stimulus to discussion.^29^ Interviews were transcribed verbatim. Data were collected until thematic saturation, defined as the stage at which no new patterns or themes emerged.^30^

### Analysis of interview data

Interviews were recorded, transcribed and analysed by DM after or concomitantly with data collection using the Framework approach.^30^ This entailed construction of an initial thematic framework from the topic guide following a process of data familiarisation. The themes and subthemes continuously evolved throughout the process of analysis by identifying emerging topics and mapping the ideas and beliefs of the recruited experts. The framework was applied to the data by coding each section of the text to subthemes and grouping them into themes, subthemes and supporting quotations as an initial descriptive analysis. This analysis was extended to identify typologies and tensions in the data to combine with systematic review data and yield the BPG.

### Patient and public involvement—survey of patients with PHP

**Figure 1:**
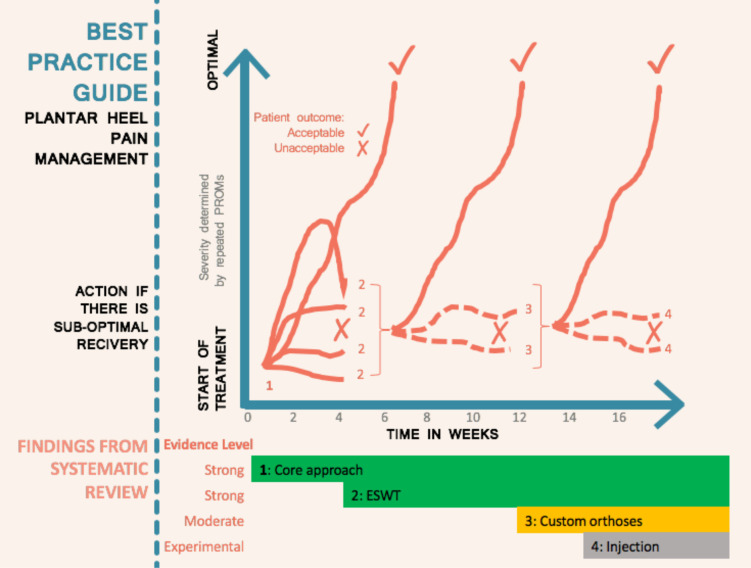
Management approach for plantar heel pain when a person progressively fails to recover with addition of extracorporeal shockwave therapy (ESWT) at 4 weeks if the core approach is not working and then addition of orthoses at 12 weeks if there is still suboptimal improvement. PROM, patient-reported outcome measure.

Interim results of the evidence synthesis (figure 1) were presented to patients using an online survey (www.surveymonkey.co.uk) (online supplemental file 1). Open questions were asked, which explored a persons’ experience of living with PHP, understanding of the nature of their PHP, expectations of clinicians, strengths of PHP management and areas for improvement. Results were analysed with the Framework approach.^30^

### Best practice guide formulation

**Figure 2:**
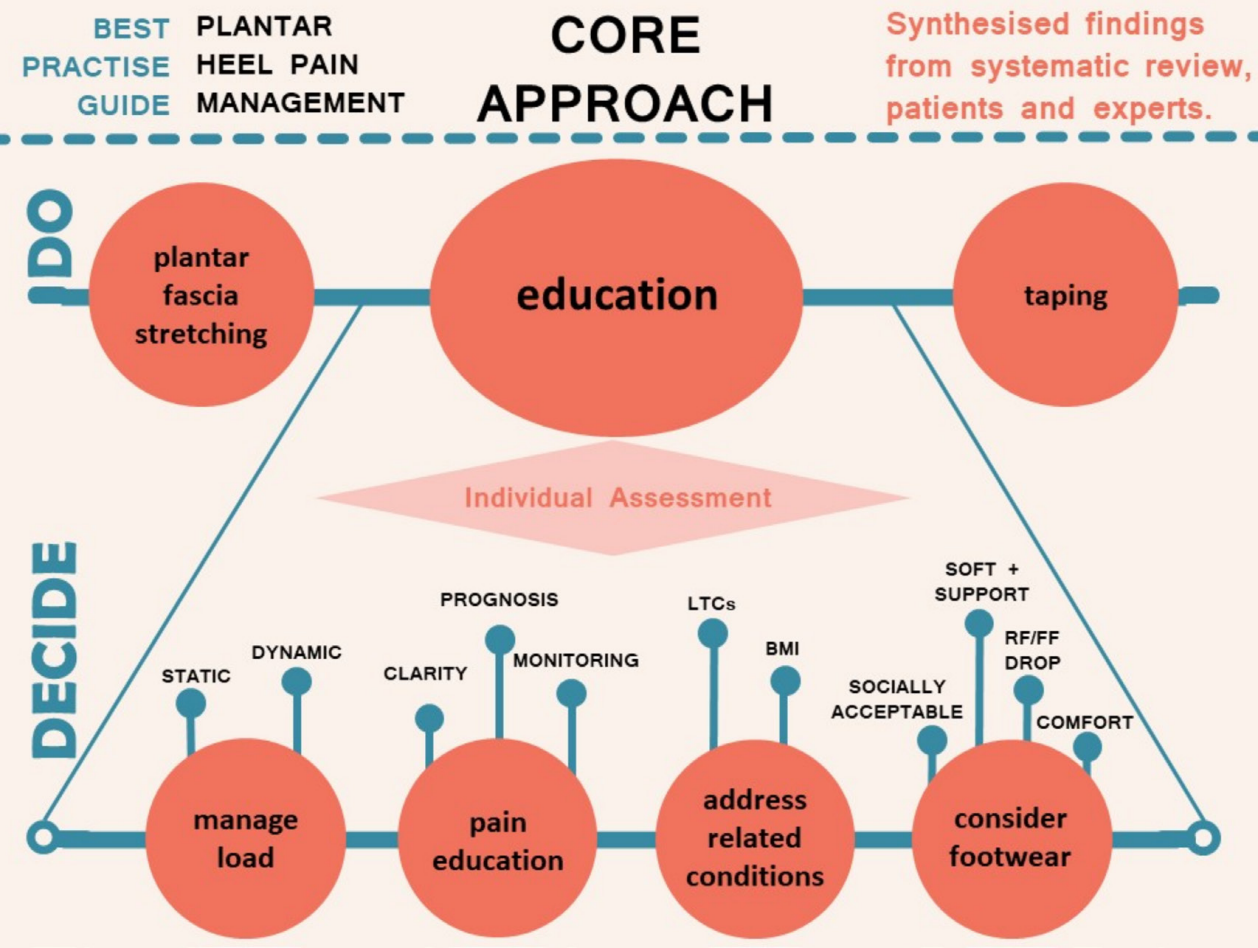
Core approach to the management of plantar heel pain based on the best available evidence, expert opinion and the patient voice. The top layer (‘DO’) of taping, stretching and education are required initial interventions with each patient. The individual assessment (‘DECIDE’) is of which specific educational aspects are needed. BMI, body mass index; FF, forefoot; LTC, long-term condition; RF, rearfoot.

The BPG was constructed by interpreting the systematic review findings through the lens of the clinical reasoning and evidence perspectives derived from the expert interviews and patient survey to generate a core approach (figure 2). In other words, *what to include* in the BPG came predominantly from the review, while *when and how* to combine and apply interventions came mainly from the interviews and survey. Specific interventions are therefore mainly review-determined, service delivery/clinical reasoning derived from the qualitative study, with confirmation of patient acceptance arising from the survey.

Furthermore, a quantitative check of the expected features of high-quality management was matched to the content of the core approach, which patients had not seen, using counts of agreement. The BPG was amended based on the patient survey results and presented as summary infographics and explanatory text in order to facilitate dissemination to both patients and professionals. This approach enabled consideration of multiple relevant perspectives, and included both qualitative and quantitative findings, as per recommended approaches that facilitate breadth and depth of understanding alongside corroboration from multiple sources.^31^ The qualitative–quantitative balance was predetermined so that inclusion of interventions was prioritised for systematic review findings, whereas ways of applying these interventions came from the expert interviews and patient survey.

## Results

### Search results

**Figure 3:**
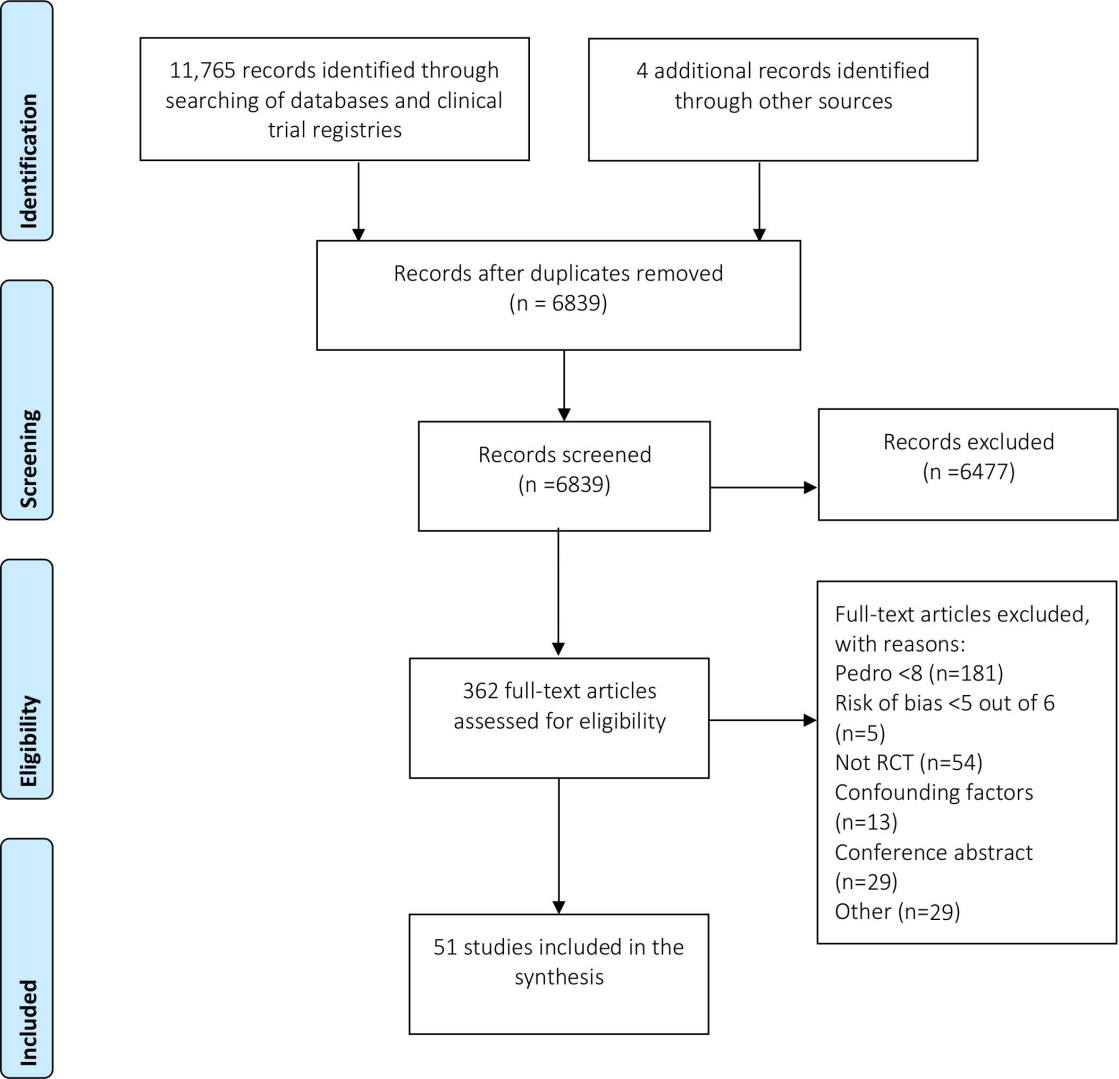
Flow diagram for study selection process. RCT, randomised controlled trial.

A total of 11 765 studies were identified through electronic databases and clinical trial registries (figure 3). After removal of duplicates, 6839 titles were screened. A total of 6477 studies were subsequently excluded leaving 362 studies to be assessed for full-text retrieval and PEDro analysis. Following the quality analysis, 51 studies met the eligibility criteria and were available for analysis. In total, the review included 4351 participants.

The characteristics of the included studies, including the treatment arms, outcome measures and participant characteristics are detailed in online supplemental file 1. Most studies had a small sample size (median=75, IQR=62). The mean duration of symptoms was 13 months (range 0.8–68.3 months) and 49% were female participants. Pain was evaluated in all studies either by a VAS or numerical pain rating scale. Other outcome measures included the Foot Function Index (9 studies), Roles and Maudsley score (7 studies), plantar fascia thickness (6 studies), Foot Health Status Questionnaire (10 studies), the Manchester-Oxford Foot Questionnaire (2 studies), Lower Extremity Functional Scale (3 studies), Foot Ankle Ability Measure (2 studies), Maryland Foot Score (1 study), Global Rating of Change (1 study), EQ-5D (study), 36-Item Short Form Survey (1 study) and the Foot and Ankle Outcome Score (1 study). Finally, the mean length of follow-up was 20 weeks (range 2–104 weeks). Ninety-six per cent of studies evaluated outcomes in the short term, but only 27% in the medium term and 13% in the long term.

Of the included studies, the type of interventions evaluated included ESWT (n=14), foot orthoses (n=8), night splints (n=1), footwear (n=1), flip-flop sandals (n=1), magnetised insoles (n=1), local injections (corticosteroid (n=11), botulinum toxin A injections (n=1), polydeoxyribonucleotide (n=2), hyaluronate injection (n=1), ozone (n=2), micronised dHACM (n=1), platelet-rich plasma (n=1)) manual therapy and exercise (n=2), low-level laser (n=2), radiation therapy (n=1), pulsed radiofrequency (n=2), stretching (n=1), trigger point dry needling (n=1), taping and iontophoresis (n=1), taping (n=1), electrolysis (n=1) and wheatgrass cream (n=1).

### Quality assessment

The quality analysis results for studies that met the eligibility criteria are included in online supplemental file 1. The PEDro scores ranged from 8/10 to 10/10. Four studies scored 10/10. ‘Blinding of all therapists who administered the therapy’ was the criterion least met by the included studies (n=7). In contrast, item 11 (‘the study provides both point measures and measures of variability for at least one outcome’) was the item most successfully completed (n=51).

### Risk of bias

RoB determined by the PEDro subset was low, as per the inclusion criteria. The subsequent check with the ROB-2 tool by three experienced reviewers (DM, MC, CJB) resulted in consensus that 48 of 50 domains in the 10 key trials had low RoB, with a maximum of 2 giving some concerns and none at high risk (online supplemental file 1). A justification and support for each judgement across all domains and studies is included in online supplemental file 1. Future trials with more transparent protocol adherence and blinding, where that is possible, would further improve the RoB scores in future studies.

### Evidence of efficacy

Table 1 provides a summary of the efficacy and strength of the evidence for interventions that were included for primary or secondary proof of efficacy. Online supplemental file 1 includes the short-term, medium-term and long-term results for each trial included in the analysis; a summary of those studies with evidence against efficacy and forest plots for the effectiveness of foot orthoses and ESWT.

**Table 1:** Efficacy and strength of evidence for interventions considered for primary and secondary proof of efficacy in the form of an ‘evidence and gap map’*

Intervention	Outcome measure		Short term†	Medium term†	Long term†
**Interventions with primary proof of efficacy**					
Custom orthoses	Pain	Between-group efficacy	Strong positive**34** 44–46 0.41 (0.07 to 0.74)	Limited positive^45^ 0.55 (0.09 to 1.02)	Moderate neutral**34** 0.04 (−0.37 to 0.45)
		Within-group outcome	1.24 (1.00 to 1.49),^34 44–46 62^‡	1.65 (1.12 to 2.18),^45^‡	
	First step pain	Between-group efficacy	Limited neutral^44 46^ −0.32 (−0.91 to 0.26)		
		Within-group outcome			
	Function	Between-group efficacy	Moderate neutral**34** 45 46 −0.21 (−0.48 to 0.06)	Limited neutral^45^ −0.39 (−0.85 to 0.07)	Moderate neutral**34** −0.12 (−0.53 to 0.29)
		Within-group outcome			
Prefabricated orthoses	Pain	Between-group efficacy	Moderate neutral**34** 46 −0.25 (−0.59 to 0.09)		Moderate neutral**34** −0.08 (−0.50 to 0.33)
		Within-group outcome			
	First step pain	Between-group efficacy			
		Within-group outcome			
	Function	Between-group efficacy	Moderate neutral**34** 46 −0.06 (−0.40 to 0.28)		Moderate neutral**34** −0.08 (−0.50 to 0.33)
		Within-group outcome			
Magnetised insoles	Pain	Between-group efficacy	Moderate neutral**36** 0.00 (−0.39 to 0.39)		
		Within-group outcome			
Radial ESWT	Pain	Between-group efficacy	Strong positive**32** 42 1.64 (−1.06 to 4.33)§	Limited positive^42^ 3.77 (2.82 to 4.72)	Strong positive**32** 42 0.78 (−0.15 to 1.72)§
		Within-group outcome	3.78 (−1.38 to 6.17)**32** 42 63 64 §	5.81 (3.57 to 8.05)^42 63^	6.41 (4.99 to 7.83)**32** 42
	First step pain	Between-group efficacy	Moderate positive**32**,‡ OR: 1.66 (1.00 to 2.76)§		Moderate positive**32** OR: 1.78 (1.07 to 2.96)
		Within-group outcome	1.19 (0.76 to 1.63)**39** **†**	1.74 (1.26 to 2.21)**39 †**	2.93 (2.34 to 3.51)**39**†
	Function	Between-group efficacy	Moderate positive**32** 0.35 (0.10 to 0.60)	Limited positive^42^ 2.39 (1.65 to 3.12)	Limited positive^42^ 0.90 (0.32 to 1.49)
		Within-group outcome	3.47 (2.57 to 4.37),^42^†	4.57 (3.48 to 5.65)^42^†	2.81 (2.02 to 3.61)^42^†
Focused ESWT	Pain	Between-group efficacy	Moderate positive**33** 0.36 (0.11 to 0.61)		
		Within-group outcome	1.33 (0.94 to 1.72)^40 64^ ‡		
	First step pain	Between-group efficacy	Strong positive**33** 41 OR: 1.89 (1.18 to 3.04)	Limited positive^50^ 1.31 (0.61 to 2.01)	Limited positive^50^ 1.67 (0.88 to 2.45)
		Within-group outcome	2.11 (0.75 to 3.48)^43 65^	2.84 (1.94 to 3.73)^50^	3.33 (2.78 to 3.87)^50^
	Function	Between-group efficacy	Moderate positive**33** 0.36 (0.10 to 0.61)		
		Within-group outcome	1.26 (0.99 to 1.53)**33**		
Combined radial and focused ESWT	Pain	Between-group efficacy	Strong positive**32 33** 42 1.08 (0.20 to 1.97)	Limited positive^40 42^ 3.77 (2.82 to 4.72)	
		Within-group outcome	2.72 (1.39 to 4.05)^40 42 63 64^	4.33 (1.12 to 7.55)^40 42 63^	
	First step pain	Between-group efficacy	Strong positive**32 33** 41 OR 1.78 (1.26 to 2.52)		OR 1.95 (1.22 to 3.12)**32** 50
		Within-group outcome	1.79 (0.92 to 2.66)**39** 43 65		3.14 (2.74 to 3.54)**39** 43 50
	Function	Between-group efficacy	Strong positive**33** 42 1.03 (−0.36 to 2.42)		
		Within-group outcome	2.32 (0.16 to 4.49)**33** 42		
Dry needling	Pain	Between-group efficacy	Moderate neutral**35** −0.33 (−0.76 to 0.10)		
		Within-group outcome			
	First step pain	Between-group efficacy	Moderate neutral**35** −0.42 (−0.85 to 0.02)		
		Within-group outcome			
	Function	Between-group efficacy	Moderate neutral**35** 0.11 (−0.31 to 0.54)		
		Within-group outcome			
Wheatgrass	Pain	Between-group efficacy	Moderate neutral**38**,‡		
		Within-group outcome			
	Function	Between-group efficacy	Moderate neutral**38**,‡		
		Within-group outcome			
Calf stretching	First step pain	Between-group efficacy	Moderate neutral**37** −0.39 (−0.80 to 0.03)		
		Within-group outcome			
	Pain	Between-group efficacy	Moderate neutral**37** 0.00 (−0.40 to 0.41)		
		Within-group outcome			
	Function	Between-group efficacy	Moderate neutral**37** −0.24 (−0.65 to 0.17)		
		Within-group outcome			
Low dye taping	First step pain	Between-group efficacy	Moderate positive**22** 0.47 (0.05 to 0.88)		
		Within-group outcome	1.21 (0.77 to 1.66)**22**		
	Pain	Between-group efficacy	Moderate neutral**22** 0.30 (−0.11 to 0.71)		
		Within-group outcome			
	Function	Between-group efficacy	Moderate neutral**22** −0.05 (−0.46 to 0.36)		
		Within-group outcome			
**Interventions with secondary proof of efficacy**					
Plantar fascia stretching	First step pain	Between-group efficacy	Moderate positive**39** 1.21 (0.78 to 1.63)	Moderate positive**39** 0.64 (0.24 to 1.04)	Moderate neutral**39** −0.04 (−0.43 to 0.35)
		Within-group outcome	2.81 (2.27 to 3.35)**39**	3.25 (2.67 to 3.83)**39**	

*Included below are definitions for efficacy and strength of the evidence. An analysis that revealed a significant effect in favour of the intervention was considered a positive effect. The strength of the evidence was rated as strong, moderate or limited based on the number of high-quality trials and whether the trial was adequately powered: strong evidence/positive effect: meta-analysis revealed multiple high-quality trials demonstrated efficacy/a positive effect in favour of the intervention; moderate evidence/positive effect: analysis revealed one high-quality trials demonstrated efficacy/a positive effect in favour of the intervention; limited evidence/positive effect: analysis revealed one high-quality trial, which did not meet the required sample size, demonstrated efficacy/a positive effect in favour of the intervention; strong evidence/neutral effect: meta-analysis revealed multiple high-quality trials demonstrated no efficacy/evidence of no effect; moderate evidence/neutral effect: analysis revealed one high-quality trial demonstrated no efficacy/evidence of no effect; limited evidence/neutral effect: analysis revealed one high-quality trial, which did not meet the required sample size, demonstrated no efficacy/evidence of no effect.

†All effect sizes are reported as an SMD (95% CI) unless otherwise stated, with no pooling of ORs and SMD being possible.

‡Incomplete data or within-group calculations being based on different statistic to between-group, explains apparent discrepancy in results and references used.

§Calculation of effect size using RevMan differs from the reported statistics, so original statistical report was accepted.

ESWT, extracorporeal shockwave therapy; SMD, standardised mean difference.

Of the 51 trials included in the review, 8 RCTs of 9 interventions could be considered for primary proof of efficacy. The nine interventions included radial ESWT,^32^ focused ESWT,^33^ custom foot orthoses,^34^ prefabricated foot orthoses,^34^ dry needling,^35^ magnetised insoles,^36^ calf stretching,^37^ foot taping^22^ and wheatgrass cream.^38^ One trial that compared radial ESWT with plantar fascia stretching was considered for secondary proof of efficacy.^39^

Moderate evidence was found for the efficacy of focused ESWT for overall pain (SMD: 0.36, 95% CI 0.11 to 0.61)^33 40^; strong evidence for first step pain (OR: 1.89, 95% CI 1.18 to 3.04)^33 40 41^ in the short term and moderate evidence of effect for function in the short term (SMD: 0.36, 95% CI 0.10 to 0.61).^33^ No, or minimal side effects were reported in each study, however the procedure was noted to be unpleasant for patients in both study reports and semi-structured interviews.

Significant and positive effects for pain in the short term were revealed for radial ESWT from two papers reporting significant between-group differences, although pooling showed a large effect size with wide CIs that crossed the line of no effect (SMD: 1.64, 95% CI −1.06 to 4.33).^32 42^ Moderate evidence of efficacy was also revealed for first step pain in the long term (OR: 1.78, 95% CI 1.07 to 2.96).^32^ Adverse events were reported as being minimal in each included study. Of note is that one high-quality study found use of radial ESWT without local anaesthetic to be superior to ESWT with prior application of injected local anaesthetic.^43^ The systematic review findings were consistent with the opinions of experts who described the positive effect of ESWT:

There’s enough evidence to suggest that patients with heel pain that have shockwave therapy tend to have less pain on review than the patients that don’t have shockwave therapy. (Expert 14)

Strong evidence was found for the efficacy of custom foot orthoses versus sham for pain in the short term (SMD: 0.41, 95% CI 0.07 to 0.74), although the results were conflicting.^34 44–46^ In addition, small effect sizes were reported for trials by Landorf *et al*,^34^ Oliveira *et al* 45 and Wrobel *et al*,^46^ while a large effect was reported by Bishop *et al*.^44^ Qualitative data confirmed that foot orthoses, without specific reference to custom or prefabricated orthoses, can be used to unload tissues beneath the heel for short-term relief particularly in situations where resting the foot is not feasible. No trials of heel cups were found, and they did not feature in the qualitative data.

One study that met all the quality and power criteria evaluated the efficacy of low dye taping and sham ultrasound versus sham ultrasound alone.^22^ There was moderate evidence of primary efficacy at 1 week for ‘first-step’ pain in favour of low dye taping (SMD: 0.47, 95% CI 0.05 to 0.88). Some patients expressed the positive role for taping to alleviate symptoms:

I think the strategies that I was given in the short term were helpful (eg, taping and stretching). (Patient 12)

In addition, experts revealed that taping is a first-line treatment that is an effective method to reduce pain in the short term and enhance patient confidence. Some experts used taping to predict the efficacy of foot orthoses:

If I tape them and their symptoms decrease and then I can say—okay, I think I can replicate what the tape is doing with either shoes or orthoses. (Expert 13)

There was moderate evidence of large effect that plantar fascia stretching is superior to radial ESWT for first step pain in the short term (SMD: 1.21, 95% CI 0.78 to 1.63)^39^ and of medium effect in the medium term (SMD: 0.64, 95% CI 0.24 to 1.04) but not in the long term (SMD: −0.04, 95% CI −0.43 to 0.35). The sample was mainly people presenting with acute PHP, and this finding complements expert reasoning well, with there being clear guidance to continue stretching targeting the plantar fascia and related structures in a variety of ways throughout rehabilitation:

They can feel an immediate response, and there seems to be some adaptation to this stretching, but again I would say this is definitely not the cure for this. (Expert 4)

### Expert interviews

Interview transcript analysis revealed 6 themes and 30 subthemes. The first two themes concerned diagnosis and patient education (table 2) and particularly influenced the core approach (figure 2), along with the findings on stretching in the ‘rehabilitation’ theme alongside ‘factors underlying management’ and ‘specific interventions’ (online supplemental file 1—expert reasoning results), which had particular influence on the stepped approach to care (figure 1). ‘Perceptions of evidence’ (online supplemental file 1) was the final theme and informed the recommendations made concerning application of specific interventions for patients recovering too slowly or not responding at all (figure 1).

**Table 2:** Qualitative analysis of expert interview data pertaining to diagnosis and patient education

Findings Illustrative quotes		
**Theme 1: diagnosis**		
History		
Overview of key elements to explore	High repetitive use versus change of use; mechanical history essential to establish; rest-activity balance important; typically insidious onset but important to check injury; importance of ruling out other causes (inflammatory, tendinopathy and neuropathic masqueraders); reduction with movement.	Q: If you have had an increase in weight, and that’s why you’ve got your heel pain, then that’s probably a point of discussion.^11^ Q: Was there an acute incident, to rule out fat pad contusion?^10^ Q: Those for whom it is part of a systemic arthritis are generally younger because seronegative arthropathy is often in a younger age group.^9^
Relative importance	Key factor in establishing diagnosis; sets priorities for physical and imaging.	Q: The primary diagnosis, when you first see someone, is generally clinical.^14^ Q: History essentially nails the diagnosis.^8^ Q: Only time I would really go for ultrasound would be if I am suspecting a tear or a rupture.^8^
Presentation of pain	am pain pathognomic; first step pain most informative; pain after inactivity; well-localised to medial-inferior heel; worse at start and at end/after aggravating activity; description as sharp at worst versus ache at other times; mechanical versus psychosocial.	Q: Very localised pain at the medial tubercle of the calcaneum.^3^ Q: First steps in the morning … after sitting for a long time … very good indication.^4^ Q: …Out of bed in the morning it’s like walking on shattered glass or walking on needles and pins.^2^
Subgroups	Lean versus high BMI; highly active versus relatively inactive; profession may indicate risk; overweight and standing job a particular risk.	Q: One group is those with high BMI, and they stand up at work 7–8 hours a day, and other group is the lean runner maybe doing too much too soon.^2^ Q: You also have these people standing a lot standing 8 hours a day at their working place.^6^ Q: Take a good history … profession and their sport and fitness regime per week.^8^
Examination		
Physical testing	Palpation at inferior medial heel (PF origin) or close to; check for ruptures; look for compensation movements; calf flexibility a key element.	Q: I could leave out the US scan, but I would always do a through history on the patient, and palpate the area.^4^ Q: Also check their calf inflexibility.^8^ Q: Activate windlass mechanism to see if plantar fascia tightens.^2^
Structures of interest	Consider all aspects of fascia; consider old injuries (medial, lateral, distal); tendinopathy, neuropathy and bone key differentials.	Q: Squeeze the calcaneus … if that causes some discomfort then I assume that there’s probably some bony oedema.^11^ Q: Dorsiflex the hallux, dorsiflex the ankle … start distally and palpate down the plantar fascia and work towards its origin.^10^ Q: Do some physical testing, I rule out other tendinopathy in the area.^5^
Imaging		
Decisions to use imaging	Use is confirmatory not diagnostic; availability and specialty may dictate use; subordinate to history and examination.	Q: US helps look at specific portion of fascia; check for tears and fibromas.^8^ Q: If I do an US, diagnostic US in someone, I cannot tell them that they have PF, that’s how crazy it is.^5^ Q: I think a lot of people go wrong, they look at imaging and try diagnosing, but really it comes down to the subjective features and the clinical features.^4^
Perceptions of utility	Sensitivity and specificity questionable; MRI unclear versus useful for bone oedema; US useful to exclude tears and lumps; US dimensions more useful than Doppler; changes likely bilateral even if unilateral pain.	Q: The more imaging work I do the more I realise that there are other things that are going on.^7^ Q: The other advantage is that MRI you can start to see there is inflammation, say, in the facets of the subtalar joint. You can start to see if there is some bone oedema.^12^ Q: For the more resistant or long-term cases, then an MRI would be my investigation of choice.^14^
**Theme 2: patient education**		
Importance of patient education	Education key to prevent recurrence; importance as for all musculoskeletal conditions; aetiology must be understood; key to patient engagement, self-management and treatment success; treatment rationale important for patient to learn; requires mixed communication methods; under-researched area; focus on key pain driver; relate to specific patient presentation; include physical and non-physical factors; reassure about positive long-term prognosis.	Q: If we leave these maladaptive beliefs unchecked, then it will lead to chronicity.^3^ Q: If they understand what the problem is and the course of it then it’s easier to have compliance.^6^ Q: If you don’t address those issues then it could be that if you remove your orthotics, stop taping or stopped your stretching or whatever, the pain is just going to come back so that’s where the education side of things is really important.^7^ Q: Overarching thing is that you’ve got to individualise it for the person.^11^
Teaching about load management	A primary goal of treatment; consider both static and dynamic weight-bearing load; change of overall load a risk factor for exacerbation; focus on function by unbundling erroneous patient perception of pain and pathology link; useful for patient to understand and self-manage a stepped approach to load increase with guidance; weight loss and associated metabolic factors poorly understood but impact on load management approach; need to address weight sensitively; therapists may not have weight management skills; key therapeutic effect mediator.	Q: Load tolerance is probably a good way to describe the key treatment.^3^ Q: Obviously, there’s more load if you’ve got more weight, so if we can reduce that it’s going to help reduce the load on the plantar fascia.^1^ Q: Get down to business and talk to him about his training programme and talk about how many miles they do a week.^2^
Advice on footwear	Comfort is key modification guide; consider softness, shock absorption, rearfoot to forefoot drop and support; new shoes need to be socially acceptable; can use to offload tissue.	Q: Getting patients into good footwear that has a small heel on it, because it takes the tension off the calf muscle and therefore the fascia, and having good cushioning or shock absorbency, are some key factors.^14^ Q: I don’t think minimalist (footwear) is made for everybody.^13^

BMI, body mass index; US, ultrasound.

### Patient survey

Forty people responded to the online survey with the Framework analysis resulting in one overarching theme of ‘patient experience’ with eight subthemes (table 3). The quantitative check showed 95% of 266 specific treatment components or descriptions of management approaches mentioned in the patient responses were consistent with the core approach initially derived from the review and expert interviews therefore indicating good agreement between the evidence, experts and patient experience.

**Table 3:** Framework analysis of 40 patient survey responses yielding 8 subthemes

Theme 1: patient values		
Subtheme Findings Illustrative quotes		
Thoughts on condition cause	Foot arch height; age; activity pattern; new load increase; long periods weight bearing; standing on hard surfaces; minimally supportive footwear; limb length asymmetry; rapidly changing load; altered gait; altered movement due to other conditions.	Q: Walking on the outside edge of my foot when I was having pain in my second toe (PN). Q: Heel spurs, arthritis. Q: Long shifts on my feet in facilities with hard floors. Q: Excess loads with inadequate progression. Q: A number of contributory factors which is why is occurred now.
Thoughts on pathology	Tissue irritation; degeneration; inflammation; tearing; inadequate tissue capacity; contracture.	Q: Tissue band has become irritated through age/overuse. Q: It feels like it is tearing. I think I have torn a ligament. Q: Inflamed damaged PF which needs to heal/repair. Q: Struggling to cope with the demand and non adapted tissue. Q: Tendon contracture is wanting to happen all the time.
Expectations	More information; quick recovery-unrealised; exercise programme, especially foot strengthening; pain elimination; access to orthoses; specific treatments; better explanation of treatment/condition and causes.	Q: Expected to get a steroid shot and was hoping for deep tissue manipulation to break down the scaring or thickening tissue. Wasn’t offered. Q: I assumed wrongly I would need insoles. I expected to be back on my feet within a few weeks (very optimistic). Q: As swift a recovery as possible, relief from the pain and programme of exercises to treat.
Needed improvements	Facilitation of earlier recognition by patients; better communication as adherence promotion. Intervention strategy for pain; easier access to, and more information on, specific treatments; standardised treatment across sectors; clarity of treatment and expectations; reduced waiting times.	Q: Better understanding of symptoms and types of patients prone to PHP. Q: More explanation for the mechanism of the symptoms in order to motivate me to do the exercise. Q: Get rid of the pain forever. Q: Standardised treatment from NHS across the country. I’ve gone private as Dr can’t refer.
Strengths of management	From no strengths to positive experiences; fast decisions; specific interventions; clear plan; individual preferences accounted for; detailed explanation; specific interventions.	Q: Range of options considered and clearly explained. Q: Spent time explaining in detail the condition/cause/treatment.
Experience	Restricted activity; intermittent severe pain; reduced exercise; altered activity; morning pain; painful; emotionally affected; large impact on ADL; long, uncertain recovery.	Q: It restricted the activities I wished to carry out. Q: It’s very painful under my heel when I get up in the morning. Q: Miserable 6 months. Had a huge impact on daily activities. Q: Very long process and uncertain outcome.
Key information	Time course of recovery; self-management advice; how pain relief works; long-term effects; explanation of what was not done; unsure; statistics on usual timescales for effects.	Q: What can I do to reduce my pain and improve function? Q: Will pain reliever actually address the issue or just mask the pain? *Q: When* they could make the pain go away? Q: Expected outcome at the end of rehab.
Sources of information	Range of online methods predominated; clinicians, friends, magazines; lack of clear guidance.	Q: I can google it all day, and there isn’t much out there. Q: Patient groups on Facebook aren’t even very helpful, because everyone using them hasn’t found relief. Q: Online forums, confusing as everyone’s cause is different therefore treatment different.

NHS, National Health Service; PHP, plantar heel pain.

### Best practice guide

The BPG was produced from synthesis of quantitative (review) and qualitative (expert interviews and patient survey) data. A core approach was determined (figure 2) prior to stepped care for patients progressing slowly, or inadequately (figure 1). The core approach consists of the best evidence-based interventions of plantar fascia stretching and low dye taping complemented by an individualised education approach. All recommended core approach components should be used simultaneously for approximately 4–6 weeks before consideration of adjunctive interventions such as ESWT or orthoses. Expert interviews strongly emphasised the need to implement this education and self-management approach prior to applying the interventions identified to have strong evidence when pain remains unchanged from baseline (table 2). The timelines were derived from the qualitative components and reflect the time required for someone to respond to the core approach, but recognise a need to adjust these timelines based on individual circumstances. In line with the planned method, inclusion of each intervention was determined by the systematic review and the mode of application, including when and how, from our expert interviews.

The nature of the condition is that you need to be doing a range of things, but all together for a sustained period of time. (Expert 14)

Of the three components of the core approach, taping and plantar fascia stretching should be universally applied and were annotated as ‘DO’ in figure 2. Nearly all trials included education, explicitly or implicitly in each intervention package that was applied, although education in isolation has never been specifically tested as a sole intervention. Education was not tested for efficacy but was recommended by experts and appreciated by patients, therefore it is suggested as a necessity for effective treatment. However, trials assessing interventions with and without education would help to strengthen this recommendation. Individualised decisions about education content are needed and were annotated as ‘DECIDE’ in figure 2. Education content had four subareas. For load management, the key issues were to reduce overall tissue compressive load by breaking up long periods of static loading such as standing and reducing injurious compressive and stretch-related dynamic loading from activities such as running in the more active population. For pain education, clarity about the meaning of pain and its relation to tissue state needs to be clearly understood, alongside realistic expectations of the prognosis being good but resolution likely to be slow. Techniques such as pain-monitoring were strongly recommended. The possible impact of other presenting long-term conditions and an elevated body mass need to be addressed. Finally, the requirement for footwear to be supportive, comfortable, incorporate a rearfoot to forefoot drop and be socially acceptable is required with specific advice to avoid barefoot walking and flat, unsupportive footwear until symptoms have entirely resolved.

the number one thing is educating people with PHP (sic) to have some understanding about the most likely reason they felt the pain, and then based on that, the key things that they need to do long term (Expert 9)

Where the core approach is only partially successful or taking >6 weeks to yield optimal outcomes for a patient, adjunct interventions are recommended based on the strength of the quantitative evidence and expert reasoning. The use of simple but validated patient-reported outcome measures, such as a global rating of change scale^47^ or equivalent may help guide these decisions. Patient experience had little influence on this section, with adjunctive interventions such as ESWT and orthoses having less prominence in responses. The primary recommendation, included as an intervention due to strong review evidence and applied according to expert interview evidence, was that ESWT—applied using either radial or focused approaches—should be applied if people with PHP are not deriving optimal benefit from the core approach as it has the strongest overall evidence.

Where the core approach and ESWT are still not successful, the stepped care approach recommended custom orthoses and if still not successful—as marked by an X in figure 1—then experimental approaches may be tried, although expert reasoning suggests that a return to the core approach and repeat application ensuring good accuracy and adherence is also a feasible fourth-line approach. Where interventions have been tried and shown to be ineffective in the literature, they should not be used except in formal trials, whereas inadequately tested interventions with no primary or secondary proof of efficacy such as injection therapy—where the evidence is inadequate or not present—may be considered, again preferably via RCT or with structured evaluation.

## Discussion

We synthesised high-quality RCTs, elicited expert clinical reasoning and surveyed patients to produce this BPG. Our work meets the majority of the relevant Agree II criteria,^48^ and the high-quality criteria previously reported for guideline development.^49^

The BPG defines a core approach (figure 2) to management which consists of the simple but active, supported self-management interventions of plantar fascia stretching^39^ and taping (labelled ‘DO’ in the figure) to support the plantar fascia,^22^ alongside less well-defined educational interventions (labelled ‘DECIDE’ in the figure). The interviews with experts gave clear direction that this education should encourage:

- individual assessment;
- footwear advice to ensure comfort in shoes that allow a small rearfoot to forefoot drop while also considering social acceptability to improve adherence;
- load management to break up long periods of static loading or problematically rapid training changes in more athletic populations;
- support to address comorbidities such as type 2 diabetes (online supplemental file 1);
- teaching patients the parameters required to self-monitor the pain response to activity and how to interpret pain with respect to tissue damage in order to allay fears of long-term consequences.

The educational delivery should adopt a realistic tone as recovery may take several weeks or months but stress the positive prognosis, a recommendation that came through strongly in the expert interviews.

### The role of ESWT

The systematic review showed ESWT had the best evidence of modalities that have been evaluated. It is typically used for people with non-resolving, persistent symptoms.^32 33 40–42 50^ As ESWT is inferior to stretching for acute symptoms,^39^ and based on the clinical reasoning elicitation, ESWT is recommended in the BPG when patients are failing to recover optimally using the core approach (figure 1). ESWT had the best evidence of any adjunctive treatments, and has large combined study cohorts, with minimal documented adverse events, demonstrating positive efficacy in the short term, medium term and long term for most patient-reported outcomes. Focused shock wave is applied so that the peak intensity is deep to the skin thus being targeted directly at the lesion and RCTs showed moderate positive short-term findings on pain of large effect.^33 40^ Radial ESWT results in peak intensity at the surface and showed moderate positive effects, again of large effect at all time points for patient-reported outcomes. When pooled as single arms of multiple studies, effect size magnitude for ESWT is large, with these data informing clinicians about the likely real-world outcome, rather than illustrating comparative effect.

The ESWT evidence has some limitations that affected synthesis of related trials. Continuous outcomes that evaluated overall pain, first step pain or function were often reported at baseline but were presented as the overall success rate with regard to heel pain at follow-up. For example, Gerdesmeyer *et al* 32 and Gollwitzer *et al* 33 41 presented first step pain, pain while doing activities and pain after application of a dolorimeter at baseline on a continuous scale. However, at follow-up they reported scores on a dichotomous scale by defining success as >60% reduction in heel pain from baseline for at least two out of three measures that evaluated pain. While it might be desirable to label participant’s outcome as either a ‘success’ or ‘not a success’ in response to an intervention, this approach has disadvantages. For example, participants that are close to but on opposite sides of the clinical cut-off point for success are labelled as being categorically different rather than being similar.^51^ Our synthesis was strengthened by the effect sizes being large, and the presence of enough adequately powered trials of high quality to determine efficacy. These guide practice where patients are not improving quickly enough or failing to respond to the core intervention (figure 1). High-quality effectiveness studies are warranted, particularly in people with PHP who are not responding to the core approach as the majority of published work has been efficacy studies, and cost-effectiveness has not been evaluated.

### When patients do not get better from the core intervention or ESWT

Where patients do not respond to core treatment or ESWT then other options are available. Custom orthoses can be considered based on positive evidence of moderate strength and lower effect size than ESWT^34^ for short-term outcomes (figure 1). This progression is extrapolated from expert interviews and systematic review findings, rather than from trials including failed previous treatment of a specific kind as explicit inclusion criteria.

Prefabricated or custom orthoses are often prescribed for PHP. However, none of the trials included in our review used the same orthosis. All differed in the prescription process, casting technique, shell material, top-covers and modifications, thus limiting trial comparison. The prescription of foot orthoses in clinical practice, whether customised or prefabricated, commonly involves a process of both education and orthosis modification in an attempt to optimise the dose and biomechanics.^52^ No included RCTs followed this process, possibly limiting efficacy of orthoses. Furthermore, prefabricated orthoses, as used in the included trials, were shown to be ineffective. Therefore, it can be recommended that a single orthosis prescription is not used for all presenting patients, an assertion supported by expert opinion in this study. Given the contrast with custom orthoses, it may be that having a range of prefabricated orthoses may be a suitable strategy so that prescription can be individualised. This approach would be a priority for future cost-effectiveness trials, given the lower cost compared with casting or scanning.

*Dry needling* had a positive effect on pain and function in the short term. Cotchett *et al* 35 revealed a small but significant effect for pain and function based on a statistical approach that included an analysis of covariance. However, our data analysis revealed evidence of no effect, which might have been revealed because comparison of final values included between-participant variability such as differences in baseline pain scores. Based on the latter analysis, dry needling can be considered to have neutral evidence of effect but could be considered as an adjunct intervention to the core approach, with lower priority than orthoses. Trigger point dry needling is also associated with minor adverse events such as needle site pain and to a lesser extent minor bruising. Findings from the interviews indicated that dry needling is not a first-line treatment but may be considered to influence pain and muscle tension when combined with other interventions.

*Corticosteroid and platelet-rich plasma injection* therapy was very carefully assessed, both in the trials and the semi-structured interviews as this is a commonly used intervention. Interestingly, although injection therapy using steroid or platelet-rich plasma is readily amenable to placebo or sham administration, no such RCTs have been performed. It was judged that insertion of a needle with no subsequent drug injection into the plantar fascia could not be considered a no-treatment placebo, as it was likely to have an effect given the findings of Cotchett *et al* 35 with dry needling. Furthermore, injection of any drug or saline would also have chemical and physical effects, which could lead to confounded RCT results. This is an intervention for which placebo control is readily achievable and represents a priority for future research—perhaps in patients where ESWT has failed to yield optimal results.

*Resistance exercises* of the affected area and limb are often effective as part of first-line care for chronic musculoskeletal problems such as osteoarthritis of the hip or knee and common tendinopathies.^53–55^ While there is moderate evidence for stretching the plantar fascia, our stringent systematic review could not identify evidence in favour of more comprehensive exercise approaches. Furthermore, the expert interviews did not provide a theme on hidden efficacy and was divided on whether such resistance exercises are useful or not (online supplemental file 1). Trials currently in development may clarify this issue in the future.^56^

### Agreement between SR, expert interviews and patient survey

Information obtained from the three methods used to formulate the BPG was generally consistent. For example, the need for positivity when discussing prognosis was expressed by experts and mirrored in the patient survey responses. Furthermore, there was strong agreement between the patient survey findings and the content of the core intervention while expert interviews and the SR were in agreement about the efficacy of the various interventions. The agreement was also good where the SR evidence was unclear. For example, there is an absence of high-quality trials of progressive strengthening while the expert interviews showed very divergent views on likely efficacy.

### How to use this BPG

Taking into consideration each patient’s past treatment history and experience, the BPG can guide patients and clinicians. It can also inform healthcare commissioners and help design future research.^57^ Patients having access to summary resources such as figures 1 and 2 should reduce some of the inconsistency they report when seeking guidance from internet and other resources. Commissioners may consider funding the necessary resources—such as ESWT devices. Audit, monitoring and checklist tools do not yet exist, but should be developed locally and may be a useful stimulus to intervention adoption. Finally, future research may be guided by consideration of the gaps in the evidence base and the expert views on research priorities identified by our work (online supplemental file 1).

### Strengths and limitations

The overall quality of the research in the PHP field is low. Of the 362 trials evaluating any intervention for PHP that were assessed for quality using the PEDro and RoB criteria, only 51 met the inclusion criteria. These stringent quality criteria without topic restriction were used in order to strengthen recommendations, meaning that the highest quality recent review included 20 trials,^9^ which we excluded due to low quality or high RoB. Additionally, this same review did not include 35 studies included as part of our more comprehensive review. Trials that met the PEDro cut-off score were further evaluated for bias against the standards outlined by the Cochrane Collaboration tool for assessing RoB^15^ and broadly demonstrated equivalence. We believe that future efficacy and effectiveness trials which are not of high quality, for example, meeting the inclusion criteria used in our research, should no longer be funded or performed on ethical grounds (Helsinki statement—section on efficacy).^58^ Arguably, new treatments should complete intervention development packages prior to undertaking an RCT as per the Medical Research Council complex interventions framework,^59^ with our mixed-methods study being consistent with the initial phases of these guidelines (online supplemental file 1).

Finally, the perceptions and experience of people with PHP identified gaps in the education and treatment they had received. Consistent with recent qualitative work,^60^ participants highlighted a poor understanding of their condition, including the underlying pathology, causal factors, efficacy of various treatments, mechanisms behind interventions, knowledge regarding expectations of improvement and how to progress when treatment is failing. Clinicians must consider how education is being delivered to their patients to ensure there is clear guidance on treatment and behaviour change, fears are allayed and that learning is checked rather than simple information provision. In fact, it is likely that interventions for clinicians, which are informed by behaviour change theory^61^ may need to be developed to ensure optimal evidence translation. In summary, we have identified a BPG which should inform and guide patient care, but there is further work to be done to ensure updated treatment approaches so that patients can optimally benefit.

**What is already known**

- Plantar heel pain is common and can have a negative impact on physical and mental health.
- Existing guidelines lack clear, high-quality recommendations for treating people with plantar heel pain.

**What are the new findings**

- A systematic review and meta-analysis, supplemented with expert clinical reasoning and patient values revealed stretching, foot taping and educational interventions are part of the core approach for people with plantar heel pain.
- A core and stepped approach to the management of people with plantar heel pain was formulated, which will prove immediately useful to clinicians who treat, and to those who suffer from, plantar heel pain.

10.1136/bjsports-2019-101970.supp2 Supplementary data
