# Supplementary material for: Management of plantar heel pain: a best practice guide informed by a systematic review, expert clinical reasoning and patient values
Source: Br J Sports Med. 2021 Mar 30;55(19):1106–18. doi: 10.1136/bjsports-2019-101970 (PMC8458083; doi:10.1136/bjsports-2019-101970)
Supplement: Supplementary data [file bjsports-2019-101970supp001.pdf]

Management of plantar heel pain: a best practice guide synthesising systematic review with expert clinical reasoning and patient values

SUPPLEMENTARY FILE 1

MATTHEW COTCHETT

Contents

1. Checklists 2
   1.1. GRAMM (Good Reporting of A Mixed Methods) Study[1] Checklist 2
2. Methodological appendices 3
   2.1. Database search 3
   2.2. Database search – MEDLINE – October 2019 5
   2.3. Characteristics of experts 6
   2.4. Topic guide to facilitate interview with experts 7
   2.5. Patient survey 7
3. Qualitative analysis of expert interviews 11
   3.1. Qualitative analysis of interview data pertaining to management principles 11
   3.2. Qualitative analysis pertaining to diagnosis and patient education 12
   3.3. Qualitative analysis of interview data pertaining to specific interventions 12
   3.4. Qualitative analysis, perceptions rehabilitation and exercise 16
   3.5. Qualitative analysis of interview data pertaining to perceptions of the evidence 17
4. Qualitative analysis of patient survey 18
5. Supplementary systematic review results 18
   5.1. Characteristics of included studies 18
   5.2. Quality analysis scores 50
   5.3. Short term results for pain and function 54
   5.4. Medium term results for pain and function 88
   5.5. Long term results for pain and function 98
   5.6. Interventions with neutral evidence of efficacy 103
   5.7. Forest plots 106
   5.8. Risk of Bias 2 108
   5.9. Risk of Bias 2: Risk of Bias – Support for Judgements 109
6. Limitations 141
   6.1. Quality analysis 141
   6.2. Data analysis 142
   6.3. Sample size bias 144
   6.4. Sample size considerations 145
   6.5. Additional limitations 146
7. Implications for further research 147

1. Checklists

1.1. GRAMM (Good Reporting of A Mixed Methods) Study[1] Checklist.

ITEM	Present?	PAGE(S)
1. Describe the justification for using a mixed methods approach to the research question	Yes	4
2. Describe the design in terms of purpose, priority and sequence of methods.	Yes	10-12
3. Describe each methods in terms of sampling, data collection and analysis.	Yes	10-12
4. Describe where integration has occurred, how it has occurred and who has participated in it.	Yes	12
5. Describe any limitation of one method associated with the presence of the other method.	Yes	None noted
6. Describe any insights gained from mixing or integrating methods.	Yes	Fig 2, 3 and 4

2. Methodological appendices

2.1. Database search

Database search – MEDLINE – October 2019

Medline

"plantar fasciitis" OR "plantar fasciopathy" OR "subcalcaneal pain syndrome" OR "plantar heel pain syndrome" OR "jogger's heel" OR "heel pain" OR "painful heel" OR "chronic plantar heel pain" OR "heel spur syndrome"

CENTRAL/Web of Science/open grey/biosis previews

"plantar fasciitis" OR "plantar fasciopathy" OR "subcalcaneal pain syndrome" OR "plantar heel pain syndrome" OR "jogger's heel" OR "heel pain" OR "painful heel" OR "chronic plantar heel pain" OR "heel spur syndrome"

CINAHL/sportdiscus:

"plantar fasciitis" OR "plantar fasciopathy" OR "subcalcaneal pain syndrome" OR "plantar heel pain syndrome" OR "jogger's heel" OR "heel pain" OR "painful heel" OR "chronic plantar heel pain" OR "heel spur syndrome" (TITLE) OR "plantar fasciitis" OR "plantar fasciopathy" OR "subcalcaneal pain syndrome" OR "plantar heel pain syndrome" OR "jogger's heel" OR "heel pain" OR "painful heel" OR "chronic plantar heel pain" OR "heel spur syndrome " (ABSTRACT)

EMBASE:

'plantar fasciitis' OR 'plantar fasciopathy' OR 'subcalcaneal pain syndrome' OR 'plantar heel pain syndrome' OR 'jogger heel' OR 'heel pain' OR 'painful heel' OR 'chronic plantar heel pain' OR 'heel spur syndrome'

PROSPERO:

'plantar fasciitis' OR 'plantar fasciopathy' OR 'subcalcaneal pain syndrome' OR 'plantar heel pain syndrome'

Controlled-trials.com:

http://www.isrctn.com/search?pageSize=20&sort=&page=1&q=%22plantar+fasciitis%22+OR+%22plantar+fasciopathy%22+OR+%22subcalcaneal+pain+syndrome%22+OR+%22plantar+heel+pain+syndrome%22+OR+%22jogger%27s+heel%22+OR+%22heel+pain%22+OR+%22painful+heel%22+OR+%22chronic+plantar+heel+pain%22+OR+%22heel+spur+syndrome%22&filters=&searchType=basic-search

http://apps.who.int/trialsearch/:

search for: "plantar fasciitis" OR "plantar fasciopathy" OR "subcalcaneal pain syndrome" OR "plantar heel pain syndrome" OR "jogger's heel" OR "heel pain" OR "painful heel" OR "chronic plantar heel pain" OR "heel spur syndrome"

Clinicaltrials.gov

https://clinicaltrials.gov/ct2/results?term=%22plantar+fasciitis%22+OR+%22plantar+fasciopathy%22+OR+%22subcalcaneal+pain+syndrome%22+OR+%22plantar+heel+pain+syndrome%22+OR+%22jogger%27s+heel%22+OR+%22heel+pain%22+OR+%22painful+heel%22+OR+%22chronic+plantar+heel+pain%22+OR+%22heel+spur+syndrome%22&Search=Search

2.2. Database search – MEDLINE – October 2019

Edit Search

Search Name: Best Practice October 2019_MEDLINE

Comment:

Save Cancel

Set	Search Statement	Annotations	Insert	Edit	Delete
1.	"plantar fasciitis".mp. [mp=title, abstract, original title, name of substance word, subject heading word, floating sub-heading word, keyword heading word, protocol supplementary concept word, rare disease supplementary concept word, unique identifier, synonyms]				
2.	"plantar fasciopathy".mp. [mp=title, abstract, original title, name of substance word, subject heading word, floating sub-heading word, keyword heading word, protocol supplementary concept word, rare disease supplementary concept word, unique identifier, synonyms]				
3.	"subcalcaneal pain syndrome".mp. [mp=title, abstract, original title, name of substance word, subject heading word, floating sub-heading word, keyword heading word, protocol supplementary concept word, rare disease supplementary concept word, unique identifier, synonyms]				
4.	"plantar heel pain syndrome".mp. [mp=title, abstract, original title, name of substance word, subject heading word, floating sub-heading word, keyword heading word, protocol supplementary concept word, rare disease supplementary concept word, unique identifier, synonyms]				
5.	"jogger's heel".mp. [mp=title, abstract, original title, name of substance word, subject heading word, floating sub-heading word, keyword heading word, protocol supplementary concept word, rare disease supplementary concept word, unique identifier, synonyms]				
6.	"heel pain".mp. [mp=title, abstract, original title, name of substance word, subject heading word, floating sub-heading word, keyword heading word, protocol supplementary concept word, rare disease supplementary concept word, unique identifier, synonyms]				
7.	"painful heel".mp. [mp=title, abstract, original title, name of substance word, subject heading word, floating sub-heading word, keyword heading word, protocol supplementary concept word, rare disease supplementary concept word, unique identifier, synonyms]				
8.	"chronic plantar heel pain".mp. [mp=title, abstract, original title, name of substance word, subject heading word, floating sub-heading word, keyword heading word, protocol supplementary concept word, rare disease supplementary concept word, unique identifier, synonyms]				
9.	"heel spur syndrome".mp. [mp=title, abstract, original title, name of substance word, subject heading word, floating sub-heading word, keyword heading word, protocol supplementary concept word, rare disease supplementary concept word, unique identifier, synonyms]				
10.	1 or 2 or 3 or 4 or 5 or 6 or 7 or 8 or 9				

Save Cancel

2.3.Characteristics of experts

PG+Age (years)	Academic	Clinical			Academic/Clinical			Patients seen	Publications
		Public	Private	Both	Public	Private	Both	(average)	(average)
5-10	1					1		9 per month	56
>10	2	1		1	1	5	1	10 per month	46
Total	3	1		1	1	6	1	9.5 per month	51

2.4. Topic guide to facilitate interview with experts

2.5. Patient survey

PHP – topic guide summary

Context and background

- Introduction, study purpose, interview procedure, confidentiality and consent
- Interviewees background and experience
- Comment on process of diagnosis of PHP? Discussion about imaging. Special tests sued?

Management of PHP

- Most important aspects of a patient's presentation when it comes to managing PHP?
- How would you manage a patient with PHP - which treatment modality would you start with? Why?
- Enquire about each treatment: Which patients benefit most? Duration of treatment? Would you use this in combination with anything else? Comment on the strength of evidence?
- If this doesn't work, what would you try next? Any specific criteria of when to use next modality
- "have you changed the way you treat PHP from past experience and why?"
- 'how do you feel about treating PHP (confident/unsure) and why'
- "are there things you do not do, but are aware of, that may be relevant?"
- "what do you do if a patient is not getting better as expected?"
- "what is your last ditch approach and why?"
- At what point might you refer for surgery? Which patients benefit most?

Introduction of online prompt tool

as you know one of the aims of this project is to produce a care pathway, so what I'd like you to do is to arrange these different modalities on the online timeline in the order you think is most appropriate and talk me through your thought process as you do it.

Other factors:

- Factors affecting patient adherence/compliance and how this influences your management decisions?
- Costs of treatment and how they influence management
- Are you of aware of any pathways on PHP? Do you use them, and what is your experience?
- Views on current level of evidence regarding management of PHP?
- How is evidence-based medicine incorporated into your practice?
- How can evidence translation in practice be improved? What are the barriers preventing evidence translation into practice? Any resources which help you?
- How important is the consideration of psychosocial factors in the assessment and management of people with PHP?
- What from your perspective, is the nature of any relationship between psychosocial factors and adherence to treatment plans for PHP?
- what are your thoughts on markers for PHP severity?
- why do you think there are so many attempted interventions for PHP?
- I am going to be provocative now, and ask what you make of the statement
    - "we should stop intervention research and go back to basics like aetiology and pathology?"
- what is your opinion of the quality of PHP research and why?
- do you think there are conflicts of interest in the research and in the delivery of services?
- Is there anything else from your clinical experience you feel is important?
- Expansion questions as determined by interviewee.

Thanks and conclusion

Queen Mary University of London A survey of people with experience of PHP to inform best practice.

Welcome

We would like to invite you to be part of this research project. You should only agree to take part if you want to, and it is entirely up to you. If you choose not to take part there won't be any disadvantages for you and you will hear no more about it.

Before you decide it is important that you understand why the study is being done and what it would involve. Please take time to read the following information carefully before you decide to take part; this will tell you why the research is being done and what you will be asked to do if you take part. The researcher will happily go through the information sheet and answer any questions you may have.

If you decide to take part you will be asked to confirm on the following section to say that you agree. You are still free to withdraw at any time and without giving a reason.

Queen Mary University of London A survey of people with experience of PHP to inform best practice.

Participant Information Sheet

What is the purpose of the study and why have I been invited to take part?
You have been invited to take part because you have been diagnosed with plantar heel pain. We are keen to find out your views on some aspects of PHP management. This study aims to determine patients' perspectives about PHP management in order to guide best practice.

Do I have to take part?
No. Whether or not you join the study is up to you. If you agree to take part, then simply completing the survey will act as consent to participate.

Our contact e-mail is d.morrissey@qmul.ac.uk

What happens if there is a problem?
If you have any questions or concerns about any aspect of the study or about the way of the study was conducted please, in the first instance, contact the researcher responsible for the study. They will try their best to answer your questions. If this is unsuccessful, or not appropriate, or you wish to make a formal complaint, please contact Hazel Covill, the Secretary at the Queen Mary Ethics of Research Committee, Room W104, Queen's Building, Mile End Campus, Mile End Road, London or research-ethics@qmul.ac.uk tel: 020 7882 7915/6947

Will my taking part in this study be kept confidential?
Yes. We will follow ethical and legal practice and all information about you will be handled in confidence. Any data, which may identify you, will be held on secure servers. The research team will be the only ones allowed

access to this information. By consenting to be involved in this study you give the research team permission to access your information. All information collected about you during the study and after completion will be kept strictly confidential and all researchers of the research team will abide by the Data Protection Act 1998 and the rights you have under this Act.

What happens if I don't want to carry on with the study?

You are free to withdraw at any time, without giving a reason. A decision to withdraw at any time, or a decision not to take part, will not affect you. If you withdraw from the study, we will stop any future data collection, but we will need to use the anonymised data collected up until your withdrawal.

What will happen to the results of the study?

The results of the study will be analysed and presented as written work, presentation material and possible publication in a peer reviewed journal. Your data will be anonymized.

Queen Mary University of London

A survey of people with experience of PHP to inform best practice.

From your experience:

1. What do you feel could be improved in the management of PHP?
2. What were the strengths of how your PHP was managed?
3. How would you describe your experience of living with PHP?
4. Why do you think you developed PHP?
5. What do you think is happening in your heel?
6. What key things did you want to know from the clinician looking after your care?
7. What were your treatment expectations?
8. What sources have you found most useful to get information about PHP?

What do you think of the best practice infographics below? These are a guide for clinicians on which we are keen to get your views.

In this scenario, shock wave therapy is recommended for people who are not getting better quickly enough.

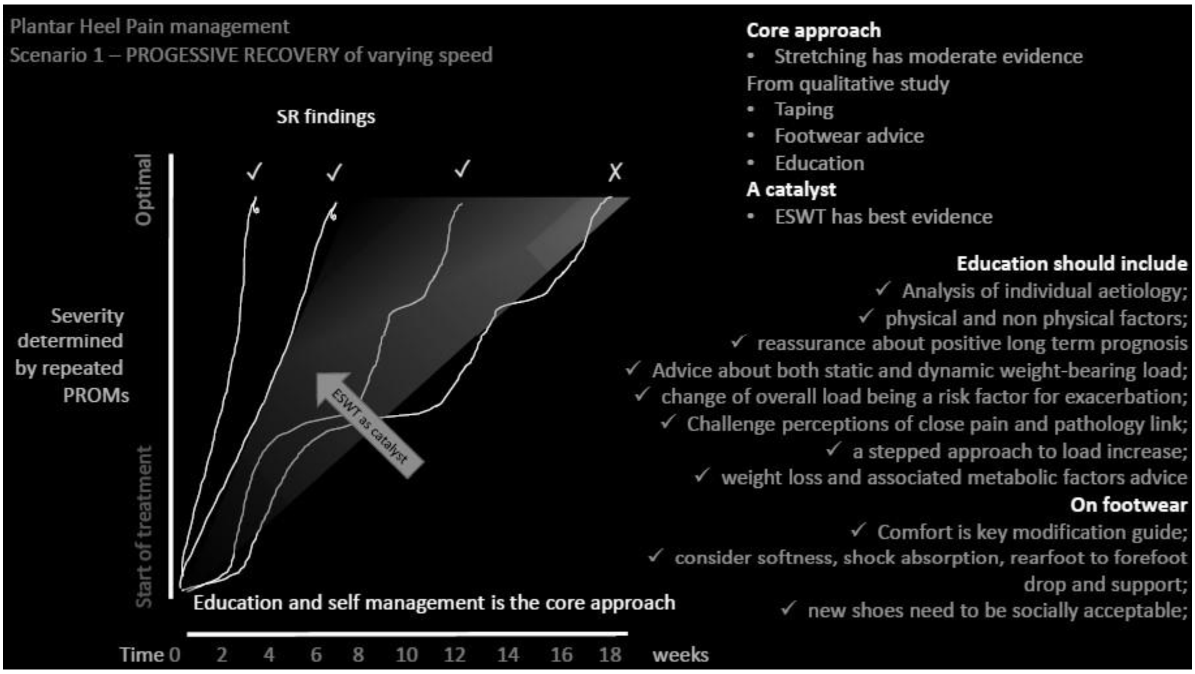

In this scenario, additional interventions are recommended for people who are not recovering.

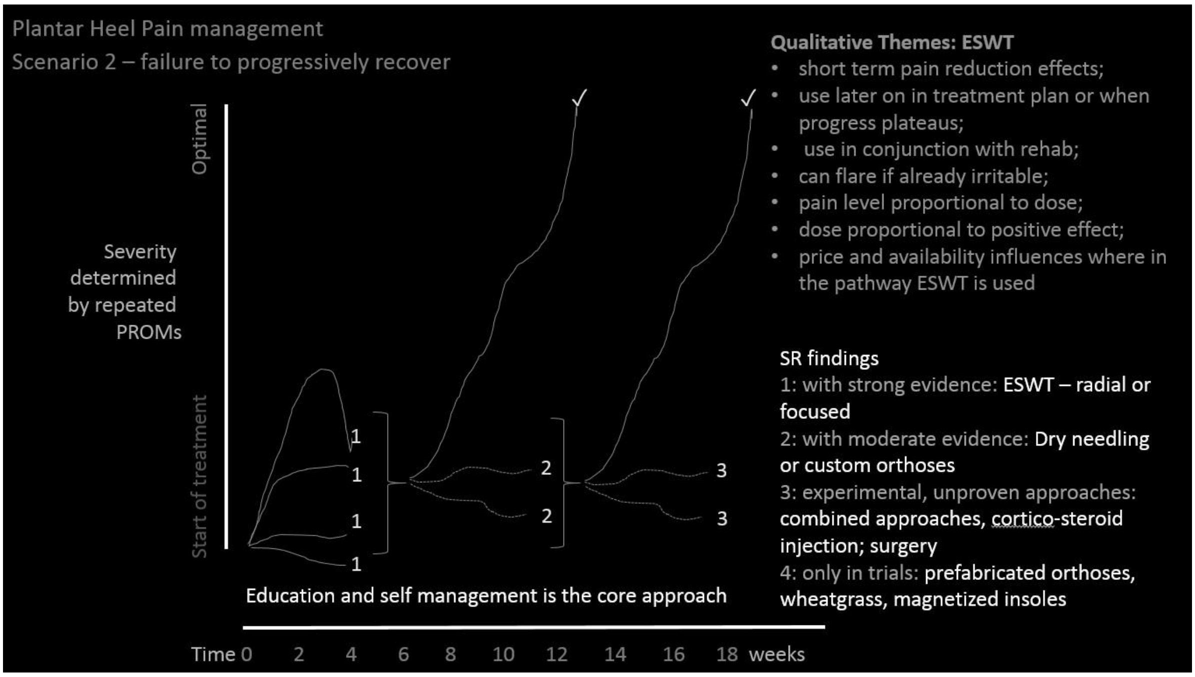

3 strengths of the infographics

3 weaknesses of the infographics

3. Qualitative analysis of expert interviews

3.1.Qualitative analysis of interview data pertaining to management principles

Theme 3: FACTORS UNDERLYING MANAGEMENT		
Adherence	Compliance is a huge issue; patient must buy in to the plan; patient will need to work hard at treatment to get results; up to clinician to get patient buy-in; compliance a key early goal; non-adherence underpins poor results; explaining mechanism of treatment helpful; really good education essential; patients want quick results; pain management principles education; weight loss requirement a major barrier; pain during exercise a barrier; passive treatments better adhered to	Q: a reason why people opt for injection (7)<br>Q: high effort treatments have lower success due to compliance (7)<br>Q: I've got a team of physios and doctors who sort of support, me, and I get them to be eyes and ears and will constantly ask them have they done what they need to do<br>Q: at 2 o'clock in the morning when the baby is crying, she is not so worried about doing her plantar fascia stretches for ten seconds ten times (11)<br>Q: trying weight loss strategies when their foot is painful and they can't do a lot of activities they like to do is really really difficult (12) Q: it's about bite sized chunks in terms of enhancing adherence (12)<br>Q: That's the nut to crack (13)<br>Q: I like to try things that I know are going to reduce their symptoms relatively quickly (3)
Costs	cost and access can be barriers to treatment; may require multiple visits; limited by insurer; more expensive treatments used less as a result; effect on occupation can be a promotor of adherence and engagement with treatment; ESWT cost a barrier; costs mainly low so cost-effectiveness high but profits low;	Q: (commissioners) are trying to find the one cheap intervention, hopefully, that can give you relief (10)<br>Q: if you've invested $20,000 or whatever it might be in the shockwave machine, you need to use that. You need to make that money back (11)<br>q: because although it is an expensive machine it does have NICE guidance and it is approved for private health insurance, so actually you could make your money back very very quick! (12)<br>Q: if you don't have good shoes, it's difficult to give them the right orthoses (14)
Length of symptoms	stage of treatment may indicate prognosis; reactive stage do better; degenerative stage do less well; duration proportional to time to take effect	Q: if you can get plantar fasciopathy at the reactive state, then they respond better to all of the small things (7)<br>Q: people who have had it for a shorter time tend to improve and have better outcomes than people who have had it for a long period of time (11)
Psycho-social Factors	only in occasional cases; salient if there is a perceived benefit to be unwell	Q: if they've had the pain for two years – how much has it (PHP) changed them? Or were they always kind of like this, and it just accentuated it? (13)<br>Q: you can't get anywhere if your foot hurts. And people take their feet for granted – they don't expect them to hurt (14)<br>Q: odd cases, where they want to be in pain, because they want attention or they don't work (7)

3.2.Qualitative analysis pertaining to diagnosis and patient education

Included in main manuscript.

3.3.Qualitative analysis of interview data pertaining to specific interventions

Findings		Illustrative quotes
Theme 4: SPECIFIC INTERVENTIONS		
ESWT		
Usage	short term pain reduction effects; use later on in treatment plan or when progress plateaus; pain vs structural effect; use in conjunction with rehab; can flare if already irritable; increasing use	Q: let's use it as a window to address other issues so when it stops working we are now stronger, more mobile, wearing the right shoes, our training is appropriate (10)<br>Q: there's enough evidence to suggest that patients with heel pain that have shockwave therapy tend to have less pain on review than the patients that don't have shockwave therapy (14)<br>Q: you might be struggling to progress from doing something like a single leg heel raise to a bit more loading, like jumping and hopping, and you give them mild shockwave treatment then suddenly they can jump (9)
Perception of evidence	merit if used at higher dose; highest evidence of interventions; efficacy blurred by low quality studies; better studies show lesser effects; different machines maybe have different effects	Q: its more effective if you give a higher dose but you cant use local analgesics (2)<br>Q: good RCTs tend to show that it's got a pretty limited effect (7)
Intensity	pain level proportional to dose; dose proportional to positive effect; radial gentler and as effective as focussed	Q: find the one spot that is the most painful .. start at down low, at two bar, and then just generally increase it over sessions depending on their own pain and how they are feeling (11)<br>Q: pretty sure there's evidence for both (radial, focussed) (3)
barriers	price and availability influences where in the pathway ESWT is used	Q: shockwave, definitely the price can be a barrier, and again I always wait until at least 6 months after the onset of symptoms to get them to this point (5)
Taping		
General principles of use	effective way into pain; good for compliance and building relationship with patient; useful if standing ++ or sportingly active; short term response; proprioceptive adjunct; improves confidence; good to improve foot function; helps windlass function in pronators; unloads the fascia; adjunct to other treatments; use in acute situation; trackside use; first line treatment; augments tibialis posterior and fat pad function; use to predict orthosis effect	Q: low dye taping, which has three functions: it helps to support the foot position, it takes strain off the fascia and keeps the natural fat pad beneath the heel of the foo (14)<br>Q: if I tape them and their symptoms decrease and then I can say – okay, I think I can replicate what the tape is doing with either shoes or orthoses (13)<br>Q: the greatest issue is that its really a short term treatment. (7)<br>Q: they would be able to tell me almost immediately if it does any good (4)
Specific aspects of use	can leave for up to a week; care with circulation and skin required; low dye taping vs augmented low dye; low allergy tape helpful for longer term use; non-elastic tape better; correct foot position as	Not needed

	applied; benefit that patient can self-apply	
Perception of Evidence	little evidence; good in short term; not sure; interested to know more; lack of trials beyond short term	Q: the RCT we ran showed that's it effective and other RCTs have shown it to be effective as well (7)<br>Q: all we can really say its got short term effectiveness (7)
Orthoses		
Principles	method to break pain cycle; unload the tissue; may shift load elsewhere in kinetic chain; may get immediate pain reduction; orthotics another form of stretching; use if cannot rest the foot from excessive use; relate to amount of standing in ADL; positive tape effects suggest orthoses may be beneficial; costly so wait for lack of response to cheaper interventions	Q: foot was adequately catered for by an off the shelf orthosis, which probably 70% of patients are (14)<br>Q: short term relief while waiting for rehabilitation effects (5)<br>Q: in most cases for me it only a temporary thing until we can get muscle control of the, that movement pattern (1)
Specifics	high arch adds stretch; equinus will require heel raise; overweight requires more support; if severe pain, excessive pronation not an essential indication; costly so wait for lack of response to cheaper interventions; redistribute pressure with close fitting orthoses;	Q: An orthotic with a high arch to put a bit of a stretch on the plantar fascia (8)<br>Q: give them something that would come up and match their arch, provide total contact, try to distribute the forces through the foot completely over all the entire aspect of the foot rather than just in two or three places. (13)
Perception of Evidence	no difference between prefabricated and custom orthoses; evidence and experience match; only evidence is short term relief; same as placebo	Q: based on the work that we have done we find that over the counter insoles work as well as bespoke insoles for the vast majority (12)
Injections		
General Usage	Useful mainly in short term; detrimental in long term; better rehab service leads to less injections; use after stretching; ticket to treatment; local anaesthetic effects can mislead patients; best avoided; last resort; use in more irritable presentation; may be the anaesthetic is the useful element; patient pressure for quick results difficult to resist; may reduce tissue thickness; fits with a hypothetical model with effect being on pain; may be sub-groups of responders	Q: Q: there is a significant amount of inflammation that would benefit from soft tissue injection (12) Q: miserably painful to do – not matter how you try (12)<br>Q: steroid injections, to get a faster improvement, and some patients are very keen on getting a change immediately (4)<br>Q: if not improving … first thing in the morning, which is a good marker – then I would consider injecting it (8)<br>Q: (orthopaedic dept) actually used it less and less after they took part in of the loading study we did (2)
Perception of Evidence	Poor evidence except in short term; better under image guidance	Q: all the evidence regardless of what tendon you're looking at show that it works as a shot term pain alleviator and then the pain comes back again (3)<br>Q: it stands to reason that if you're guiding something right into the fascia then you're going to have more success (6)
Complications	risk of rupture; worse in long term; fat pad atrophy risk; side effects overstated; fluorinated steroids higher risk; choice of	Q: long term they lead to issues, and possibly, my feeling is patients get worse in the long term (3)<br>Q: There are risks with the injection of rupture and fat pad atrophy, so that would be in my mind (14)

	steroid may influence risk	
autologous blood		
Usage	last resort; unlikely to be effective; really bad responses; not used	Q: Interventions that some of my clients get that I am not necessarily involved in or really agree with, are the use of PRP (10)<br>Q: I never use PRP, or suggest PRP for the plantar fascia, and the reason or that is just a personal reason, anecdotally, I've had really bad responses to PRP (3)
Perception of Evidence	better design shows absence of results; poor quality research; new treatment so hard to judge; needs better research	Q: show that unanimously the PRP is effective, that's case series, and the you have RCTs, and there's not many of them, but the RCTs and I know the one in the lower limb, there two on the Achilles, they show that there is no benefit over placebo (4)
Manual therapy		
Usage	soft tissue work to address soft tissue tightness; mobilising sub-talar and ankle joints helpful; variable effects in patients; low priority and rarely used; hard to tell if effects psychological or physiological	Q: I would use that if mobility was a problem (10)<br>Q: tend to do manual therapy a little bit more in the sub acute stage (13)<br>Q: really depends on the patient a little bit and probably their expectations of what they want (9)
Perception of Evidence	unsure about evidence; low quality; maybe has some merit; under-tested	Q: I'm not familiar with any great strong evidence anyway, but that doesn't mean it doesn't work (1)
Night splints		
Usage	rarely used; may help first step pain; does not address cause; useful if first step pain severe or very stiff am; aim is long term stretch; can interrupt sleep, difficult in hot climates	Q: problem with those is that some people can't tolerate them – they keep them awake in bed at night (14)<br>Q: if the person is very very stiff, has a lot of morning stiffness ok why not use it (5)
Perception of Evidence	Unsure; less effective than first line treatments; some short-term benefit;	Q: I know that its shown in the Achilles, in the plantar fascia I think there is limited evidence (5)
Surgery		
Usage	More common and questionable rationale with non-specialist surgeons; better to counsel patient to wait and not opt for early surgery; may compromise foot function; partial or full fascial release in very resistant cases may be useful; risk is high; more common in private sector; see negative effects but may be patient self-selection; may take years to realise effects; suggested benefits not matched by experience; not enough experience to judge	Q: one patient every five years (8)<br>Q: I can't see physiological rationale that it might be helpful (9)<br>Q: keloid scarring and other complications, so it hasn't been favourable (10)<br>Q: patients probably had it done because they were looking for a quicker fix. And surgery offered that to them, but it turned out bad. Now I do know, however, that I only see the ones that went bad (13)<br>Q: I have operated on a very small number myself with poor outcomes, and therefore it is something that I think is a last resort (14)
Perceptions of evidence	good trials underway and needed; novel approaches such as endoscopy may be useful	Q: I think it's very important to compare conservative treatment to surgery and I think that there are no studies (6)

3.4. Qualitative analysis, perceptions rehabilitation and exercise

	Findings	Illustrative quotes
Theme 5: Rehabilitation		
Exercise principles to optimise outcomes	***tensions between:*** *consider local dynamics vs consider whole kinetic chain;*<br>*holistic vs PF focussed approach; increasing vs decreasing load if pain flaring; treat as tendinopathy vs a PF-specific manner progressively load PF vs stretching only*	Q: I might consider (a loading programme) in a sedentary person that's been resistant to a range of other treatments.(14)<br>Q: strengthening exercises which are very similar to Achilles tendinopathy treatment (6)<br>Q: the key is getting that neuromotor control then to work through progressive loading (1)<br>Q: No evidence that I believe that there's any weakness link with calf or anything (2)<br>Q: wouldn't go for strengthening programme you would stick to more stretching? That's correct, yep … I'd like to see more RCTs, preferably larger RCTs that are evaluated before I make up my mind. (7)
Exercise specifics	gradual changes in activity important; total may take 2-3 months vs quick results possible; more than 24 hours of pain aggravation suggest overdose; Facilitate tibialis posterior action; medial arch and dynamic foot posture control important; aim to find ways into pain to facilitate improved rehab and function: pain resolution during ADL not sufficient to represent cure; internal patient locus of control important; may need to consider strength in multiple planes of movement; consider intrinsic foot muscle strength	Q: I want to get their symptoms reducing and then introduce the loading programme (14)<br>Q: apply small dorsiflexion of the hallux, to increase the loading of the fascia … most important part of the treatment (4)
Stretching		
essential	stiffness almost universal; both calf muscle and plantar fascia stiffness common; need to be specific vs address all of it; include in multi-modal approaches; use early and throughout; consider passive and manual and orthosis driven stretching	Q: absolutely hammer plantar fascia stretches. I love plantar fascia stretches. (11)<br>Q: stretching fascia down there, carry on doing throughout (8)<br>Q: the management needs to be three pronged, and unless you do all three your chances of resolution are lessened… typically stretch the Achilles tendon and the plantar fascia… manage the, what we will call "inflammation," for want of a better word…the third element will be to control their foot mechanics. (9)
effectiveness	helps symptoms; may not help load capacity; treats pain vs treats mechanics; maybe less effective than mixed methods; may be less effective than other interventions; fits a model of mechanical - pain - abnormal function; breaks vicious cycle; need better solutions; should work quickly	Q: stretching is kind of band aid effect, so you can use it potentially at the beginning (9)<br>Q: they can feel an immediate response, and there seems to be some adaptation to this stretching, but again I would say this is definitely not the cure for this. (4)
evidence perceptions	gold standard in early 21st century; own trials and influence practice strongly;	Q: the evidence for stretching long term is not good but there is short term evidence for plantar fascia stretching and short term evidence for the calf muscle stretching (7)<br>Q: I've really almost changed some of my practice from doing the trial and hearing the feedback that you get. So I've put a lot of emphasis on plantar fascia stretches (10)

3.5.Qualitative analysis of interview data pertaining to perceptions of the evidence

Theme 6: PERCEPTIONS OF EVIDENCE		
Findings	Illustrative quotes	
Improving Evidence Translation	Short, concise synopses most useful; open access essential; directed at patients and professionals; variety of methods; time not access is the modern barrier; clinical skills in explanation deficient; this kind of study useful; balance generic information synopses with need for different approaches for different patients; live guidelines, social media and websites underused; training champions across the country	Q: should think of other ways of delivering this information, should it be through a video or an interactive thing on the screen where you click through on diagnoses (2)<br>Q: we just need to make it more accessible to the consumer that needs it, so it's for the clinician and the patient (9)<br>q: The main barrier is probably time (11)
Areas to prioritise in future research	combined approaches (ESWT and Ex); best exercise approach; how to individualise (exercise); stratified sub-groups by presentation (Gender, PA level, body mass); enough ESWT, orthoses, CSI research; move from efficacy to effectiveness vs need higher effects sizes in efficacy to move forward; purported detrimental effect of steroid injection needs clarified; weight loss and NSAID RCTs needed	Addressing the posterior kinetic chain ()<br>Q: there are lots of treatments that clinicians would argue black and blue work, yet the evidence suggests that it may work and it may not work (9)<br>q: you may have some evidence for an arm of that, with some evidence for foot orthoses, but there is very little evidence for the combination. Because it is really difficult to manage, for want of a better phrase, the "dose," (12)<br>Q: if you have a decent outcome measure it would make designing the trial a whole heap easier. (12)<br>Q: often there is a poor definition of what you are treating. (14)

4. Qualitative analysis of patient survey

Included in main manuscript.

5. Supplementary systematic review results

5.1. Characteristics of included studies

Table: Characteristics of included studies related to orthoses

**Study**	**Number of participants**	**Outcome measure**	**Interventions**	**% Female**	**Age (mean ± SD)**	**Duration of heel pain (mean ± SD, weeks or months)**	**Follow up period**
Baldassin (2009)	142	FFI: pain, overall	Custom made orthoses (95% EVA) (70)<br><br>Prefabricated orthoses (95% EVA) (72)	73<br><br>78	47.2 (12.4)<br><br>47.5 (11.5)	20.1 (29.2) months<br><br>15.7 (21.6) months	4, 8 weeks
Bishop (2018)	60	VAS: first step pain<br><br>VAS: average 24 hour pain<br><br>Plantar fascia	Control group: sham insole with existing shoes<br><br>Shoe group: (ASICS Nimbus 14, ASICS Corp. Japan)<br><br>Shoe + orthoses: (ASICS	65<br><br>60<br><br>70	44.7 (13.3)<br><br>44.9 (14.5)<br><br>44.5 (13.0)	6.0 (3.1)<br><br>6.1 (3.3)<br><br>6.2 (2.5)	12 weeks

		thickness	Nimbus 14, ASICS Corp. Japan) + 4mm polypropylene orthoses with a EVA rearfoot post				
Landorf (2006)	136	FHSQ: pain, function	Custom made orthoses (46)<br><br>Sham orthoses (46)<br><br>Firm prefabricated orthoses (44)	74<br><br>67<br><br>57	49.2 (12.0)<br><br>48.5 (9.6)<br><br>47.3 (11.6)	12 (2-360 months)<br><br>12 (range 1-240 months)<br>11 (range 2-360 months)	3, 12 months<br>52 weeks
Oliveira (2015)	74	FHSQ – pain, function	Custom made orthoses (ethyl vinyl acetate) (37)<br><br>Sham orthoses (37)	81<br><br>97	48 (10.1)<br><br>53 (10.8)	48 (143.7) weeks 11.0<br><br>48 (171.1) weeks 11.0	45, 90, 145 days<br>20.7 weeks
Vicenzino (2015)	150	NRS: worst pain in the preceding week<br><br>Global Rating of Change scale (GROC)<br><br>LEFS	Firm prefabricated orthoses (51)<br><br>Contoured sandal (49)<br><br>Flip flop sandals (50)	63<br><br>65<br><br>76	50 (13)<br><br>52 (11)<br><br>50 (12)	24 (12, 56) median (IQR) weeks (5.5)<br><br>24 (12, 56) (5.5)<br><br>22 (10, 40) (5.0)	4, 8 and 12 weeks
Winemiller (2003)	101	VAS: first step pain	Magnetised insoles (57)<br><br>Non-magnetised insoles	68<br><br>88	42.0 (9.5)<br><br>40.4 (8.9)	85 (86) months<br><br>120 (170)	8 weeks

			(44)			months	
Wrobel (2015)	77	FFI-R: revised foot function index<br><br>VAS: first step pain	Custom made orthoses (26)<br><br>Sham orthoses (35 durometer, 3mm base of EVA) (26)<br><br>Prefabricated orthoses (45 durometer EVA) (25)	63% overall	49.6 (12.0) overall	5.2 (3.2) months	1, 3 months<br>13 weesk
Xu (2019)	60	VAS: foot comfort	Customised orthoses<br><br>Prefabricated orthoses	50	40.31 (5.21)<br><br>42.52 (6.18)	20.5 (6.4) months	8 weeks

Night splints

Table: Characteristics of included studies related to night splints

Study	Number of participants	Outcome measure	Interventions	% Female	Age (mean ± SD)	Duration of heel pain (mean ± SD, weeks or months)	Follow up period
Wheeler (2018)	40	VAS: average pain; first step pain;<br><br>Foot Function Index – R (total)	Night splint + HEP which included static stretches of the plantar fascia, the calf (selectively involving both gastrocnemius and soleus for different stretches), plus Flexor Hallucis Longus (FHL) and hamstrings, as well as calf and intrinsic foot muscle strengthening and balance training exercises (20)<br><br>HEP which included static stretches of the plantar fascia, the calf (selectively involving both gastrocnemius and soleus for different stretches), plus Flexor Hallucis	75<br><br>65%	53.4 (8.9)<br><br>50.9 (11.7)	25.2 months	3 months<br>13 weeks

			Longus (FHL) and hamstrings, as well as calf and intrinsic foot muscle strengthening and balance training exercises (20)				

ESWT

Table: Characteristics of included studies related to ESWT

**Study**	**Number of participants**	**Outcome measure**	**Interventions**	**% female**	**Age (mean ± SD)**	**Duration of heel pain (mean ± SD, months) unless otherwise stated**	**Follow up period**
Abt (2002)	32	VAS: first step pain	Focused ESWT (17) – 1000 pulses; energy flux density of 0.08mj/mm2<br><br>Sham ESWT (15) – energy absorbing foil that prevented transmission)	64<br><br>40	56.5<br><br>57.4	n/a	48 weeks
Buchbinder (2002)	166	VAS: overall, first step pain<br><br>Maryland Foot Score	Ultrasound guided focused ESWT (81) - 2000 or 2500 pulses x 3 sessions; energy flux density of 0.02 to 0.33mJ/mm^2<br><br>Sham (85) – 100 pulses; energy flux density of 0.02mJ/mm^2	58<br><br>58	52.2 (12.81)<br><br>54.2 (12.05)	36 (8-600) weeks median (range) (8.28 months)<br><br>43 (8-980) median (range) 9.89 months	6 and 12 weeks
Gerdesmeyer (2008)	252	VAS: overall pain and first step pain<br><br>Roles and Maudsley	Radial (125) - 2000 pulses x 3 sessions two weeks apart; energy flux density of 0.16mJ/mm^2<br><br>Placebo (118) – placebo hand piece that	70<br><br>67	52.4 (12.0)<br><br>52.0 (10.5)	25.6 (26.1) months<br><br>24.9 (25.3) months	3 months 13 weeks

			prevented ESWT transmission				
Gerdesmeyer (2016)	106	VAS: overall pain<br><br>Roles and Maudsley	Placebo verum (52) – participants told that they would be treated by real and effective ESWT. 2000 pulses delivered although application pressure was 0.<br><br>Placebo-placebo (53) – participants told that the placebo treatment they would receive would have no effect. 2000 pulses delivered although application pressure was 0.	60<br><br>60	51.0 (10.5)<br><br>49.3 (9.4)	18.4 (19.3) months<br><br>18.2 (21.6) months	6 weeks
Gollwitzer (2007)	40	VAS: first step pain, overall pain<br><br>Roles and Maudsley	Focused (20) – 2000 pulses x 3 sessions in weekly intervals; energy flux density of 0.25mJ/mm^2<br><br>Placebo (20) – Air chambered polyethylene foil that prevented ESWT transmission.	45<br><br>80	53.9 (12.5)<br><br>58.9 (10.9)	11.3 (7.4) months<br><br>12.1 (8.0) months	3 months 13 weeks
Gollwitzer (2015)	250	VAS: first step pain, overall pain	Focused (125) – 2000 pulses x 3 sessions; energy flux density of 0.25mJ/mm^2	67	50 (11.2)	> 6 months	3 months 13 weeks

		Roles and Maudsley	Placebo (121) – Air filled standoff that prevented ESWT transmission	72	47.4 (10.6)	> 6 months	
Hocaoglu (2017)	72	VAS: pain over the previous week<br><br>FFI: overall<br><br>Plantar fascia thickness	Radial (36) - 2000 pulses (3 times a week); energy flux density of 0.16mJ/mm^2<br><br>Ultrasound guided steroid (36) - ultrasound guided 1ml betamethasone sodium plus 0.5 mL of prilocaine	83<br><br>89	50.2 (8.2)<br><br>47.8 (7.9)	8 (6-24) months<br><br>9 (6-18) months	1, 3, 6 months<br>26 weeks
Ibrahim (2016)	50	VAS: worst pain<br><br>Roles and Maudsley	Radial (25) - 2000 pulses x 2 sessions one week apart; energy flux density of 0.16mJ/mm^2<br><br>Placebo (25) – heel clasp that prevented ESWT transmission	72<br><br>56	56.6 (2.71)<br><br>49.1 (2.55)	> 6 months<br><br>> 6 Months	4, 12, 24 weeks and 2 years<br>104 weeks
Kudo (2006)	114	Roles and Maudsley<br><br>VAS: first step pain	Focused (58) - 3,800 pulses; energy flux density of 0.36 mJ/mm^2 + medial calcaneal nerve block, 5 mL of 1% Xylocaine<br><br>Placebo + anaesthesia (56) – a thin foam cushion with ultrasound gel prevented ESWT	68<br><br>58	51.1 (10.6)<br><br>48.8 (9.8)	31.3 (32.5) months<br><br>27.1 (23.5) months	3 months<br>13 weeks

			transmission.				
Lohrer (2010)	39	VAS: overall pain<br><br>FFI: activity, disability	Radial (19) - 2000 pulses x 3 sessions at weekly intervals; energy flux density of 0.17mJ/mm^2<br><br>Focused (20) - 2000 pulses x 3 sessions at weekly intervals; energy flux density of 0.20mJ/mm^2	42<br><br>40	52 (38-68) median<br><br>45 (34-71) median	> 3 months<br><br>> 3 months	3 months<br>13 weeks
Rompe (2003)	45	VAS: first step pain	Focused (22) - 2100 pulses x 3 sessions; 0.16mJ/mm2<br><br>Placebo (23) – sound reflecting pad	45<br><br>56	43 (32-59) mean (range)<br><br>40 (30-61) mean (range)	20 (12-60) months<br><br>18 (12-72) months	6 months and 1 year<br>52 weeks
Rompe (2005)	86	VAS: first step pain<br><br>NRPS: average pain	Focused (41) - 2000 pulses x 3 sessions at weekly intervals; energy flux density of 0.09mJ/mm^2 + 4ml 1% mepivacaine<br><br>Focused (45) - without anaesthesia	65<br><br>53	48 (22-68)<br><br>50 (30-67)	17 (6-36) months<br><br>15 (6-40) months	3, 12 months<br>52 weeks
Rompe (2010)	102	VAS: First step pain<br><br>FFI	Radial (48) - 2000 pulses x 3 sessions at weekly intervals; energy flux density of 0.16mJ/mm^2	63	49.8 (29-68)<br><br>53.1 (27-70)	3.6 (2-6) weeks<br>0.82 months	8 weeks

			Stretching (54) – plantar fascia stretch 3 x day for 8 weeks + additional plantar fascia stretch (10 second hold x 10 repetitions, 3 x day)	66		3.9 (2-6) weeks<br>0.89 months	
Speed (2003)	88	VAS: first step pain, overall pain, night pain	Focused (46) - 1500 pulses x 3 sessions at monthly intervals; energy flux density of 0.12mJ/mm^2<br><br>Placebo (42) – no skin contact	56<br><br>59	51.7 (25-76)<br><br>52.5 (30 – 73)	16.7 (12-312) months<br><br>13.5 (12-312) months	3 months<br>13 weeks

Local injection

Table: Characteristics of studies related to local injections

Study	Number of participants	Outcome measure	Interventions	% female	Age (mean ± SD)	Duration of heel pain (mean ± SD, months)	Follow up period
**Local corticosteroid versus placebo**							
Crawford (1999)	106	VAS, overall pain	Local steroid and local anaesthetic (24) - 1 ml 2% lignocaine + 25mg prednisolone<br><br>Local anaesthetic (20)<br><br>Local steroid and local anaesthetic and tibial nerve block (22)<br><br>Local anaesthetic and tibial nerve block (13)	65 (overall)	59.4 (11.8)<br><br>56.8 (13.0)<br>53.6 (14.2)<br><br>58.8 (12.4)	11.6 (19.4)<br><br>18.9 (25.7)<br><br>14.8 (23.8)<br><br>8.5 (9.9)	1, 3, 6 months<br>26 weeks
[a]Ball (2012)	65	VAS: overall pain<br><br>Plantar fascia thickness	Ultrasound-guided steroid injection 0.5mL (20 mg) of methylprednisolone acetate + 0.5mL 0.9% saline (22)<br><br>Palpation-guided steroid	55<br><br>64	49. 0 (12.9)<br><br>49.1 (10.7)	6 (6-10) months – median (IQR)<br><br>6 (5-11)	6, 12 weeks

			injection (22)<br><br>Ultrasound-guided saline injection, 1mL 0.9% (21)	48	50.1 (10.6)	7 (5-18)	
McMillan (2012)	82	VAS: first step<br><br>FHSQ: pain, function	1 ml 4mg/ml dexamethasone sodium phosphate (+ tibial nerve block) (41)<br><br>1 ml normal saline + tibial nerve block) (40)	54<br><br>42	51.7 (11.9)<br><br>53.6 (9.0)	9.0 (8.0) months (median interquartile range)<br><br>12.0 (11.5) months (median interquartile range)	3 months 13 weeks
**Local steroid versus botulinum toxin**							
Diaz-Llopis (2011)	56	FHSQ: pain, function	Local steroid (28) – 2mL betamethasone 6 mg/mL plus local anaesthetic (0.5 mL of 1% mepivacaine)<br><br>Botulinum toxin A (28) - 100U Botulinum toxin A in 1ml saline. 40U injected into insertion of plantar fascia and 30U at midpoint of plantar fascia.	64<br><br>68	56.3 (14.7)<br><br>51.5 (14.7)	Not reported	1 month 4.34 weeks
**Local steroid (methylprednisolone) versus local steroid (dexamethsone)**							
Ahmed (2013)	60	VAS: first step pain	Local steroid (30) - 1cc 40 mg methylprednisolone	40	48.2 (8.5) overall	14.3 weeks 3.29 months	4, 8 and 12 weeks

			and 1cc of 2% lignocaine<br><br>Local steroid (30) - 1ml of 4mg/ml of dexamethasone phosphate and 1cc of 2% lignocaine	 <br>47		 <br><br>14.2 weeks<br>13.26	
**Local steroid (methylprednisolone) versus Ozone**							
Babaei-Ghazani (2018)	30	VAS: first step	1US guided cc of lidocaine 1% + 2 cc of ozone (O2-O3)<br>US guided 1cc of lidocaine 1% + 1 cc of methylprednisolone (40 mg mixed with 1cc of normal<br>Saline)	90 (overall)l	48.47 (8.8)<br><br><br>44.1 (9.1)	7.8 (6.4)<br><br><br><br>11.9 (14.4)	12 weeks
Bahrami (2019)	50	VAS: pain<br><br>FAAM: (function)	Ozone gas (3mls + 1 ml 2% lidocaine (25)<br><br>Local steroid: 1ml methylprednisolone (40 mg) and 1 ml 2% lidocaine (25)	62<br><br><br>70	47.7 (9.7)<br><br>47.5 (8.7)	9.7 (4.9)<br><br><br>10.2 (7.5)	3 months
**Local steroid versus prefabricated orthoses**							
Whittaker (2019)	103	FHSQ: overall pain;<br>function;<br><br>VAS: first step pain, average	Corticosteroid injection<br><br><br>Prefabricated orthoses	60.0<br><br><br>62.3	44.9 (12.8)<br>42.9 (10.9)	6 (8) (median (IQR)<br><br>6 (8) (median (IQR)	12 weeks

		pain<br><br>EQ-5D: QoL<br><br>SF-36: QoL					
**Local steroid versus strength and stretching versus steroid combined with strength and stretching**							
Johannsen (2019)	90	VAS: pain during function<br><br>FFI: function and pain	Corticosteroid injection (31)<br><br>Strength training (30)<br><br>Corticosteroid injection + strength training (29)	45<br><br>70<br><br>58	45 (10)<br><br>44 (8)<br><br>48 (8)	8 (5)<br><br>11 (9)<br><br>17 (22)	3, 6 months
**Polydeoxyribonucleotide versus placebo**							
Kim (2015)	40	VAS: pain during activity<br><br>Manchester-Oxford Foot Questionnaire (MOXFQ)	Polydeoxyribonucleotide (20) – 1.5 ml of Polydeoxyribonucleotide was injected into the heel weekly for 3 weeks<br><br>Placebo (20) – 1.5ml of saline weekly for 3 weeks.	65<br><br>80	52 (34-68)<br><br>55 (42-710	Pain greater than 6 months	12 weeks
**Polydeoxyribonucleotide versus corticosteroid**							
Lee (2019)	44	VAS: pain<br><br>Manchester-Oxford Foot Questionnaire (MOXFQ)	Polydeoxyribonucleotide (20) – 1.5 ml of Polydeoxyribonucleotide was injected into the heel weekly for 3 weeks<br><br>Corticosteroid – 20mg of triamcinolone + 3mls of 1%	63<br><br>90	56.2 (12.9)<br><br>50.8 (11.5)	> 3 months<br><br>> 3 months	6 weeks and 6 months

			lidocaine at the first visit.<br>3mls of normal saline at weeks 2 and 3.				
**Hyaluronate (high does) versus hyaluronate (low dose) versus hyaluronate (very low dose)**							
Kumai (2017)	168	Roles and Maudsley<br><br>VAS: average pain over a period of several days	Hyaluronate –H (58) - 25mg of HA in 2.5 mL<br><br>Hyaluronate –L (50) - 8mg of HA in 0.8 mL<br><br>Hyaluronate – VL (60) – 0.25mg of HA in 2.5 mL	67<br><br>67<br><br>66	50.4 (20-73)<br><br>52.0 (24-74)<br><br>54.1 (27-74)	220.5 (161-422) days – median (interquartile range) 7.2<br><br>211.0 (118-466) 6.93 months<br><br>246 (137-409) 8.08	5 weeks
**Micronized dHACM versus saline injections**							
Cazzell (2018)	147	VAS<br><br>Foot Function Index–Revised (FFI-R)	Micronized dHACM<br><br>Sodium chloride (0.9%)	53.4<br><br>62.5	48.7 (11.0)<br>53.0 (9.0)	179.3 (101.7) days (5.89)<br>205.8 (124.4) days (6.76 months)	3, 6, 12 months<br>52 weeks
**Ultrasound guided platelet rich plasma versus platelet poor plasma**							
Malahias (2019)	36	VAS: pain function, satisfaction	Platelet rich plasma (18)<br><br>Platelet poor plasma (18)	n/a	n/a	> 6 months	3, 6 months

Pulsed radiofrequency

Table: Characteristics of studies related to pulsed radiofrequency

Study	Number of participants	Outcome measure	Interventions	% female	Age (mean ± SD)	Duration of heel pain (mean ± SD, months)	Follow up period
Wu (2017)	36	VAS: overall pain, first step pain.<br><br>Plantar fascia thickness	Ultrasound guided pulsed radiofrequency of tibial nerve (18) – A radiofrequency probe was inserted into the region of the posterior tibial nerve. PRF stimulation was applied for 120 seconds at 2Hz, with a 30-millisecond pulse width at 42°C.<br><br>Ultrasound guided local anaesthetic injection (18) - 0.5mL of 2% lidocaine was injected around the posterior tibial nerve	60<br><br>45	49.5 (9.9)<br><br>44.7 (13.8)	9.6 (5.2) months<br><br>10.0 (4.9) months	1, 4, 8 and 12 weeks
**Ultrasound guided pulsed radiofrequency of calf muscle versus sham**							
Ye (2015)	100	FHSQ: pain, function.	Ultrasound guided pulsed radiofrequency (PRF) of calf muscle	38	49.0 (13.8)	4.5 (3.1) years<br>54 months	3, 6 months<br>26 weeks

		VAS, first step pain.	(gastrocnemius trigger point) (50) – PRF at 42°C for 5 minutes + 3ml 0.5% levobupivacaine into a gastrocnemius trigger point<br><br>Sham (50) – same procedure as the intervention group but PRF not applied.	38	51.8 (11.3)	5.7 (3.5) years<br>68.4 months	3, 6 months<br>26 weeks

Low dye taping and iontophoresis

Table: Characteristics of studies related to low dye taping and iontophoresis

Study	Number of participants	Outcome measure	Interventions	% female	Age (mean ± SD)	Duration of heel pain (mean ± SD, months)	Follow up period
Osborne (2006)	31	VAS: worst pain	Low dye taping, stretching and 6 treatments of iontophoresis over 2 weeks with Placebo	60	52.2 (10.7)	7.5 (5.0) months	4 weeks

			(0.9% NaCl) (10)<br><br>Low dye taping, stretching and 6 treatments of iontophoresis over 2 weeks with 0.4% Dexamethasone (11)<br><br>Low dye taping, stretching and 6 treatments of iontophoresis over 2 weeks with 5% Acetic acid (10)	 <br>72<br><br><br><br><br><br><br>30	 <br>49.3 (13.3)<br><br><br><br><br><br><br>52.0 (7.7)	 <br>8.1 (7.5) months<br><br><br><br><br><br><br>18.6 (19.2)	

Low dye taping

Table: Characteristics of studies related to low dye taping and iontophoresis

Study	Number of participants	Outcome measure	Interventions	% female	Age (mean ± SD)	Duration of heel pain (mean ± SD, months)	Follow up period
Radford (2006)	92	VAS: first step pain<br><br>FHSQ: overall pain and function	Low dye taping + sham ultrasound<br><br>Sham ultrasound	28<br><br>27	51.3 (13.5)<br><br>49.2 (13.8)	9 months (median)<br><br>10 months (median)	2 weeks

Exercise: Calf stretching

Table: Characteristics of studies related to low dye taping and iontophoresis

Study	Number of participants	Outcome measure	Interventions	% female	Age (mean ± SD)	Duration of heel pain (mean ± SD, months)	Follow up period
Radford (2007)	92	VAS: first step pain<br><br>FHSQ: overall pain and function	Calf stretching + sham ultrasound<br><br>Sham ultrasound	28<br><br>27	50.7 (11.8)<br><br>50.1 (11.0)	13 months (median)<br><br>13 months (median)	2 weeks

Manual therapy and exercise

Table: Characteristics of studies related to manual therapy and exercise

Study	Number of participants	Outcome measure	Interventions	% female	Age (mean ± SD)	Duration of heel pain (mean ± SD, months)	Follow up period
**Foot mobilisation + stretching + ultrasound versus stretching + ultrasound**							
Shashua (2015)	50	NRS: first step pain<br><br>LEFS	Foot mobilisation (ankle, subtalar and midfoot) + stretching (calf and plantar fascia; 2 sets; 30 second holds; 3 x a day + ultrasound (1MHz, 1.5 W/cm^2 50% pulses, 5 minutes. 8 sessions over 4 weeks (25)<br><br>Stretching + ultrasound. 8 sessions over 4 weeks (25)	72<br><br>68	54.16 (13.04)<br><br>48.48 (11.68)	5.28 (4.54) months<br><br>6.54 (5.69) months	6 weeks
**Manual therapy and exercise (stretching) versus iontophoresis, ultrasound and exercise (stretch and strengthening)**							
Cleland (2009)	60	NPRS: overall pain	Soft tissue mobilisation of the	67	49.5 (8.0)	255.4 (190.2) days	4 weeks, 6 months

		LEFS: function	calf and plantar fascia + rearfoot eversion mobilisation + impairments-based manual therapy approach in the region of the hip, knee, ankle, and foot + stretching of the gastrocnemius muscle and/or plantar fascia (30)<br><br>Iontophoresis with dexamethasone + stretching of the gastrocnemius muscle and/or plantar fascia + ultrasound + cryotherapy + intrinsic foot strengthening (30)	73	47.4 (9.3)	8.38 months<br><br>268.0 (237.8) days<br>8.81 months	

Acupuncture

Table: Characteristics of studies related to acupuncture

Study	Number of participants	Outcome measure	Interventions	% female	Age (mean ± SD)	Duration of heel pain (mean ± SD, months)	Follow up period
Cotchett (2014)	84	VAS, first step pain FHSQ, pain FHSQ, function	Trigger point dry needling (Soleus, gastrocnemius, quadratus plantae, abductor halluces, flexor digitorum brevis) (41)	58	54.4 (12.4)	13.4 (14.1) months	6 weeks, 3 months
			Sham dry needling (43)	30	57.8 (12.0)	13.7 (17.3) months	

Wheatgrass

Table: Characteristics of studies related to wheatgrass

Study	Number of participants	Outcome measure	Interventions	% female	Age (mean ± SD)	Duration of heel pain (mean ± SD, months)	Follow up period
Young (2006)	80	VAS: first step pain<br><br>FHSQ	Wheatgrass (42)<br><br>Placebo (38)	62<br><br>55	54.4 (11.2)<br><br>50.0 (12.0)	12 months (median)<br><br>12 months (median)	6, 12 weeks

Cryotherapy ultrasound

Table: Characteristics of studies related to cryotherapy ultrasound

Study	Number of participants	Outcome measure	Interventions	% female	Age (mean ± SD)	Duration of heel pain (mean ± SD, months)	Follow up period
Costantino (2014)	84	VAS: worst pain	Cryotherapy ultrasound (42) – 10 daily treatments, for 20 minutes; -2°C; ultrasound emission of 2.4 Watt/cm^2	42	54.7 (9.9)	Greater than 6 months	3, 12, and 18 months

			Cryotherapy (42)	38	54.3 (8.7)		

Radiation therapy

Table: Characteristics of studies related to radiation therapy

Study	Number of participants	Outcome measure	Interventions	% female	Age (mean ± SD)	Duration of heel pain (mean ± SD, months)	Follow up period
Niewald (2012)	66	VAS	High dose radiation therapy - total dose of 6.0 Gy applied in 6 fractions of 1.0 Gy twice weekly (29)	Not reported	54.4	15.3 months	12 weeks and 1 year
			Low dose radiation therapy - total dose of 0.6 Gy applied in 6 fractions of 1.0 Gy twice weekly (33)		58.0	18.8 months	

Low level laser

Table: Characteristics of studies related to low level laser

Study	Number of participants	Outcome measure	Interventions	% female	Age (mean ± SD)	Duration of heel pain (mean ± SD, months)	Follow up period
**Low level laser versus placebo**							
Macias (2015)	69	VAS: first step pain<br><br>Plantar fascia thickness<br><br>FFI: pain, disability, activity limitations.	Low level laser (37) - infrared wavelength of 904 nm; 17mW of output; 3 x week for 6 weeks<br><br>Sham laser (32)	61% overall	56.7 (31-75)	12.3 (11.0) months<br><br>12.2 (12.4) months	1, 2, 3, 6, and 8 weeks
**High level laser versus low level laser**							
Ordahan (2018)	75	VAS: overall<br><br>FAOS: ADL	LLLT (904 nm) were performed three times per week, over a period of 3 weeks. Each treatment combined with silicone insole and stretching exercises.<br><br>HILT (1064 nm) were performed three times per week, over a	73%<br><br>73%	48.6 (10.8)<br><br>48.7 (11.4)	8.0 (1.6)<br><br>8.0 (1.5)	3 weeks

			period of 3 weeks. Each treatment combined with silicone insole and stretching exercises.				

Electrolysis

Table: Characteristics of studies related to electrolysis

Study	Number of participants	Outcome measure	Interventions	% female	Age (mean ± SD)	Duration of heel pain (mean ± SD, months)	Follow up period
**Electrolysis versus**							
Fernandez-Rodriguez (2018)	73	VAS: overall pain<br><br>Plantar fascia thickness<br><br>Foot and Ankle Ability Measure	Ultrasound-guided percutaneous needle electrolysis of the fascia (39)<br><br>Placebo puncture (34)	23<br><br>19	45.1 (11.4)<br><br>46.6 (11.1)	Heel pain for at least 3 months	12, 24 weeks

5.2.Quality analysis scores

Author (year)	Eligibility criteria	Randomisation	Allocation concealment	Baseline comparability	Participant blinding	Clinician blinding	Assessor Blinding	Attrition	Intention-to-treat	Between group comparisons	Point estimate and variability	Total score	Risk of Bias score
**Radial ESWT**													
Gerdesmeyer 2008	1	1	1	1	1	0	1	1	1	1	1	9	0
Gerdesmeyer 2016	1	1	1	1	1	0	1	1	0	1	1	8	1
Hocaoglu 2017	1	1	1	1	0	0	1	1	1	1	1	8	0
Ibrahim 2016	1	1	1	1	1	0	1	1	1	1	1	9	0
Lohrer 2010	1	1	0	1	1	0	1	1	1	1	1	8	1
Rompe 2010	1	1	1	1	0	0	1	1	1	1	1	8	0
**Focused ESWT**													
Abt 2002	1	1	1	1	1	0	1	1	1	1	1	9	0
Buchbinder 2002	1	1	1	1	1	0	1	1	1	1	1	9	0
Gollwitzer 2007	1	1	1	1	1	0	1	1	1	1	1	9	0
Gollwitzer 2015	1	1	1	1	1	0	1	1	1	1	1	9	0
Kudo 2006	1	1	1	1	1	0	1	1	1	1	1	9	0
Lohrer 2010	1	1	0	1	1	0	1	1	1	1	1	8	1
Rompe 2003	1	1	1	1	1	0	1	1	0	1	1	8	1
Rompe 2005	1	1	1	1	0	0	1	1	1	1	1	8	0
Speed 2003	1	1	0	1	1	0	1	1	1	1	1	8	1
**Corticosteroid injections**													
Ahmed 2013	0	1	0	1	1	0	1	1	1	1	1	8	1
Babaei-Ghazani 2018	1	1	1	1	1	1	1	1	1	1	1	10	0
Bahrami 2019	1	1	1	1	1	1	1	1	0	1	1	9	1
Ball 2013	1	1	1	1	1	0	1	1	0	1	1	8	1

Crawford 1999	1	1	1	1	1	0	1	1	0	1	1	8	1
Diaz Llopis 2013	1	1	0	1	1	0	1	1	1	1	1	8	1
Hocaoglu 2017	1	1	1	1	0	0	1	1	1	1	1	8	0
Johannsen 2019	1	1	1	1	0	0	1	1	1	1	1	8	0
Lee 2019	1	1	1	1	1	1	1	1	1	1	1	10	0
McMillan 2012	1	1	1	1	1	1	1	1	1	1	1	10	0
Whittaker 2019	1	1	1	1	0	0	1	1	1	1	1	8	0
**Ozone**													
Babaei-Ghazani 2018	1	1	1	1	1	1	1	1	1	1	1	10	0
Bahrami 2019	1	1	1	1	1	1	1	1	0	1	1	9	1
**Orthoses**													
Baldassin 2009	1	1	1	1	1	0	1	0	1	1	1	8	1
Bishop 2018	1	1	1	1	1	0	1	1	1	1	1	9	0
Landorf 2006	1	1	1	1	1	0	1	1	1	1	1	9	0
Oliveira 2015	1	1	1	1	1	0	0	1	1	1	1	8	1
Wrobel 2015	1	1	1	1	1	0	1	1	0	1	1	8	1
Vicenzino 2015	1	1	1	1	0	0	1	1	1	1	1	8	0
Xu 2019	1	1	1	1	0	0	1	1	1	1	1	8	0
Whittaker 2019	1	1	1	1	0	0	1	1	1	1	1	8	0
**Contoured sandals**													
Vicenzino 2015	1	1	1	1	0	0	1	1	1	1	1	8	0
**Manual therapy**													
Cleland 2009	1	1	1	1	0	0	1	1	1	1	1	8	0
Shashua 2015	1	1	1	1	0	0	1	1	1	1	1	8	0
**Taping and iontophoresis**													
Osborne 2006	1	1	1	0	1	0	1	1	1	1	1	8	1
**Taping**													
Radford 2006	1	1	1	1	1	0	1	1	1	1	1	9	1
**Trigger point dry needling**													
Cotchett 2014	1	1	1	1	1	0	1	1	1	1	1	8	0

**Cryotherapy**													
Costantino 2014	1	1	0	1	1	0	1	1	1	1	1	8	1
**Laser therapy**													
Macias 2015	1	1	1	0	1	0	1	1	1	1	1	8	1
Ordahan 2018	1	1	1	1	1	0	1	1	0	1	1	8	1
**Electrolysis**													
Fernandez-Rodriguez 2018	1	1	1	1	1	0	1	1	0	1	1	8	1
**Polydeoxyribonucleotide**													
Kim 2015	1	1	1	0	1	1	1	1	1	1	1	9	1
Lee 2019	1	1	1	1	1	1	1	1	1	1	1	10	0
**Botulinum toxin**													
Diaz Llopis 2013	1	1	0	1	1	0	1	1	1	1	1	8	1
**Platelet rich plasma**													
Malahias 2019	1	1	1	0	1	1	1	1	1	1	1	0	1
**Hyaluronate**													
Kumai 2017	1	1	1	1	1	0	1	1	1	1	1	9	0
**Radiation therapy**													
Niewald 2012	1	1	1	1	1	0	0	1	1	1	1	8	1
**Night splints**													
Wheeler 2018	1	1	1	1	0	0	1	1	1	1	1	8	0
**Magnetised insoles**													
Winemiller 2003	1	1	1	1	1	1	1	1	1	1	1	10	0
**Pulsed radiofrequency**													
Wu 2017	1	1	0	1	0	0	1	1	1	1	1	8	1
Ye 2015	1	1	1	1	1	0	1	1	1	1	1	9	0
**Wheatgrass**													
Young 2006	1	1	1	1	1	1	1	1	1	1	1	10	0
**Amniotic membrane**													
Cazzell 2018	1	1	1	1	1	0	1	1	1	1	1	9	1
**Calf stretching**													

Radford 2007	1	1	1	1	1	0	1	1	1	1	1	9	1
**Plantar fascia stretching**													
Rompe 2010	1	1	1	1	0	0	1	1	1	1	1	8	0
**Strengthening**													
Johannsen 2019	1	1	1	1	0	0	1	1	1	1	1	8	0

5.3.Short term results for pain and function

Table: Short term effectiveness of orthoses for pain associated with plantar heel pain

Study	Population	Outcome measure	Interventions	Outcomes	Statistical analysis OR (95% CI) or SMD (95% CI) unless otherwise stated	Favours
**Custom orthoses versus sham orthoses**						
Bishop (2018)	60	VAS: first step pain	Custom made orthoses (20)<br><br>Sham orthoses (20)	22.4 (21.9)<br><br>40.2 (31.8)	-0.64 (-1.28, -0.00)	N/S
Bishop (2018)	60	Average 24 hour pain	Custom made orthoses (20)<br><br>Sham orthoses (20)	15.3 (13.9)<br><br>38.3 (26.7)	-1.06 (-1.73, -0.39)	Custom orthoses
Landorf (2006)	136	FHSQ: pain	Custom made orthoses (46)<br><br>Sham orthoses (46)	-71.80 (20.6)<br><br>-63.40 (21.5)	-0.40 (-0.81, 0.02)	NS
Landorf (2006)	136	FHSQ: function	Custom made orthoses (46)<br><br>Sham orthoses (46)	-84.1 (19.9)<br><br>-79.7 (22.3)	-0.21 (-0.62, 0.20)	NS
Oliveira (2015)	74	FHSQ: pain	Custom made orthoses (37)<br><br>Sham orthoses (37)	-62.0 (27.4)<br><br>-59.8 (27.4)	-0.08 (-0.54, 0.38)	NS
Oliveira (2015)	74	FHSQ: function	Custom made orthoses (37)	-81.3 (18.2)	-0.36 (-0.82, 0.10)	NS

			Sham orthoses (37)	-73.3 (25.5)		
Wrobel (2015)	77	FFI_R: pain	Custom made orthoses (25)<br><br>Sham orthoses (23)	22.4 (9.3)<br><br>23.5 (8.6)	-0.12 (-0.69, 0.45)	NS
Wrobel (2015)	77	VAS: first step pain	Custom made orthoses (25)<br><br>Sham orthoses (23)	2.6 (2.4)<br><br>2.7 (2.1)	-0.04 (-0.6, 0.52)	NS
Wrobel (2015)	77	FFI-R:	Custom made orthoses (25)<br><br>Sham orthoses (23)	57.20 (?)<br><br>62.4 (?)	-0.02 (-0.56, 0.53)	NS
**Custom orthoses versus prefabricated orthoses**						
Baldassin (2009)	142	FFI: pain	Custom made orthoses (70)<br><br>Prefabricated orthoses (72)	31.9 (26.0)<br><br>34.2 (27.6)	-0.09 (-0.47, 0.30)	NS
Baldassin (2009)	142	FFI: total	Custom made orthoses (70)<br><br>Prefabricated orthoses (72)	22.9 (21.3)<br><br>27.9 (24.6)	-0.22 (-0.55, 0.11)	N/S
Landorf (2006)	136	FHSQ: pain	Custom made orthoses (46)<br><br>Prefabricated orthoses (44)	-71.8 (20.6)<br><br>-71.40 (23.2)	-0.02 (-0.43, 0.40)	NS
Landorf (2006)	136	FHSQ: function	Custom made orthoses (46)<br><br>Prefabricated orthoses (44)	-84.1 (19.9)<br><br>-81.8 (22.8)	-0.11 (-0.52, 0.31)	NS
Wrobel (2015)	77	FFI: pain	Custom made orthoses (25)	22.4 (9.3)	-0.07 (-0.65, 0.51)	NS

			Prefabricated orthoses (21)	23.0 (7.68)		
Wrobel (2015)	77	VAS: first step	Custom made orthoses (25)<br><br>Prefabricated orthoses (21)	2.6 (2.4)<br><br>2.5 (2.1)	-0.01 (-0.57, 0.59)	NS
Wrobel (2015)	77	FFI: function	Custom made orthoses (25)<br><br>Prefabricated orthoses (21)	-24.9 (12.2)<br><br>-24.7 (9.5)	-0.02 (-0.57, 0.53)	NS
Xu (2019)	60	VAS: foot comfort	Customised orthoses (30)<br><br>Prefabricated orthoses (30)	3.12 (0.51)<br><br>5.25 (1.22)	-2.25 (-2.90, -1.59)	Favours custom orthoses
**Prefabricated orthoses versus contoured sandals versus flip flops**						
Vicenzino (2015)	150	NRS (0-10)	Prefabricated orthoses (51)<br><br>Contoured sandal (49)	2.0 (0.7)<br><br>3.0 (0.6)	-1.52 (-1.97, -1.07)	Prefabricated orthoses
Vicenzino (2015)	150	NRS (0-10)	Prefabricated orthoses (51)<br><br>Flip flops (50)	2.0 (0.7)<br><br>3.0 (0.8)	-1.32 (-1.75, -0.89)	Prefabricated orthoses
Vicenzino (2015)	150	GROC: Improved > quite a bit better	Prefabricated orthoses (51)<br><br>Contoured sandal (49)	27/51<br><br>27/49	OR: 0.92 (0.42, 2.01)	NS
Vicenzino (2015)	150	GROC: Improved >	Contoured sandal (49)<br><br>Flip flops (50)	27 (49)<br><br>16/50	OR: 2.61 (1.15, 5.91)	Contoured sandal

		quite a bit better				
Vicenzino (2015)	150	GROC: Improved > quite a bit better	Prefabricated orthoses (51)<br><br>Flip flops (50)	27/51<br><br>16/50	OR: 0.42 (0.19, 0.94)	Prefabricated orthoses
Vicenzino (2015)	150	LEFS change category at week twelve >9	Prefabricated orthoses (51)<br><br>Contoured sandal (49)	24/51<br><br>25/49	OR: 0.85 (0.39, 1.87)	NS
Vicenzino (2015)	150	LEFS change category at week twelve >9	Prefabricated orthoses (51)<br><br>Flip flops (50)	24/51<br><br>16/50	OR: 1.89 (0.84, 4.24)	NS
Vicenzino (2015)	150	LEFS change category at week twelve >9	Contoured sandal (49)<br><br>Flip flops (50)	25/49<br><br>16/50	OR: 2.21 (0.98, 5.01)	NS
**Magnetised insoles**						
Winemiller (2003)	101	VAS: first step pain	Magnetised insoles (57)<br><br>Non-magnetised insoles (44)	3.9 (2.6)<br><br>3.9 (2.6)	0.00 (-0.39, 0.39)	N/S

Night splints

Table: Short term effectiveness of nightsplints for **pain and function** associated with plantar heel pain

Study	Population	Outcome measure	Interventions	Outcomes Mean (SD) or Success n/N	Statistical analysis OR (95% CI) or SMD (95% CI) unless otherwise stated	Favours
**Night splint and a home exercise program versus home exercise**						
Wheeler (2018)	40	VAS: Average pain	Night splint + home exercise program (20)<br><br>Home exercise program (20)	5.3 (2.6)<br><br>5.6 (2.9)	-0.11 (-0.73, 0.51)	N/S
		VAS: first step pain	Night splint + home exercise program (20)<br><br>Home exercise program (20)	5.2 (2.7)<br><br>5.8 (3.4)	-0.19 (-0.81, 0.43)	N/S
		FFI-R (total)	Night splint + home exercise program (20)<br><br>Home exercise program (20)	77.6 (25.4)<br><br>77.0 (27.4)	0.02 (-0.60, 0.64)	N/S

ESWT

Table: Short term effectiveness of ESWT for **pain** associated with plantar heel pain

Study	Population	Outcome measure	Interventions	Outcomes Mean (SD) or Success n/N	Statistical analysis OR (95% CI) or SMD (95% CI) unless otherwise stated	Favours
**Radial shockwave versus placebo**						
Gerdesmeyer (2008)	252	VAS: >60% improvement in VAS from baseline for at least 2 of 3 VAS measurements	Radial (125)<br><br>Placebo (118)	75/125<br><br>49/118	OR: 2.11 (1.27, 3.52)	Radial
Gerdesmeyer (2008)	252	VAS: >60% improvement in first step pain	Radial (125)<br><br>Placebo (118)	76 (125)<br><br>57 (118)	OR: 1.66 (1.00, 2.76)	NS
Ibrahim (2010)	50	VAS: >60% improvement in VAS from baseline	Radial (25)<br><br>Placebo (25)	23/25<br><br>1/25	OR: 276.0 (23.4, 3255.3)	Radial
Ibrahim (2010)	50	VAS: overall pain	Radial (25)<br><br>Placebo (25)	-7.44 (2.4)<br><br>-1.2 (1.47)	-3.04 (-3.87, -2.21)	Radial

**Radial versus plantar fascial stretching**						
Rompe (2010)	102	FFI: First step pain (mean change from baseline)	Radial (48)<br><br>Stretching (54)	-1.8 (2.0)<br><br>- 4.5 (2.4)	1.21 (0.78, 1.63)	Stretching
**Radial versus ultrasound guided local steroid**						
Hocaoglu (2017)	72	VAS: pain over the previous week	Radial (36)<br><br>Ultrasound guided steroid (36)	35.0 (12.5)<br><br>40.0 (17.5)	0.33 (-0.14, 0.79)	N/S
Hocaoglu (2017)	72	FFI: overall	Radial (36)<br><br>Ultrasound guided steroid (36)	67.0 (29.7)<br><br>57.0 (19.1)	-0.40 (-0.86, 0.07)	N/S
**Radial versus focused**						
Lohrer (2010)	39	VAS: overall pain	Radial (19)<br><br>Focused (20)	24.9 (24.5)<br><br>25.9 (18.0)	-0.05 (-0.67, 0.58)	N/S
Lohrer (2010)	39	FFI: disability	Radial (19)<br><br>Focused (20)	19.9 (19.7)<br><br>24.9 (18.9)	-0.25 (-0.88, 0.38)	N/S
Lohrer (2010)	39	FFI: activity	Radial (19)<br><br>Focused (20)	3.0 (5.1)<br><br>6.3 (3.7)	-0.73 (-1.38, -0.08)	Radial
**Focused shockwave therapy versus placebo**						
Gollwitzer (2007)	40	VAS: >60% improvement in overall VAS	Focused (20)<br><br>Placebo (20)	11/20<br><br>8/20	OR: 1.83 (0.52, 6.43)	NS

		from baseline for at least 2 of 3 VAS measurements				
Gollwitzer (2007)	40	VAS: >60% improvement in first step VAS from baseline	Focused (20)<br><br>Placebo (20)	11/20<br><br>6/20	OR: 2.85 (0.78, 10.47)	NS
Gollwitzer (2015)	250	VAS: >60% improvement in overall VAS from baseline for at least 2 of 3 VAS measurements	Focused (125)<br><br>Placebo (121)	68/125<br><br>45/121	OR: 2.01 (1.21, 3.35)	Focused
Gollwitzer (2015)	250	VAS: >60% improvement in first step VAS from baseline	Focused (125)<br><br>Placebo (121)	63/125<br><br>44/121	OR: 1.78 (1.07, 2.96)	Focused
Gollwitzer (2015)	250	Composite score: Overall pain	Focused (125)<br><br>Placebo (121)	-54.5 (38.9)<br><br>-40.3 (40.0)	-0.36 (-0.61, -0.11)	Focused
Speed (2003)	88	VAS: >50% improvement in overall VAS from baseline	Focused (46)<br><br>Placebo (42)	17/46<br><br>10/42	OR: 1.88 (0.74, 4.75)	NS
Speed (2003)	88	VAS: >50% improvement in first step VAS from	Focused (46)<br><br>Placebo (42)	19/46<br><br>15/42	OR: 1.27 (0.54, 3.00)	NS

		baseline				
Speed (2003)	88	VAS: overall pain	Focused (46)<br><br>Placebo (42)	-32.2 (62.0)<br><br>-22.9 (41.8)	-0.17 (-0.59, 0.25)	N/S
**Focused + local anaesthesia versus placebo and local anaesthesia**						
Kudo (2006)	114	Roles and Maudsley score of 1 or 2	Focused + anaesthesia (58)<br><br>Placebo + anaesthesia (56)	23/58<br><br>16/56	OR: 0.61 (0.28, 1.33)	N/S
Kudo (2006)	114	VAS: >60% improvement in first step VAS from baseline	Focused + anaesthesia (58)<br><br>Placebo + anaesthesia (56)	25/58<br><br>12/56	OR: 2.78 [1.22, 6.33]	Focused ESWT
Kudo (2006)	114	VAS: first step pain	Focused + anaesthesia (58)<br><br>Placebo + anaesthesia (56)	3.9 (3.2)<br><br>5.3 (2.7)	-0.47 (-0.84, 0.10)	N/S
**Focused + local anaesthesia versus focused**						
Rompe (2005)	86	NPRS: >50% improvement in first step pain from baseline	Focused + anaesthesia (41)<br><br>Focused (45)	12/41<br><br>30/45	OR: 4.83 (1.94, 12.06)	Focused without anaesthesia
Rompe (2005)	86	NPRS: first step pain	Focused + anaesthesia (41)<br><br>Focused (45)	4.1 (1.5)<br><br>2.2 (2.0)	-1.06 [-1.51, -0.61]	Focused without anaesthesia
**ESWT blinded placebo versus ESWT unblinded placebo**						

Gerdesmeyer (2016)	106	VAS: overall pain	Placebo verum (52)<br><br>Placebo-placebo (53)	5.5 (2.2)<br><br>6.3 (2.0)	-0.37 (-0.76, 0.01)	N/S
Gerdesmeyer (2016)	106	Roles and Maudsley score on a 4 point ordinal scale.	Placebo verum (52)<br><br>Placebo-placebo (53)	3.3 (0.6)<br><br>3.7 (0.4)	-0.72 (-1.11, -0.32)	N/S
**ESWT versus ESWT (different intensities)**						
Buchbinder (2002)	166	VAS: first step pain	Ultrasound guided ESWT (81)<br><br>Sham (85)	23.7 (40.7)<br><br>23.5 (42.2)	0.00 (-0.30, 0.31)	N/S
Buchbinder (2002)	166	VAS: overall pain	Ultrasound guided ESWT (81)<br><br>Sham (85)	26.3 (34.8)<br><br>25.7 (34.9)	0.02 (-0.29, 0.32)	N/S
Buchbinder (2002)	166	Maryland Foot Score	Ultrasound guided ESWT (81)<br><br>Sham (85)	-15.0 (20.6)<br><br>-13.9 (20.5)	-0.05 (-0.36, 0.25)	N/S

Local injection

Table: Short term effectiveness of local injections for pain and function associated with plantar heel pain

Study	Participants	Outcome measure	Interventions	Outcomes	Statistical analysis OR (95% CI) or SMD (95% CI) unless otherwise stated	Favours
**Local corticosteroid versus placebo**						
Crawford (1999)	106	VAS: overall pain	Local steroid and local anaesthetic (24)<br><br>Local anesthetic (20)	3.6 (2.8)<br><br>3.7 (3.3)	-0.03 (-0.63, 0.56)	N/S
Crawford (1999a)	106	VAS: overall pain	Local steroid and local anaesthetic + tibial nerve block (22)<br><br>Local anaesthetic + tibial nerve block (13)	3.4 (2.7)<br><br>3.1 (2.7)	0.11 (-0.58, 0.79)	N/S
Ball (2012)	65	VAS: overall pain	Ultrasound-guided steroid injection 0.5mL (20 mg) of methylprednisolone acetate + 0.5mL 0.9% saline (16)	28.29 (24.84) (n=37) [steroid groups combined]	-0.90 (-1.49, -0.31)	Local steroid

			Palpation-guided steroid injection (21)<br><br>Ultrasound-guided saline injection, 1ml 0.9% (18)	 <br><br>53.8 (33.8) (18)		
McMillan (2012)	82	FHSQ: pain	Local steroid + local anaesthetic (tibial nerve block) (41)<br><br>Placebo and local anaesthetic (tibial nerve block) (40)	-65.4 (27.7)<br><br>-59.7 (25.4)	-0.21 [-0.65, 0.22]	N/S
McMillan (2012)	82	VAS: first step pain	Local steroid + local anaesthetic (tibial nerve block) (41)<br><br>Placebo and local anaesthetic (tibial nerve block) (40)	0 (27.6)<br><br>7.34 (27.6)	-0.26 [-0.70, 0.17]	N/S
McMillan (2012)	82	FHSQ: function	Local steroid + local anaesthetic (tibial nerve block) (41)<br><br>Placebo and local anaesthetic (tibial nerve block) (40)	0 (18.2)<br><br>-4.1 (18.2)	0.22 [-0.21, 0.66]	N/S
**Local steroid versus botulinum toxin**						
Diaz-Llopis (2012)	56	FHSQ: pain	Local steroid (28)<br><br>Botulinum toxin (28)	-53.73 (31.18)<br>-63.3	-0.35 (-0.88, 0.18)	N/S

				(21.9)		
Diaz-Llopis (2012)	56	FHSQ: function	Local steroid (28)<br><br>Botulinum toxin (28)	-63.6 (24.1)<br>-70.9 (26.4)	-0.28 (-0.81, 0.24)	N/S
**Local steroid (methylprednisolone) versus local steroid (dexamethsone)**						
Ahmed (2013)	60	VAS: first step pain	Local steroid (methylprednisolone) (30)<br><br>Local steroid (dexamethsone) (30)	5.3 (16.3)<br><br>19.5 (19.1)	-0.79 (-1.31, -0.26)	Methylprednisolone
**Local steroid (celestone chondrose) versus prefabricated orthoses (Formthotics)**						
Whittaker (2019)	103	FHSQ pain	Corticosteroid injection<br><br>Prefabricated orthoses	64.8 (26)<br><br>73.4 (20.9)	-0.36 (-0.75, 0.03)	N/S
		VAS first step pain	Corticosteroid injection<br><br>Prefabricated orthoses	35.3 (30.8)<br><br>19.8 (20)	0.60 (0.20, 0.99)	Favours orthoses
		FHSQ function	Corticosteroid injection<br><br>Prefabricated orthoses	83.9 (20.4)<br><br>87.9 (14.7)	-0.22 (-0.61, 0.16)	N/S
		SF36	Corticosteroid injection<br><br>Prefabricated orthoses	48.5 (6.6)<br><br>49.5 (6.2)	-0.16 (-0.54, 0.23)	N/S
**Local steroid versus strength and stretching versus steroid combined with strength and stretching**						

Johannsen (2019)	90	VAS	Corticosteroid injection (31)<br><br>Strength training (30)<br><br>Corticosteroid injection + strength training (29)	Not available		
	90	FFI	Corticosteroid injection (31)<br><br>Strength training (30)<br><br>Corticosteroid injection + strength training (29)	Data is in a composite form		
**Local steroid versus an ozone injection**						
Babaei-Ghazani (2018)	30	VAS: first step	1US guided cc of lidocaine 1% + 2 cc of ozone (O2-O3) (15)<br><br>US guided 1cc of lidocaine 1% + 1 cc of methylprednisolone (40 mg mixed with 1cc of normal saline) (15)	24.66 (11.8)<br><br>25.3 (26.4)	-0.03 (-0.75, 0.69)	N/S
Babaei-Ghazani (2018)	30	FAAM (daily foot and ankle ability)	1US guided cc of lidocaine 1% + 2 cc of ozone (O2-O3) (15)<br><br>US guided 1cc of lidocaine 1% + 1 cc of methylprednisolone (40	90.70 (9.4)<br><br>78.3 (18.4)	0.82 (0.07, 1.57)	Methylprednisolone

			mg mixed with 1cc of normal saline) (15)			
		FAAM (sports)	1US guided cc of lidocaine 1% + 2 cc of ozone (O2-O3) (15)<br><br>US guided 1cc of lidocaine 1% + 1 cc of methylprednisolone (40 mg mixed with 1cc of normal saline) (15)	81.2 (13.5)<br><br>71.2 (23.3)	0.51 (-0.22, 1.24)	N/S
Bahrami (2019)	50	VAS: pain	Ozone gas (3mls + 1 ml 2% lidocaine (25)<br><br>Local steroid: 1ml methylprednisolone (40 mg) and 1 ml 2% lidocaine (25)	3.1 (1.5)<br><br>3.0 (1.1)	-0.08 (-0.67, 0.52)	N/S
		FAAM	Ozone gas (3mls + 1 ml 2% lidocaine (25)<br><br>Local steroid: 1ml methylprednisolone (40 mg) and 1 ml 2% lidocaine (25)	45.0 (11.7)<br><br>43.0 (10.1)	-0.18 (-0.77, -0.41)	N/S
**Polydeoxyribonucleotide versus placebo**						
Kim (2015)	40	VAS: pain during activity	Polydeoxyribonucleotide (20)	3.7 (2.3)	-1.08 (-1.75, -	Polydeoxyribonucleotide

			Placebo (20)	6.3 (2.4)	0.42)	
Kim (2015)	40	Manchester-Oxford Foot Questionnaire (MOXFQ)	Polydeoxyribonucleotide (20)<br><br>Placebo (20)	27.0 (11.0)<br><br>38.0 (12.0)	-0.94 (-1.59, -0.28)	Polydeoxyribonucleotide
**Polydeoxyribonucleotide versus corticosteroid**						
Lee (2019)	44	VAS: pain	Polydeoxyribonucleotide (22) – 1.5 ml of Polydeoxyribonucleotide was injected into the heel weekly for 3 weeks<br><br>Corticosteroid – 20mg of triamcinolone + 3mls of 1% lidocaine at the first visit. 3mls of normal saline at weeks 2 and 3 (22)	4.2 (1.7)<br><br>2.6 (2.1)	0.82 (0.20, 1.44)	
		Manchester-Oxford Foot Questionnaire (MOXFQ)	Polydeoxyribonucleotide (22) – 1.5 ml of Polydeoxyribonucleotide was injected into the heel weekly for 3 weeks<br><br>Corticosteroid – 20mg of triamcinolone + 3mls of 1% lidocaine at the first visit. 3mls of normal saline at weeks 2 and 3. (22)	35 (12.6)<br><br>23.3 (15.6)	0.81 (0.19, 1.43)	
**Hyaluronate (high dose) versus hyaluronate (low dose) versus hyaluronate (very low dose)**						
Kumai (2017)	168	VAS: average pain over a	Hyaluronate –high and low (108)	3.6 (2.2)	-0.26 (-0.58, 0.06)	N/S

		period of several days	Hyaluronate – VL (60)	4.2 (2.2)		
Kumai (2017)	168	Roles and Maudsley	Hyaluronate –H (58)<br><br>Hyaluronate – VL (60)	13/58<br><br>9/60	OR: 1.64 [0.64, 4.19]	N/S
Kumai (2017)	168	VAS: first step pain success	Hyaluronate –H (58)<br><br>Hyaluronate – VL (60)	18/58<br><br>19/60	OR: 0.97 (0.45, 2.11)	N/S
Kumai (2017)	168	Roles and Maudsley, success	Hyaluronate –L (50)<br><br>Hyaluronate – VL (60)	6/50<br><br>9/60	OR: 0.77 (0.25, 2.34)	N/S
Kumai (2017)	168	VAS: first step pain success	Hyaluronate –L (50)<br><br>Hyaluronate – VL (60)	19/50<br><br>19/60	OR: 1.32 (0.60, 2.91)	N/S
**Micronized dHACM versus saline injections**						
Cazzell (2018)	147	VAS	Micronized dHACM (73)<br><br>Sodium chloride (0.9%) (72)	17.1 (23.6)<br><br>38.8 (31.2)	-0.78 [-1.12, -0.44]	Favours dHACM
Cazzell (2018)	147	Foot Function Index–Revised (FFI-R)	Micronized dHACM (73)<br><br>Sodium chloride (0.9%) (72)	23.7 (23.6)<br><br>33.2 (26.2)	-0.38 [-0.71, -0.05]	Favours dHACM
**Platelet rich plasma versus platelet poor plasma**						
Malahias (2019)	36	VAS: pain	Platelet rich plasma (18)	42 (29.2)	0.13 (-0.53, 0.78)	N/S

			Platelet poor plasma (18)	38 (31.7)		
		VAS: function	Platelet rich plasma (18)<br><br>Platelet poor plasma (18)	58 (32.5)<br><br>56 (32.6)	0.06 (-0.59, 0.71)	N/S
		VAS: satisfaction	Platelet rich plasma (18)<br><br>Platelet poor plasma (18)	66 (34.8)<br><br>70 (30.5)	-0.12 (-0.77, 0.53)	N/S

Pulsed radiofrequency

Table: Short term effectiveness of pulsed radiofrequency for pain and function associated with plantar heel pain

Study	Population	Outcome measure	Interventions	Outcomes	Statistical analysis OR (95% CI) or SMD (95% CI) unless otherwise stated	Favours
**Ultrasound guided pulsed radiofrequency of tibial nerve versus ultrasound guided local anaesthetic injection**						
Wu (2017)	36	VAS: overall pain	Ultrasound guided pulsed radiofrequency of tibial nerve (18)<br><br>Ultrasound guided local anaesthetic injection (18)	1.54 (1.26)<br><br>6.09 (1.70)	-2.97 (-3.95, -2.00)	Ultrasound guided pulsed radiofrequency of tibial nerve
Wu (2017)	36	VAS, first step pain	Ultrasound guided pulsed radiofrequency of tibial nerve (18)<br><br>Ultrasound guided local anaesthetic injection (18)	1.79 (1.62)<br><br>6.13 (1.75)	-2.52 (-3.41, -1.62)	Ultrasound guided pulsed radiofrequency of tibial nerve
**Ultrasound guided pulsed radiofrequency of calf muscle versus sham**						
Ye (2015)	100	FHSQ: pain	Ultrasound guided pulsed radiofrequency of calf muscle (gastrocnemius trigger point) (50)<br><br>Sham (50)	-70.9 (23.5)<br><br>-50.6 (24.5)	-0.84 (-1.25, -0.43)	Ultrasound guided pulsed radiofrequency of calf muscle

Ye (2015)	100	VAS: first step pain	Ultrasound guided pulsed radiofrequency of calf muscle (50)<br><br>Sham (50)	36.4 (27.4)<br><br>62.6 (27.5)	-0.95 (-1.36, -0.53)	Ultrasound guided pulsed radiofrequency of calf muscle
Ye (2015)	100	FHSQ: function	Ultrasound guided pulsed radiofrequency of calf muscle (50)<br><br>Sham (50)	-81.3 (19.9)<br><br>-62.1 (23.5)	-0.87 (-1.29, -0.46)	Ultrasound guided pulsed radiofrequency of calf muscle

BMJ Publishing Group Limited (BMJ) disclaims all liability and responsibility arising from any reliance placed on this supplemental material which has been supplied by the author(s)

Taping and iontophoresis

Table: Short term effectiveness of low dye taping and iontophoresis for pain associated with plantar heel pain

Study	Population	Outcome measure	Interventions	Outcomes	Statistical analysis SMD (95% CI) or OR (95% CI) unless otherwise stated	Favours
Osborne (2006)	31	VAS: worst pain	Low dye taping, stretching and 6 treatments of iontophoresis over 2 weeks with Placebo (0.9% NaCl) (10)<br><br>Low dye taping, stretching and 6 treatments of iontophoresis over 2 weeks with 0.4% Dexamethasone (11)<br><br>Low dye taping, stretching and 6 treatments of iontophoresis over 2 weeks with 5% Acetic acid (10)	Data requested		

Taping

Table: Short term effectiveness of low dye taping for pain and function associated with plantar heel pain

Study	Population	Outcome measure	Interventions	Outcomes	Statistical analysis SMD (95% CI) or OR (95% CI) unless otherwise stated	Favours
Radford (2006)	92	VAS: First step pain	Low dye taping + sham ultrasound<br><br>Sham ultrasound	41.4 (28.5)<br><br>54.0 (24.8)	-0.47 (-0.88, -0.05)	Taping
Radford (2006)	92	FHSQ: pain	Low dye taping + sham ultrasound<br><br>Sham ultrasound	60.0 (22.0)<br><br>53.5 (21.0)	0.30 (-0.11, 0.71)	N/S
Radford (2006)	92	FHSQ: function	Low dye taping + sham ultrasound<br><br>Sham ultrasound	72.0 (21.9)<br><br>70.9 (25.0)	-0.05 (-0.46, 0.36)	N/S

Manual therapy and exercise

Table: Short term effectiveness of foot mobilisation + stretching + US versus stretching + ultrasound for pain and function associated with plantar heel pain.

Study	Population	Outcome measure	Interventions	Outcomes	Statistical analysis SMD (95% CI) or OR (95% CI) unless otherwise stated	Favours
**Foot mobilisation + stretching + ultrasound versus stretching + ultrasound**						
Shashua (2015)	50	VAS: first step pain	Foot mobilisation + stretching + ultrasound (25)<br><br>Stretching + ultrasound (25)	4.6 (3.3)<br><br>4.7 (3.4)	-0.02 (-0.58, 0.53)	N/S
Shashua (2015)	50	LEFS	Foot mobilisation + stretching + ultrasound (25)<br><br>Stretching + ultrasound (25)	55.9 (19.4)<br><br>57.8 (18.0)	-0.10 (-0.66, 0.45)	N/S
**Manual therapy and exercise (stretching) versus iontophoresis, ultrasound and exercise (stretch and strengthening)**						
Cleland (2009)	60	NPRS: overall pain (change from baseline)	Manual therapy and exercise (30)<br><br>Iontophoresis and exercise (30)	-2.9 (2.1)<br><br>-1.4 (1.6)	0.78 [0.26, 1.31]	Manual therapy and exercise

Cleland (2009)	60	LEFS: function	Manual therapy and exercise (30)<br><br>Iontophoresis and exercise (30)	-21.0 (14.2)<br><br>-7.5 (14.2)	-0.94 (-1.47, -0.40)	Manual therapy and exercise
Cleland (2009)	60	FAAM: function	Manual therapy and exercise (30)<br><br>Iontophoresis and exercise (30)	22.2 (19.0)<br><br>8.9 (14.1)	0.78 (0.26, 1.31)	Manual therapy and exercise

Exercise: Stretching

Table: Short term effectiveness of stretching for pain and function associated with plantar heel pain

Study	Population	Outcome measure	Interventions	Outcomes	Statistical analysis SMD (95% CI) or OR (95% CI) unless otherwise stated	Favours
Radford (2007)	92	VAS: First step pain	Stretching + sham ultrasound<br><br>Sham ultrasound	51.1 (29.1)<br><br>62.5 (29.5)	-0.39 (-0.80, 0.03)	Not significant
Radford (2007)	92	FHSQ: pain	Stretching + sham ultrasound<br><br>Sham ultrasound	50.9 (23.1)<br><br>50.8 (26.4)	0.00 (-0.40, 0.41)	Not significant
Radford (2007)	92	FHSQ: function	Stretching + sham ultrasound<br><br>Sham ultrasound	72.4 (23.6)<br><br>66.4 (26.2)	-0.24 (-0.65, 0.17)	Not significant

Acupuncture

Table: Short term effectiveness of acupuncture for pain and function associated with plantar heel pain

**Study**	**Population**	**Outcome measure**	**Interventions**	**Outcomes**	**Statistical analysis SMD (95% CI) or OR (95% CI) unless otherwise stated**	**Favours**
Cotchett (2014)	84	VAS: first step pain	Trigger point dry needling (41)<br><br>Sham dry needling (43)	20.9 (19.4)<br><br>29.9 (23.3)	-0.42 (-0.85, 0.02)	N/S
Cotchett (2014)	84	FHSQ: pain	Trigger point dry needling (41)<br><br>Sham dry needling (43)	-72.2 (18.9)<br><br>-65.7 (20.5)	-0.33 (-0.76, 0.10)	N/S
Cotchett (2014)	84	FHSQ: function	Trigger point dry needling (41)<br><br>Sham dry needling (43)	-77.2 (21.7)<br><br>-<br>79.5 (18.1)	0.11 (-0.31, 0.54)	N/S

Wheatgrass

Table: Short term effectiveness of wheatgrass for pain associated with plantar heel pain

Study	Population	Outcome measure	Interventions	Outcomes	Statistical analysis SMD (95% CI) or OR (95% CI) unless otherwise stated	Favours
Young (2006)	80	VAS: first step pain	Wheatgrass (42)<br>Placebo (38)	Requested data		
Young (2006)	80	FHSQ: pain	Wheatgrass (42)<br>Placebo (38)	Requested data		
Young (2006)	80	FHSQ: function	Wheatgrass (42)<br>Placebo (38)	Requested data		

Cyrotherapy ultrasound

Table: Short term effectiveness of cryotherapy ultrasound for pain associated with plantar heel pain

Study	Population	Outcome measure	Interventions	Outcomes	Statistical analysis SMD (95% CI) or OR (95% CI) unless otherwise stated	Favours
Costantino (2014)	102	VAS: worst pain	Cryotherapy ultrasound (42)<br><br>Cryotherapy (42)	3.2 (2.3)<br><br>6.2 (1.0)	-1.66 (-2.16, -1.16)	Ultrasound cryotherapy
Costantino (2014)	102	Effectiveness index (VAS score at baseline/VAS score at 3 months x 100%)	Cryotherapy ultrasound (42)<br><br>Cryotherapy (42)	18/42<br><br>0/42	OR: 64.18 (3.70, 1112.56)	Ultrasound cryotherapy

Radiation therapy

Table: Short term effectiveness of radiation therapy for pain

Study	Population	Outcome measure	Interventions	Outcomes	Statistical analysis SMD (95% CI) or OR (95% CI) unless otherwise stated	Favours
Niewald (2012)	66	VAS	High dose radiation therapy - total dose of 6.0 Gy applied in 6 fractions of 1.0 Gy twice weekly (29)<br><br>Low dose radiation therapy - total dose of 0.6 Gy applied in 6 fractions of 1.0 Gy twice weekly (33)	18.7 (23.5)<br><br>39.5 (26.9)	-0.81 (-1.33, -0.29)	Radiation therapy

Low level laser

Table: Short term effectiveness of low level laser for pain and function associated with plantar heel pain

Study	Population	Outcome measure	Interventions	Outcomes	Statistical analysis OR (95% CI) or SMD (95% CI) unless otherwise stated	Favours
**Low level laser versus placebo**						
Macias (2015)	69	VAS: first step pain	Low level laser (37)<br>Sham (32)	39.5 (27.9)<br>62.3 (18.2)	-0.94 (-1.44, -0.44)	Low level laser
Macias (2015)	69	FFI: disability	Low level laser (37)<br>Sham (32)	31.5 (19.6)<br>33.5 (20.3)	-0.10 (-0.57, 0.37)	N/S
Macias (2015)	69	FFI: activity limitations	Low level laser (37)<br>Sham (32)	9.7 (7.9)<br>9.8 (7.1)	-0.01 (-0.49, 0.46)	N/S
**High level laser versus low level laser**						
Ordahan (2018)	75	VAS: overall pain	Low level laser (35)<br>High level laser (35)	5.56 (2.1)<br>2.75 (1.8)	1.40 [0.88, 1.93]	Favours high level laser
		FAOS: ADL	Low level laser (35)<br>High level laser (35)	51.63 (20.2)<br>58.8 (20.5)	-7.17 [-16.70, 2.36]	N/S

Electrolysis

Table: Short term effectiveness of electrolysis for pain

Study	Population	Outcome measure	Interventions	Outcomes	Statistical analysis SMD (95% CI) or OR (95% CI) unless otherwise stated	Favours
Fernandez-Rodriguez (2018)	73	VAS: first step	Ultrasound-guided percutaneous needle electrolysis of the fascia (38)<br><br>Placebo puncture (29)	1.1 (0.9)<br><br>4.8 (1.6)	-2.92 [-3.63, -2.22]	Electrolysis
		FAAM	Ultrasound-guided percutaneous needle electrolysis of the fascia (38)<br><br>Placebo puncture (29)	78.2 (5.5)<br><br>52.3 (11.6)	2.95 [2.25, 3.66]	Electrolysis

5.4.Medium term results for pain and function

Foot orthoses

Table: Medium term effectiveness of orthoses for pain and function associated with plantar heel pain

Study	Population	Outcome measure	Interventions	Outcomes	Statistical analysis SMD (95% CI) or OR (95% CI) unless otherwise stated	Favours
**Custom orthoses versus sham orthoses**						
Oliveira (2015)	74	FHSQ: pain	Custom made orthoses (37)<br><br>Sham orthoses (37)	2.6 (2.5)<br><br>4.2 (3.2)	-0.55 (-1.02, -0.09)	N/S
Oliveira (2015)	74	FHSQ: function	Custom made orthoses (37)<br><br>Sham orthoses (37)	-86.0 (14.9)<br><br>-78.5 (22.8)	-0.39 (-0.85, 0.07)	N/S

ESWT

Table: Medium term effectiveness of ESWT for pain and function associated with plantar heel pain

Study	Population	Outcome measure	Interventions	Outcomes Mean (SD) or Success n/N	Statistical analysis OR (95% CI) or SMD (95% CI) unless otherwise stated	Favours
**Radial shockwave versus placebo**						
Ibrahim (2010)	50	VAS: >60% improvement in VAS from baseline	Radial (25)<br>Placebo (25)	25/25<br>4/25	OR:<br>243.67 (12.41, 4785.60)	Radial ESWT
Ibrahim (2010)	50	VAS: overall pain	Radial (25)<br>Placebo (25)	0.5 (0.1)<br>7.4 (0.5)	-3.77 (-4.72, -2.82)	Radial ESWT
**Radial versus plantar fascial stretching**						
Rompe (2010)	102	FFI item II: First step pain (mean change from baseline)	Radial (48)<br>Stretching (54)	-3.5 (2.8)<br>-5.2 (2.5)	0.64 (0.24, 1.04)	Stretching
**Radial versus ultrasound guided steroid**						
Hocaoglu (2017)	72	VAS: overall pain	Radial (36)<br>Ultrasound guided steroid (36)	20.0 (12.5)<br>40.0 (12.5)	1.58 (1.05, 2.12)	Radial ESWT

Hocaoglu (2017)	72	FFI: overall	Radial (36)<br><br>Ultrasound guided steroid (36)	41.6 (27.7)<br><br>55.4 (19.7)	0.56 (0.09, 1.04)	Radial ESWT
**Focused shockwave therapy versus placebo**						
Rompe (2003)	45	VAS: >50% improvement in first step VAS from baseline	Focused (22)<br><br>Placebo (23)	12/22<br><br>6/23	OR:<br>3.40 (0.97, 11.91)	N/S
Rompe (2003)	45	VAS: first step pain	Focused (22)<br><br>Placebo (23)	2.1 (2.0)<br><br>4.7 (1.9)	-1.31 (-1.96, -0.66)	Focused ESWT
Speed 2003	88	VAS: first step	Focused (46)<br><br>Placebo (42)	34.7 (33.4)<br><br>29.0 (30.0)	0.18 (-0.24, 0.60)	N/S
Abt (2002)	32	VAS: overall pain and first step pain<br><br>Roles and Maudsley	Focused (17)<br><br>Placebo (15)	Data not available		

Lithotripter

Table: Medium term effectiveness of lithotripter for **pain** associated with plantar heel pain

Study	Population	Outcome measure	Interventions	Outcomes	Statistical analysis	Favours
Dogramaci (2010)	50	VAS: overall pain	Pneumatic lithotripter (25)<br><br>Sham (25)	2.0 (1.6)<br><br>7.1 (1.5)	-3.11 (-3.95, -2.27)	Lithotripter
Dogramaci (2010)	50	Roles and Maudsley score (number of patients who achieved good and excellent scores)	Pneumatic lithotripter (25)<br><br>Sham (25)	23/25<br><br>6/25	OR: 36.42 [6.57, 201.70]	Lithotripter

Local injection

Table: Medium term effectiveness of local injections for pain and function associated with plantar heel pain

Study	Population	Outcome measure	Interventions	Outcomes	Statistical analysis SMD (95% CI) or OR (95% CI) unless otherwise stated	Favours
**Local corticosteroid versus placebo**						
Crawford (1999)	106	VAS: overall pain	Local steroid (1ml of 25mg/ml of prednisolone) and local anaesthetic (1ml of 2% lignocaine) (24)<br><br>Local anaesthetic (20)	2.4 (2.6)<br><br>3.3 (2.7)	-0.33 (-0.93, 0.26)	N/S
Crawford (1999a)	106	VAS: overall pain	Local steroid and local anaesthetic + tibial nerve block (22)<br><br>Local anaesthetic + tibial nerve block (13)	2.5 (3.2)<br><br>0.6 (1.1)	0.70 (-0.00, 1.41)	N/S
Johannsen (2019)	90	VAS: pain during function<br><br>FFI: function and pain	Corticosteroid injection (31)<br><br>Strength training (30)<br><br>Corticosteroid injection +	Unable to extract data from FFI and data not available		

			strength training (29)	for VAS scores from the author		
**Polydeoxyribonucleotide versus corticosteroid**						
Lee (2019)	44	VAS: pain	Polydeoxyribonucleotide (22) – 1.5 ml of Polydeoxyribonucleotide was injected into the heel weekly for 3 weeks<br><br>Corticosteroid – 20mg of triamcinolone + 3mls of 1% lidocaine at the first visit. 3mls of normal saline at weeks 2 and 3 (22)	3.5 (2.4)<br><br>3.0 (1.9)	0.23 (-0.37, 0.82)	N/S
	44	Manchester-Oxford Foot Questionnaire (MOXFQ)	Polydeoxyribonucleotide (22) – 1.5 ml of Polydeoxyribonucleotide was injected into the heel weekly for 3 weeks<br><br>Corticosteroid – 20mg of triamcinolone + 3mls of 1% lidocaine at the first visit. 3mls of normal saline at weeks 2 and 3. (22)	26.4 (12.6)<br><br>27.4 (14.7)	-0.07 (-0.66, 0.52)	N/S
**Platelet rich plasma versus platelet poor plasma**						
Malahias (2019)	36	VAS: pain	Platelet rich plasma (18)<br><br>Platelet poor plasma (18)	29 (34.4)<br><br>43 (37)	-0.38 (-1.04, 0.28)	N/S
		VAS: function	Platelet rich plasma (18)	46 (41.7)	0.15 (-0.51, 0.80)	N/S

			Platelet poor plasma (18)	40 (39.1)		
		VAS: satisfaction	Platelet rich plasma (18)<br><br>Platelet poor plasma (18)	73 (36.1)<br><br>68 (33.4)	0.14 (0.51, 0.79)	N/S

Pulsed radiofrequency

Table: Medium term effectiveness of pulsed radiofrequency for pain and function associated with plantar heel pain

Study	Population	Outcome measure	Interventions	Outcomes	Statistical analysis SMD (95% CI) or OR (95% CI) unless otherwise stated	Favours
**Ultrasound guided pulsed radiofrequency of calf muscle versus sham**						
Ye (2015)	100	FHSQ: pain	Ultrasound guided pulsed radiofrequency of calf muscle (gastrocnemius trigger point) (50)<br><br>Sham (50)	-64.9 (27.9)<br><br>-46.4 (21.9)	-0.73 (-1.14, -0.33)	Ultrasound guided pulsed radiofrequency of calf muscle
Ye (2015)	100	VAS: first step pain	Ultrasound guided pulsed radiofrequency of calf muscle (50)<br><br>Sham (50)	42.4 (28.8)<br><br>56.6 (25.0)	-0.52 (-0.92, -0.12)	Ultrasound guided pulsed radiofrequency of calf muscle
Ye (2015)	100	FHSQ: function	Ultrasound guided pulsed radiofrequency of calf muscle (50)<br><br>Sham (50)	-75.7 (23.0)<br><br>-61.7 (26.5)	-0.56 (-0.96, -0.16)	Ultrasound guided pulsed radiofrequency of calf muscle

Manual therapy and exercise

Table: Medium term effectiveness of manual therapy and exercise versus iontophoresis and exercise for pain and function associated with plantar heel pain.

**Study**	**Population**	**Outcome measure**	**Interventions**	**Outcomes**	**Statistical analysis SMD (95% CI) or OR (95% CI) unless otherwise stated**	**Favours**
**Manual therapy and exercise (stretching) versus iontophoresis, ultrasound and exercise (stretch and strengthening)**						
Cleland (2009)	60	NPRS: overall pain	Manual therapy and exercise (30)<br>Iontophoresis and exercise (30)	3.4 (2.9)<br>2.8 (2.4)	0.22 (-0.29, 0.73)	N/S
Cleland (2009)	60	LEFS: function	Manual therapy and exercise (30)<br>Iontophoresis and exercise (30)	22.8 (19.2)<br>12.9 (13.6)	0.58 (0.07, 1.10)	Manual therapy and exercise
Cleland (2009)	60	FAAM: function	Manual therapy and exercise (30)<br>Iontophoresis and exercise (30)	31.6 (25.1)<br>17.9 (13.3)	0.67 (0.15, 1.19)	Manual therapy and exercise

Electrolysis

Table: Short term effectiveness of electrolysis for pain

Study	Population	Outcome measure	Interventions	Outcomes	Statistical analysis SMD (95% CI) or OR (95% CI) unless otherwise stated	Favours
Fernandez-Rodriguez (2018)	73	VAS: first step	Ultrasound-guided percutaneous needle electrolysis of the fascia (38)<br><br>Placebo puncture (29)	0.4 (0.6)<br><br>3.7 (1.6)	-2.85 (-3.55, -2.16)	Electrolysis
		FAAM	Ultrasound-guided percutaneous needle electrolysis of the fascia (38)<br><br>Placebo puncture (29)	82.0 (2.9)<br><br>61.8 (12.7)	2.32 (1.69, 2.95)	Electrolysis

5.5.Long term results for pain and function

Orthoses

Study	Population	Outcome measure	Interventions	Outcomes	Statistical analysis SMD (95% CI) or OR (95% CI) unless otherwise stated	Favours
**Custom orthoses versus sham orthoses**						
Landorf (2006)	136	FHSQ: pain	Custom made orthoses (46)<br><br>Sham orthoses (46)	-83.1 (21.4)<br><br>-82.3 (18.0)	0.04 (-0.45, 0.37)	NS
Landorf (2006)	136	FHSQ: function	Custom made orthoses (46)<br><br>Sham orthoses (46)	-90.2 (17.8)<br><br>-87.8 (20.6)	-0.12 (-0.54, 0.29)	NS
**Custom orthoses versus prefabricated orthoses**						
Landorf (2006)	136	FHSQ: pain	Custom made orthoses (46)<br><br>Prefabricated orthoses (44)	-83.1 (21.4)<br><br>-83.8 (18.0)	0.04 (-0.38, 0.45)	NS
Landorf (2006)	136	FHSQ: function	Custom made orthoses (46)<br><br>Prefabricated orthoses (44)	90.2 (17.8)<br><br>89.5 (19.0)	0.04 (-0.38, 0.45)	NS

ESWT

Table: Long term effectiveness of ESWT for **pain and function** associated with plantar heel pain

Study	Population	Outcome measure	Interventions	Outcomes Mean (SD) or Success n/N	Statistical analysis OR (95% CI) or SMD (95% CI) unless otherwise stated	Favours
**Radial shockwave versus placebo**						
Ibrahim (2016)	50	VAS: >60% improvement in VAS from baseline	Radial (25)<br><br>Placebo (25)	22/25<br><br>6/25	OR:<br>23.22 (5.10, 105.73)	Radial ESWT
Ibrahim (2016)	50	VAS: overall pain	Radial (25)<br><br>Placebo (25)	-6.2 (8.2)<br><br>-2.04 (2.7)	-0.66 (-1.24, -0.09)	Radial ESWT
**Radial versus plantar fascial stretching**						
Rompe (2010)	102	FFI item II: First step pain (mean change from baseline)	Radial (48)<br><br>Stretching (54)	-5.9 (2.6)<br><br>-5.8 (2.3)	-0.04 (-0.43, 0.35)	N/S
**Focused shockwave therapy versus placebo**						
Rompe (2003)	45	VAS: >50% improvement in first step VAS from baseline	Focused (22)<br><br>Placebo (23)	13/22<br><br>7/23	OR: 3.3 (0.97, 11.29)	N/S
Rompe (2003)	45	VAS: first step pain	Focused (22)	1.5 (1.7)	-1.67 (-2.45, -0.88)	Focused ESWT

			Placebo (23)	4.4 (1.7)		
Abt (2002)						Unable to extract data

Cryotherapy ultrasound

Table: Long term effectiveness of ultrasound cryotherapy for pain associated with plantar heel pain

Study	Population	Outcome measure	Interventions	Outcomes	Statistical analysis SMD (95% CI) or OR (95% CI) unless otherwise stated	Favours
Costantino (2014)	102	VAS: worst pain	Cryotherapy ultrasound (42)<br><br>Cryotherapy (42)	1.5 (2.06)<br><br>6.3 (0.9)	-2.98 (-3.61, -2.35)	Ultrasound cryotherapy
Costantino (2014)	102	Effectiveness index (VAS score at baseline/VAS score at 3 months x 100%)	Cryotherapy ultrasound (42)<br><br>Cryotherapy (42)	36/42<br><br>0/42	OR: 477.31 [26.00, 8764.02]	Ultrasound cryotherapy

Radiation therapy

Table: Long term effectiveness of radiation therapy for pain

**Study**	**Population**	**Outcome measure**	**Interventions**	**Outcomes**	**Statistical analysis SMD (95% CI) or OR (95% CI) unless otherwise stated**	**Favours**
Niewald (2012)	66	VAS	High dose radiation therapy - total dose of 6.0 Gy applied in 6 fractions of 1.0 Gy twice weekly (29)<br><br>Low dose radiation therapy - total dose of 0.6 Gy applied in 6 fractions of 1.0 Gy twice weekly (33)	Requested data		

5.6.Interventions with neutral evidence of efficacy

Prefabricated foot orthoses

Two trials evaluated the efficacy of prefabricated foot orthoses. Landorf[2] in a three arm trial that also included custom made foot orthoses, evaluated the effectiveness of a firm density polyethylene foam compared to a 6mm soft ethyl vinyl acetate flat insole. In contrast, Wrobel3 compared a 45 durometer ethyl vinyl acetate orthotic with a neoprene topcover versus a 35 durometer 3mm ethyl vinyl acetate flat insole with a neoprene cover.

The trial by Landorf[2] met all the quality and power criteria to be considered for primary proof of efficacy, while the trial by Wrobel[3] did not have the required power but was of adequate quality for inclusion in a meta-analysis. Pooling revealed moderate evidence of no effect for pain in the short term (SMD -0.25 95% CI -0.59 to 0.09) and long term (SMD -0.08 [-0.50, 0.33] and moderate evidence of no effect for foot function (SMD -0.06 95% CI -0.40 to 0.28) in the short term and long term (SMD -0.08 95% CI -0.50 to 0.33).

Magnetised insoles

One trial,[4] which met all the quality and power criteria, evaluated the efficacy of magnetised insoles versus sham insoles for pain. The results revealed moderate evidence of no effect for pain in the short term (SMD: 0.00 95% CI -0.39 to 0.39). Minor adverse events were reported by 27% of participants in the sham control group while 13% reported adverse events in the magnetised insole group primarily related to issues accommodating the insoles in shoes. No qualitative data was provided to support the use of magnetised insoles.

Wheat grass

Young[5] evaluated the efficacy of wheatgrass versus placebo in the short term, although data was not available on request.

Corticosteroid injections

Seven trials included a corticosteroid injection in one of the treatment arms. Three trials[6-8] attempted to isolate the specific effect of the active ingredient (i.e. the corticosteroid) by comparing it to treatment arms that included a saline injection, while Ahmed[9] compared methylprednisolone to dexamethasone, Babaei-Ghazani[10] evaluated the effectiveness of ozone (O2-O3) versus methylprednisolone, Diaz-Llopis[11] compared betamethasone to Botulium toxin and Hocaoglu[54] compared betamethasone to radial ESWT.

No trial was considered for primary proof of efficacy as there were no control arms that included a placebo, sham or no treatment group. Importantly, a treatment arm that included an injection, with penetration of the skin, was not considered to be a true sham or placebo control group. Furthermore, no trial had sufficient power to be considered for secondary proof of efficacy.

One trial, which evaluated the effectiveness of a single ultrasound guided corticosteroid injection plus local anaesthesia versus a saline injection plus local anaesthesia[6] met the quality and power criteria. However, the trial did not compare a corticosteroid injection to a true placebo, sham or no treatment control, or to a treatment of proven efficacy. As this trial compared a corticosteroid injection to another unproven intervention, and no difference in effect was noted in the short term, the intervention is regarded as not being adequately tested.

There was tension in the qualitative data regarding the use of corticosteroid injections with some experts suggesting it might be the ticket to positive outcomes, and can be difficult to resist for patients that want a quick result. In contrast, others suggested it should be best avoided with a greater focus on rehabilitation. Experts also reported that the local anaesthetic effects, associated with the injection, might mislead patients and could be considered as the important element in outcomes. A sub-theme also emerged that corticosteroid injections only provide short term relief and should be weighed up against the risk of adverse events such as rupture, fat pad atrophy and patients getting worse in the long term.

Manual therapy

SSI results were confirmed by the systematic review, with findings suggesting only occasional use of soft tissue mobilisation techniques and these being of low priority and variable effect. Two studies evaluated the effectiveness of a myriad of manual therapy and exercise interventions although neither study could be considered for primary or secondary proof of efficacy. Cleland[12] found that manual therapy and exercise was superior for pain versus a group that received iontophoresis, cryotherapy and exercise in the short term (SMD 0.78, 95% CI 0.26 to 1.31) but not in the medium (SMD 0.22, 95% CI -0.29 to 0.73). However, neither set of interventions has secondary proof of efficacy and it is unclear if the difference between groups for pain reflected a deleterious effect associated with the interventions in the group with less superior outcomes.

A second trial,[13] that did not meet the power criteria found that the addition of foot and ankle mobilizations to a program including stretching and ultrasound was not superior to stretching and ultrasound alone for first step pain (SMD -0.02 95% CI -0.58 to 0.53) or function (SMD -0.10 95% CI -0.66 to 0.45) in the short term.

Rehabilitative exercise

No exercise rehabilitation studies met the inclusion criteria, which contrasted strongly with the SSI results, consistent with this being an under-researched area. Perhaps reflecting the lack of level 1 evidence, there were tensions in the findings between local and whole kinetic chain approaches, and how best to manage overall tissue load. Many specific exercise pointers were found, that may guide future RCTs.

References

1. O'Cathain A, Murphy E, Nicholl J. The quality of mixed methods studies in health services research. 2008;13:92-98.
2. Landorf KB, Keenan AM, Herbert RD. Effectiveness of foot orthoses to treat plantar fasciitis: a randomized trial. *Arch Intern Med.* 2006;166:1305-1310.
3. Wrobel JS, Fleischer AE, Crews RT, Jarrett B, Najafi B. A randomized controlled trial of custom foot orthoses for the treatment of plantar heel pain. *J Am Podiatr Med Assoc.* 2015;105:281-294.
4. Winemiller MH, Billow RG, Laskowski ER, Harmsen WS. Effect of magnetic vs sham-magnetic insoles on plantar heel pain: a randomized controlled trial. *JAMA.* 2003;290:1474-1478.
5. Young MA, Cook JL, Webster KE. The effect of topical wheatgrass cream on chronic plantar fasciitis: a randomized, double-blind, placebo-controlled trial. *Complement Ther Med.* 2006;14:3-9.
6. McMillan AML, K. B.; Gilheany, M. F.; Bird, A. R.; Morrow, A. D.; Menz, H. B. Ultrasound guided corticosteroid injection for plantar fasciitis: randomised controlled trial. *BMJ.* 2012;344:e3260.
7. Ball EM, McKeeman HM, Patterson C, et al. Steroid injection for inferior heel pain: a randomised controlled trial. *Ann Rheum Dis.* 2013;72:996-1002.
8. Crawford F, Atkins D, Young P, Edwards J. Steroid injection for heel pain: evidence of short-term effectiveness. A randomized controlled trial. *Rheumatol (Oxford).* 1999;38:974-977.
9. Ahmed GS, Shaikh AH, Tofique M. Local steroid injection for treatment of plantar fasciitis. Comparison between methylprednisolone and dexamethasone. *Med Channel.* 2013;19:37-41.
10. Babaei-Ghazani A, Karimi N, Forogh B, et al. Comparison of ultrasound-guided local ozone (O2-O3) injection vs corticosteroid injection in the treatment of chronic plantar fasciitis: a randomized clinical trial. *Pain Med.* 2018;doi: 10.1093/pm/pny066. [Epub ahead of print].
11. Diaz-Llopis IV, Gomez-Gallego D, Mondejar-Gomez FJ, Lopez-Garcia A, Climent-Barbera JM, Rodriguez-Ruiz CM. Botulinum toxin type A in chronic plantar fasciitis: clinical effects one year after injection. *Clin Rehabil.* 2013;27:681-685.
12. Cleland JA, Abbott JH, Kidd MO, et al. Manual physical therapy and exercise versus electrophysical agents and exercise in the management of plantar heel pain: a multicenter randomized clinical trial. *J Orthop Sports Phys Ther.* 2009;39:573-585.
13. Shashua A, Flechter S, Avidan L, Ofir D, Melayev A, Kalichman L. The effect of additional ankle and midfoot mobilizations on plantar fasciitis: a randomized controlled trial. *J Orthop Sports Phys Ther.* 2015;45:265-272.

5.7.Forest plots

Radial ESWT versus sham (overall pain in the short term)

	Radial ESWT			Sham				Std. Mean Difference
**Study or Subgroup**	**Mean**	**SD**	**Total**	**Mean**	**SD**	**Total**	**Weight**	**IV, Random, 95% CI**
Gerdesmeyer 2008	-56	39.3	125	-44.1	41.8	118	51.1%	-0.29 [-0.55, -0.04]
Ibrahim 2010	-7.44	2.45	25	-1.2	1.47	25	48.9%	-3.04 [-3.87, -2.21]
**Total (95% CI)**			**150**			**143**	**100.0%**	**-1.64 [-4.33, 1.06]**

Heterogeneity: $Tau^2 = 3.68$; $Chi^2 = 38.32$, df = 1 ($P < 0.00001$); $I^2 = 97\%$
Test for overall effect: Z = 1.19 (P = 0.23)

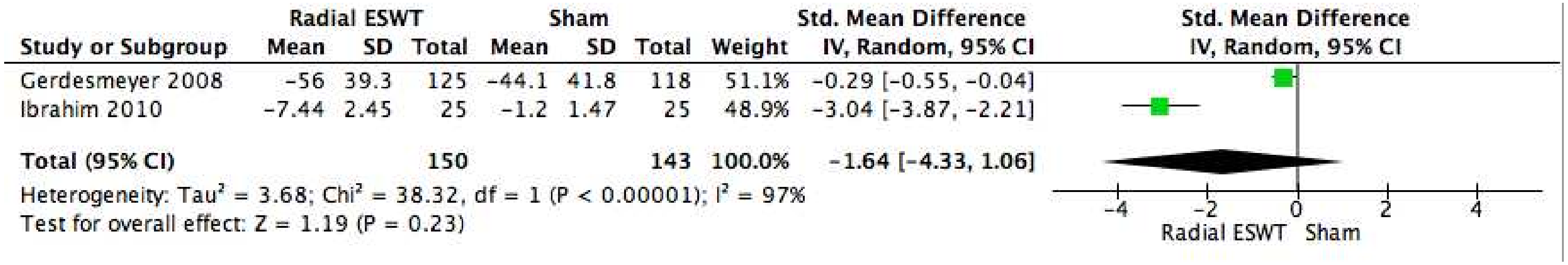

Focused ESWT versus sham (first step pain in the short term)

	ESWT		Sham			Odds Ratio
**Study or Subgroup**	**Events**	**Total**	**Events**	**Total**	**Weight**	**M-H, Random, 95% CI**
Gollwitzer 2007	11	20	6	20	13.3%	2.85 [0.78, 10.47]
Gollwitzer 2015	63	125	44	121	86.7%	1.78 [1.07, 2.96]
**Total (95% CI)**		**145**		**141**	**100.0%**	**1.89 [1.18, 3.04]**
Total events	74		50			

Heterogeneity: $Tau^2 = 0.00$; $Chi^2 = 0.44$, df = 1 ($P = 0.51$); $I^2 = 0\%$
Test for overall effect: Z = 2.64 (P = 0.008)

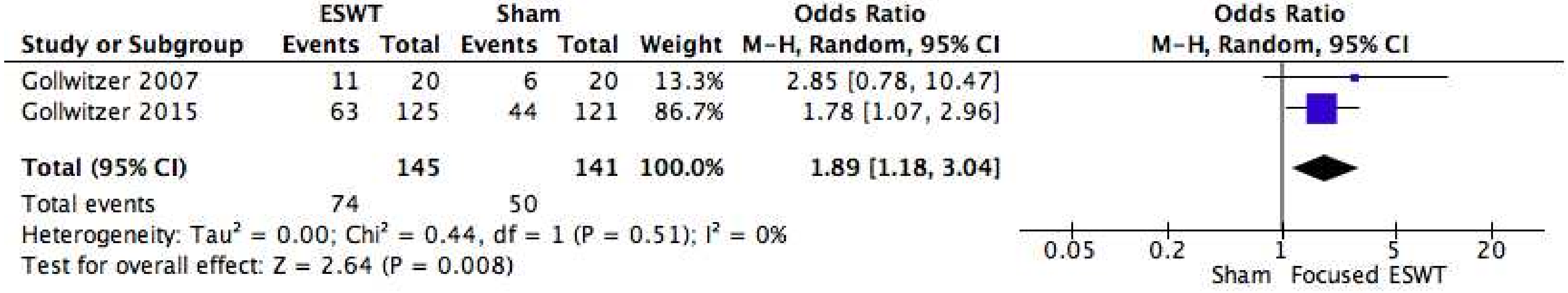

Custom foot orthoses versus sham (overall pain in the short term)

	Custom foot orthoses			Sham foot orthoses				Std. Mean Difference
Study or Subgroup	Mean	SD	Total	Mean	SD	Total	Weight	IV, Random, 95% CI
Bishop 2018	15.3	13.9	20	38.3	26.7	20	17.9%	−1.06 [−1.73, −0.39]
Landorf 2006	−71.8	20.6	46	−63.4	21.5	46	31.5%	−0.40 [−0.81, 0.02]
Oliveira 2015a	3.5	2.7	37	4.2	3.2	37	28.4%	−0.23 [−0.69, 0.22]
Wrobel 2015	22.4	9.31	25	23.5	8.64	23	22.2%	−0.12 [−0.69, 0.45]
**Total (95% CI)**			**128**			**126**	**100.0%**	**−0.41 [−0.74, −0.07]**

Heterogeneity: $Tau^2 = 0.05$; $Chi^2 = 5.19$, df = 3 (P = 0.16); $I^2 = 42\%$
Test for overall effect: Z = 2.37 (P = 0.02)

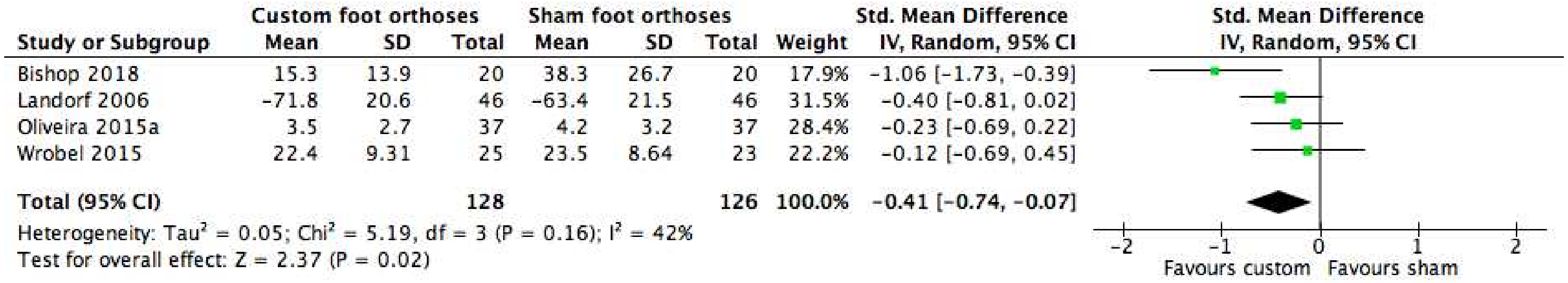

5.8. Risk of Bias 2

Results of Risk of Bias 2 are presented for studies informing the determination of primary or secondary proof of efficacy. Plot 1 is the agreed summary from all three reviewers (MC, DM, CB) with the most stringent reviewer producing plot 2.

Plot 1: ROB2 - agreed summary

Study	Experimental	Comparator	Outcome	Weight	Randomization process	Deviations from intended interve	Missing outcome data	Measurement of the outcome	Selection of the reported result	Overall
Landorf 2006	Orthoses	sham	All PROMs	1	+	+	+	+	+	+
Oliveira 2015	orthoses	sham	All PROMs	1	+	+	+	+	+	+
WROBEL 2015A	Orthoses	Sham	All PROMs	1	+	+	+	+	?	!
BISHOP 2018	Orthoses	sham	All PROMs	1	+	+	+	+	+	+
Gerdesmeyer 2008	ESWT	Placebo	All PROMs	1	+	+	+	+	+	+
Ibrahim 2010	ESWT	sham	All PROMs	1	+	+	+	+	+	+
Gollwitzer 2007	ESWT	sham	All PROMs	1	+	+	+	+	+	+
Gollwitzer 2015	ESWT	sham	All PROMs	1	+	+	+	+	+	+
ROMPE 2010	Stretching	ESWT	All PROMs	1	+	+	+	?	+	!
Radford 2006	Taping	no tape	All PROMs	1	+	+	+	+	+	+

+ Low risk
? Some concerns
– High risk

5.9. Risk of Bias 2: Risk of Bias – Support for Judgements

Study: Landorf (2006)

Risk of bias		
Bias	Author's judgement	Support for judgement
Randomization process	Low risk	Participants were allocated to 1 of 3 groups according to a computer-generated random allocation sequence.<br><br>The allocation sequence was concealed from potential participants and from the investigator who recruited participants<br><br>There were no baseline differences in key outcomes
Deviations from intended interventions	Low risk	It is unlikely participants were aware of their assigned intervention as they all received a similar assessment and had a neutral position cast taken.<br><br>Carers and people delivering the intervention were aware of the participant's intended intervention, however, there were no deviations from the intended intervention that arose because of the experimental context.

		ITT was used to estimate the effect of assignment to intervention.
Missing outcome data	Low risk	Data for pain and function were available for all, or nearly all participants randomised.<br><br>Loss to follow-up over the 12 months of the trial was 2.9%.
Measurement of the outcome data	Low risk	A study protocol was published and the results included all major outcomes. The main outcome measure was the FHSQ which is a reliable and valid outcome measure<br><br>Comparable methods of outcome measurement involving the same measurement methods and thresholds were used at comparable time points.<br><br>As the outcomes were participant reported and the participants were blinded to their intervention, the outcome assessor (i.e. the participant) was blinded.

		Also, "To minimize the assessor's influence on participant responses, all outcomes were measured at the beginning of each appointment prior to any interaction between the participant and the assessor"
Selection of the reported result	Low risk	Data was analysed in accordance with a pre-specified analysis plan as highlighted on the ANZCTR (ACTRN12606000091505)<br><br>All eligible reported results of the pain and function domains corresponded to all intended outcome measurements.<br><br>All eligible reported results for the outcome measurements correspond to all the intended analyses

Study: Wrobel (2015)

Risk of bias		
Bias	Author's judgement	Support for judgement
Randomization process	Low risk	Patients were block

		randomized (n = 6 in each center; created, concealed, and assigned by the research coordinator.<br><br>There were no baseline differences in key outcomes
Deviations from intended interventions	Low risk	It is unlikely participants were aware of their assigned intervention as they all received a similar assessment and had a neutral position cast taken.<br><br>Carers and people delivering the intervention were aware of the participant's intended intervention, however there were no deviations from the intended intervention that arose because of the experimental context.<br><br>ITT was not used to estimate the effect of assignment to intervention, however the risk of a substantial impact of the failure to analyse participants in the group to which they were assigned could be considered to be low.
Missing outcome data	Low risk	Data for pain and function

		were available for all, or nearly all participants randomised.<br><br>Loss to follow-up over the 3 months of the trial was 8.0%.
Measurement of the outcome data	Low risk	The main outcome was the FFI-R which is a reliable and valid outcome measure.<br><br>Comparable methods of outcome measurement involving the same measurement methods and thresholds were used at comparable time points.<br><br>As the outcomes were participant reported and the participants were blinded to their intervention, the outcome assessor (i.e. the participant) was blinded.
Selection of the reported result	Some concerns	The trail was registered at ClinicalTrials.gov (NCT00765843) but it is uncertain if all eligible reported results for the outcome domain correspond to all intended outcome measurements as there is very little information published in

		the registry about the outcome measurements and analysis

Study: Bishop (2019)

Risk of bias		
Bias	Author's judgement	Support for judgement
Randomization process	Low risk	"Group allocation was conducted via a researcher blind to recruitment using a computer generated block (4 × 15 blocks) random number sequence, after the initial assessment outlined above. Participants were blinded as to the exact nature of the trial, and simply told that the trial was investigating the effect of three different insoles in treating plantar heel pain"<br><br>There were no baseline differences in key outcomes
Deviations from intended interventions	Low risk	It is unlikely participants were aware of their assigned intervention as they all received a similar assessment and had a neutral position cast taken.<br><br>Carers and people delivering the intervention were aware of the participant's intended intervention, however there were no deviations reported

		from the intended intervention that arose because of the experimental context.<br><br>ITT was used to estimate the effect of assignment to intervention.
Missing outcome data	Low risk	Data for pain and function were available for all, or nearly all participants randomised.<br><br>Loss to follow-up over the 3 months of the trial was 6%.
Measurement of the outcome data	Low risk	A study protocol was published and the results included all major outcomes. The main outcome measure was a VAS which is a reliable and valid outcome measure of pain.<br><br>Comparable methods of outcome measurement involving the same measurement methods and thresholds were used at comparable time points.<br><br>As the outcomes were participant reported and the participants were blinded to

		their intervention, the outcome assessor (i.e. the participant) was blinded.<br><br>In addition, "A blinded assessor was used to process all outcome data".
Selection of the reported result	Low risk	Data was analysed in accordance with a pre-specified analysis plan as highlighted in the Australian New Zealand Clinical Trials Registry (ACTRN 12613000446763<br><br>All eligible reported results of the pain and function domains corresponded to all intended outcome measurements.<br><br>All eligible reported results for the outcome measurements correspond to all the intended analyses

Study: Oliviera (2015)

Risk of bias		
Bias	Author's judgement	Support for judgement
Randomization process	Low risk	"A computer-generated randomization list with a 1:1 ratio created by a statistician not involved in the trial was used to allocate patients randomly to the study group (SG) or the control group (CG).<br><br>Blinded randomization was performed using opaque-sealed envelopes, and an independent researcher not involved in the eligibility assessment, outcome assessment, or treatment kept the assignment scheme in a locked cupboard in a central location".<br><br>There were no baseline differences in key outcomes.
Deviations from intended interventions	Low risk	It is unlikely participants were aware of their assigned intervention as they all received a similar assessment and had a negative cast taken of the foot.

		Carers and people delivering the intervention were aware of the participant's intended intervention, however there were no deviations reported from the intended intervention that arose because of the experimental context.<br><br>ITT was used to estimate the effect of assignment to intervention.
Missing outcome data	Low risk	Data for pain and function were available for all, or nearly all participants randomised.<br><br>Loss to follow-up over the 180 days of the trial was 4%.
Measurement of the outcome data	Low risk	A study protocol was published and the results included all major outcomes. The main outcome measure was a VAS and FHSQ which is a reliable and valid outcome measure of pain.<br><br>Comparable methods of outcome measurement involving the same measurement methods and thresholds were used at

		comparable time points.<br><br>As the outcomes were participant reported and the participants were blinded to their intervention, the outcome assessor (i.e. the participant) was blinded.
Selection of the reported result	Low risk	Data was analysed in accordance with a pre-specified analysis plan as highlighted in the This trial is registered on ClinicalTrials.gov (NCT01549678)<br><br>All eligible reported results of the pain and function domains corresponded to all intended outcome measurements.<br><br>All eligible reported results for the outcome measurements correspond to all the intended analyses

Study: Gerdesmeyer (2008)

Risk of bias		
Bias	Author's judgement	Support for judgement
Randomization process	Low risk	"A total of 254 patients were randomly assigned to receive either rESWT or placebo treatment with concealed allocation in permuted blocks of 4 to 8, stratified by treatment center with the use of a computer-generated random list".<br><br>There were no baseline differences in key outcomes
Deviations from intended interventions	Low risk	It is unlikely participants were aware of their assigned intervention as "Patients in the control group received identical placebo intervention with a placebo hand-piece that prevented transmission of shock waves. The placebo hand-piece was identical in design, shape, and weight to ensure that there was no way to identify the placebo hand-piece. The treatment in the placebo group was the same compared with the active one. Thereby, set up and sound created by the shock wave device was identical in both groups; however, no energy was administered in the placebo group".<br><br>Carers and people delivering the intervention were aware of the participant's intended intervention.

		There was no deviations from the intended intervention that arose because of the experimental context.<br><br>ITT was used to estimate the effect of assignment to intervention.
Missing outcome data	Low risk	Data for pain and function were available for all, or nearly all participants randomised at 12 weeks.<br><br>Loss to follow-up over the 12 weeks of the trial was 10% (n = 26)<br><br>For the ITT population at 12 weeks, 3% were lost to follow up.<br><br>For the dichotomous outcomes (overall success rate, first step pain) the observed number of successful events (n=126) is much greater than the number of participants with missing outcome data (n=26). Therefore, the risk of bias is considered low.
Measurement of the outcome data	Low risk	The main outcome measure was a VAS which is a reliable and valid outcome measure.<br><br>Comparable methods of outcome measurement involving the same measurement methods and thresholds were used at comparable time points.

		As the outcomes were participant reported and the participants were blinded to their intervention, the outcome assessor (i.e. the participant) was blinded.
Selection of the reported result	Low risk	Evidence of a pre-specified analysis plan was not found but the trial was conducted as an FDA approval study. In addition, the results included all major outcomes, thus the risk of reporting bias was probably low.

Study: Gollwitzer (2007)

Risk of bias		
Bias	Author's judgement	Support for judgement
Randomization process	Low risk	"Participants who were eligible and provided written, informed consent were randomized by permuted blocks of different length to receive either active treatment or placebo. Random allocation was guaranteed by consecutive sealed and nontransparent envelopes, which provided treatment allocation assigned by a computer-generated random list"<br><br>There were no baseline differences in key outcomes
Deviations from intended interventions	Low risk	It is unlikely participants were aware of their assigned intervention as "Participants in the control group received identical placebo therapy. An air-chambered polyethylene foil was located between the coupling head and the participant, which absorbed all the acoustic energy. Thereby, setup and sound created by the shockwave device was identical in both groups; however, transmission of shockwaves was prevented in the placebo group".<br><br>Carers and people delivering the intervention were aware of the

		participant's intended intervention.<br><br>There was no deviations from the intended intervention that arose because of the experimental context.<br><br>ITT was used to estimate the effect of assignment to intervention.
Missing outcome data	Low risk	Data for pain and function were available for all, or nearly all participants randomised at 12 weeks.<br><br>Loss to follow-up over the 12 weeks of the trial was 2% (n = 1)
Measurement of the outcome data	Low risk	The main outcome measure was a VAS which is a reliable and valid outcome measure.<br><br>Comparable methods of outcome measurement involving the same measurement methods and thresholds were used at comparable time points.<br><br>As the outcomes were participant reported and the participants were blinded to their intervention, the outcome assessor (i.e. the participant) was blinded.
Selection of the reported result	Low risk	Evidence of a pre-specified analysis plan was not found however the results included all major outcomes, thus the risk of reporting bias was probably low.

BMJ Publishing Group Limited (BMJ) disclaims all liability and responsibility arising from any reliance placed on this supplemental material which has been supplied by the author(s)

Study: Gollwitzer (2015)

Risk of bias		
Bias	Author's judgement	Support for judgement
Randomization process	Low risk	"Participants who were eligible and provided written, informed consent were randomized by permuted blocks of different length to receive either active treatment or placebo. Random allocation was guaranteed by consecutive sealed and nontransparent envelopes, which provided treatment allocation assigned by a computer-generated random list"<br><br>There were no baseline differences in key outcomes
Deviations from intended interventions	Low risk	It is unlikely participants were aware of their assigned intervention as "The placebo group received identical sham intervention with an airfilled standoff that prevented the transmission of shock waves. The placebo handpiece was identical in design, shape, and weight to ensure that there was no way for the participants to identify the placebo handpiece".<br><br>Carers and people delivering the intervention were aware of the participant's intended intervention.<br><br>There was no deviations from the

		intended intervention that arose because of the experimental context.<br><br>ITT was used to estimate the effect of assignment to intervention.
Missing outcome data	Low risk	Loss to follow-up over the 12 weeks of the trial was 6.8% (n = 17)<br><br>For the ITT population at 12 weeks, 1.6% were lost to follow up.<br><br>For the dichotomous outcomes (overall success rate, first step pain) the observed number of successful events (n=112) is much greater than the number of participants with missing outcome data (n=17), so the risk of bias is considered low.
Measurement of the outcome data	Low risk	The main outcome measure was a VAS which is a reliable and valid outcome measure.<br><br>Comparable methods of outcome measurement involving the same measurement methods and thresholds were used at comparable time points.<br><br>As the outcomes were participant reported and the participants were blinded to their intervention, the outcome assessor (i.e. the participant) was blinded.

Selection of the reported result	Low risk	Evidence of a pre-specified analysis plan was not found however the trial was registered and was conducted as a U.S. Food and Drug Administration (FDA) approval study (Investigational Device Exemption number IDE G050236), thus the risk of reporting bias was probably low.

Study: Ibrahim (2016)

Risk of bias		
Bias	Author's judgement	Support for judgement
Randomization process	Low risk	Randomization was performed by a computerized random number generator created by an independent bio-statistician to draw up groups' allocation. An administrative assistant distributed interventions via opaque, sealed envelopes, containing information about the individual allocation schedule.<br><br>There were no baseline differences in key outcomes
Deviations from intended interventions	Low risk	It is unlikely participants were aware of their assigned intervention as "The patients were not made aware as to whether they received RSWT or placebo treatment. The principal investigator who applied the treatments prevented any behavior that could have indicated to the patients whether they received RSWT or placebo treatment. Specifically, (i) he did not address this issue to the patients; (ii) no patient knew how placebo treatment was actually achieved; (iii) the sound, look and handling of the RSWT device were identical in both RSWT and placebo treatments; and (iv) all RSWT or placebo treatment sessions took approximately ten minutes. Thus, the patients could not

		determine whether they received RSWT or placebo treatment”.<br><br>Carers and people delivering the intervention were aware of the participant’s intended intervention.<br><br>There was no deviations from the intended intervention that arose because of the experimental context.<br><br>ITT was used to estimate the effect of assignment to intervention.
Missing outcome data	Low risk	Loss to follow-up over the 2 year period of the trial was 6% (n = 3)
Measurement of the outcome data	Low risk	The main outcome measure was a VAS which is a reliable and valid outcome measure.<br><br>Comparable methods of outcome measurement involving the same measurement methods and thresholds were used at comparable time points.<br><br>As the outcomes were participant reported and the participants were blinded to their intervention, the outcome assessor (i.e. the participant) was blinded.
Selection of the reported result	Low risk	All eligible reported results of the pain and function domains corresponded to all intended outcome measurements.

		All eligible reported results for the outcome measurements correspond to all the intended analyses<br><br>It has been registered with ClinicalTrials.gov (Identifier NCT02679521)

Study: Rompe (2010)

Risk of bias		
Bias	Author's judgement	Support for judgement
Randomization process	Low risk	"A computerized random-number generator was used to formulate an allocation schedule. Patients were allocated to treatment groups in blocks of six. A medical assistant allocated interventions according to the allocation schedule. The medical assistant was unaware of the size of the blocks. It was not possible to blind the individual patient to his or her treatment assignment at any point during the study".<br><br>There were no baseline differences in key outcomes
Deviations from intended interventions	Low risk	Participants and people delivering the interventions were aware of the assigned intervention. However, there was no deviations from the intended intervention that arose because of the experimental context.<br><br>ITT was used to estimate the effect of assignment to intervention.
Missing outcome data	Low risk	Data for pain and function were available for nearly all participants randomised at 12 weeks.<br><br>Loss to follow-up over the 8 weeks

		(primary endpoint) of the trial was 5.8% (n = 6)
Measurement of the outcome data	Some concerns	The main outcome measure was the FFI, which is a reliable and valid outcome measure.<br><br>Comparable methods of outcome measurement involving the same measurement methods and thresholds were used at comparable time points. However, it is unclear if outcome assessors were aware of the intervention received by the participants and it is possible that assessment of the outcome could have been influenced by knowledge of the intervention received.
Selection of the reported result	Low risk	Evidence of a pre-specified analysis plan was found at Current Controlled Trials (http://www.controlled-trials.com/ISRCTN03438342.<br><br>All eligible reported results of the pain and function domains corresponded to all intended outcome measurements.<br><br>All eligible reported results for the outcome measurements correspond to all the intended analyses

BMJ Publishing Group Limited (BMJ) disclaims all liability and responsibility arising from any reliance placed on this supplemental material which has been supplied by the author(s)

Study: Radford (2006)

Risk of bias		
Bias	Author's judgement	Support for judgement
Randomization process	Low risk	The random allocation sequence was generated using a computer program (Microsoft Excel) in one block (i.e. simple randomisation). The allocation sequence was concealed from the researcher (JR) enrolling and assessing participants in sequentially numbered, opaque, sealed and stapled envelopes.<br><br>There were no baseline differences in key outcomes
Deviations from intended interventions	Low risk	It is unlikely participants were aware of their assigned intervention. "Participants were informed prior to entering the study that a sham intervention was being administered in the trial and were blinded as to whether they were receiving active treatment or not". "Care was taken that study participants did not meet by ensuring they exited the building by a different door- way to the one through which they entered".<br><br>There was no deviations from the intended intervention that arose because

		of the experimental context.<br><br>ITT was used to estimate the effect of assignment to intervention.
Missing outcome data	Low risk	Data for pain and function were available for all, or nearly all participants randomised.<br><br>Loss to follow-up over the course of the trial was 0.0%.

Measurement of the outcome data	Low risk	A study protocol was published and the results included all major outcomes. The main outcome measure was the FHSQ and VAS which are both reliable and valid outcome measures.<br><br>Comparable methods of outcome measurement involving the same measurement methods and thresholds were used at comparable time points.<br><br>As the outcomes were participant reported and the participants were blinded to their intervention, the outcome assessor (i.e. the participant) was blinded.

Selection of the reported result	Low risk	Data was analysed in accordance with a pre-specified analysis plan as highlighted with The trial was registered with the Australian Clinical Trials Registry (ACTRN012605000046606)<br><br>All eligible reported results of the pain and function domains corresponded to all intended outcome measurements.<br><br>All eligible reported results for the outcome measurements correspond to all the intended analyses

6. Limitations

6.1. Quality analysis

Recent editorials in BJSM (Büttner et al., 2020a and Büttner et al., 2020b), published after our study was carried out, have recommended that summary and cut-off scores – such as those we used to determine study inclusion - may mask high RoB in studies included in systematic reviews, hence an analysis of the studies informing the main determination of efficacy was performed in order to ensure confidence in practice recommendations (Table 1). Reassuringly, (Supplementary File 1) the included studies were found to typically demonstrate low risk of bias in the majority of domains. Future work may consider these recommendations further at the design stage, and consider alternative ways of excluding low quality studies at high risk of bias from high quality systematic reviews.

It could be argued, due to the components of the PEDro score, that this review was biased toward interventions where it is possible to blind the clinician delivering an intervention, and the participant receiving the intervention. However, non-pharmacological interventions (e.g. physical therapy or exercise), where it is not possible to blind the clinician or participant, can still meet other criteria that influence treatment effects such as allocation concealment and blinding of outcome assessors. Researchers are encouraged to apply available methodological strategies to ensure validity of the findings and confidence in reported effects.

References

Büttner F, Winters M, Delahunt E, et al. Identifying the 'incredible'! Part 1: assessing the risk of bias in outcomes included in systematic reviews. *Br J Sports Med* 2020a;54:798-800.

Büttner F, Winters M, Delahunt E, et al. Identifying the 'incredible'! Part 2: Spot the difference-a rigorous risk of bias assessment can alter the main findings of a systematic review. *Br J Sports Med* 2020b;54:801-8.

6.2. Data analysis

We used the SMD to standardise the results to a uniform scale for those outcomes that were measured in different ways as recommended by the Cochrane Handbook for Systematic Reviews of Interventions. We believe that consistency facilitates comparison between all studies.

For the reader, we have calculated the MDs (see below) for those studies that used the same outcome measure (which only applied to single studies)

Custom orthoses

Overall pain (MT): MD -1.60 [-2.91, -0.29]

Function (MT): MD -7.50 [-16.28, 1.28]

Overall pain (LT): MD -0.80 [-8.88, 7.28]

Function (LT): MD -2.40 [-10.27, 5.47]

Prefabricated foot orthoses

Overall pain (LT): MD -1.50 [-8.94, 5.94]

Function (LT): MD: -1.70 [-9.88, 6.48]

Magnetised insoles:

First step pain (ST): MD: 0.00 [-1.02, 1.02]

Radial ESWT

Overall pain (MT): MD -6.90 [-7.90, -5.90]

Function (ST): MD -17.70 [-30.37, -5.03]

Function (MT): MD -1.84 [-2.26, -1.42]

Function (LT): MD -0.92 [-1.47, -0.37]

Focused ESWT

Overall pain (ST): MD -14.20 [-24.07, -4.33]

First step pain (MT): MD -2.60 [-3.83, -1.37]

First step pain (LT): MD -2.90 [-4.03, -1.77]

Dry needling:

First step pain (ST): MD -9.00 [-18.15, 0.15]

Overall pain (ST): MD -6.50 [-14.93, 1.93]

Function (ST): MD 2.30 [-6.27, 10.87]

Calf Stretching:

First step pain (ST): MD: -11.40 [-23.37, 0.57]

Overall pain (ST): MD 0.10 [-10.04, 10.24]

Function (ST): MD -6.00 [-16.19, 4.19]

Foot taping:

First step pain (ST): MD -12.60 [-23.52, -1.68]

Overall pain (ST): 6.50 [-2.29, 15.29]

Function (ST): -1.10 [-10.70, 8.50]

PF Stretching:

First step pain (ST): MD 2.70 [1.85, 3.55]

First step pain (MT): MD 1.70 [0.66, 2.74]

First step pain (LT): MD -0.10 [-1.06, 0.86]

6.3.Sample size bias

It is possible that the pooled SMD and OR may suffer from small-sample bias (Lin, 2018). Although the sample sizes in studies that could be included in this systematic review are quite large in comparison to other MSK research, and we have set a minimum based on a stringently set MCID and SD.

Reference

Lin L. Bias caused by sampling error in meta-analysis with small sample sizes. PLoS One. 2018 Sep 13;13(9):e0204056. doi: 10.1371/journal.pone.0204056. PMID: 30212588; PMCID: PMC6136825

6.4.Sample size considerations

Of the 51 included trials, only 8 could be evaluated for primary proof of efficacy and one for secondary proof of efficacy due to the remaining trials being underpowered or not having a suitable control. We considered that a trial with a sample size less than 38 per group was inadequately powered and was therefore not considered for proof of efficacy in isolation. The pre-specified sample size of 38 was based on the minimal important difference for first step pain measured using a VAS, rather than overall pain, as first step pain is arguably the pathognomonic feature of PHP. The inclusion of trials with small sample sizes can inflate effect sizes and these stronger effects might not be representative of the true treatment effect,(Dechartres et al., 2013) which impacts on the validity of the findings and recommendations made to patients, clinicians and funders. It is recommended that researchers perform a priori sample size calculations to ensure the trial has adequate power to detect a difference between groups if a difference truly exists. Researchers are encouraged to calculate the required sample size using the minimal important difference that exists for the VAS and/or FHSQ in this population, (Landorf et al., 2010) rather than calculating a sample size based on effects from meta-analyses that have included underpowered trials.

References

Dechartres A, Trinquart L, Boutron I, Ravaud P. Influence of trial sample size on treatment effect estimates: meta-epidemiological study. *BMJ* 2013;346.

Landorf KB, Radford JA, Hudson S. Minimal important difference (MID) of two commonly used outcome measures for foot problems. *J Foot Ankle Res* 2010;14:3:7.

6.5.Additional limitations

First, reviewers who performed the PEDro analysis were not blinded to the study author, which might have introduced bias. Second, the panel of expert clinicians (podiatrists, podiatric surgeons, physiotherapists, sports physician and a rheumatologist) represents the views of the majority but not all disciplines involved in the assessment and management of PHP. Third, the Framework analysis was conducted by a physiotherapist (DM), which could have biased the findings, although this individual has extensive experience in qualitative research and has previously published two reviews (Barton et al., 2015 and Rowe et al., 2012). that combined best available evidence with expert reasoning. Additionally, the results in this study were extensively debated within the multidisciplinary author team. Finally, there was incomplete reporting in several trials, which limited the available data for meta-analysis.

References

Barton CJ, Lack S, Hemmings S, Tufail S, Morrissey D. The ‘Best practice guide to the conservative management of patellofemoral pain: incorporating level 1 evidence with expert clincial reasoning. *Br J Sports Med* 2015;49:923-34.

Rowe V, Hemmings S, Barton CJ, Malliaris P, Maffuli N, Morrissey D. Conservative management of midportion achilles tendinopathy. *Sports Med* 2012;42:941-67.

7. Implications for further research

There are several implications for further research. There is clearly a need for more robust, adequately powered trials with improved reporting of outcomes. At a minimum, researchers must include the mean and a measure of variability at all time points to minimise research waste. There is also a need for an established set of outcome measures that is specific to this population, which will reduce heterogeneity and improve potential for pooling of studies in a meta-analysis.   highlighted the importance of patient education as a core treatment for PHP, although no trials included a methodologically robust educational intervention developed in line with best practice such as the MRC framework.(Craig et al., 2008) Furthermore, many experts described the value of exercise, particularly strength based programs, although no trial suitable for inclusion in this review attempted to isolate the specific effect of strengthening exercises. Future research should focus on trials that compare one intervention to another of known efficacy. It is not appropriate to compare two or more active interventions that have no proof of efficacy as the superiority of one intervention, over another, might simply reflect a negative effect of an intervention in the comparator group(s). Following the publication of new high-quality efficacy research, the BPG should be revisited and amended.

Views of experts reinforced the need to conduct trials stratified by subgroups such as gender, physical activity level and BMI. However, sub-groups with particularly positive or negative prognosis were not identified in this study, which would be useful to guide research to target those most at risk of poor outcome. Additionally, trials may need to be stratified based on the underlying pathology, with pain experienced in this region of the foot potentially relating to a noxious stimulus in the plantar fascia, muscle, fat, nerve and/or bone. Until the underlying pathology of PHP is appropriately linked to a person's signs and symptoms, trials will continue to include participants with a likely heterogeneous group of pathologies subjected to poorly targeted interventions.

References

Craig P, Dieppe P, Macintyre S, Michie S, Nazareth I, Petticrew M. Developing and evaluating complex interventions: the new Medical Research Council guidance. *BMJ* 2008;337:a1655.
