# Supplementary material for: Management of plantar heel pain: a best practice guide informed by a systematic review, expert clinical reasoning and patient values
Source: Br J Sports Med. 2021 Mar 30;55(19):1106–18. doi: 10.1136/bjsports-2019-101970 (PMC8458083; doi:10.1136/bjsports-2019-101970)
Supplement: Supplementary data [file bjsports-2019-101970supp002.pdf]

Multiple choice questions

1. The ‘core’ approach to the treatment of plantar heel pain includes what combination of treatments:

a) Stretching, dry needling, ESWT
b) Stretching, foot taping and educational interventions
c) Stretching, education interventions and footwear advice
d) Stretching, foot taping and ESWT

2. The ‘core’ approach to the treatment of plantar heel pain is recommended for a period of 6 weeks before consideration of which combination of adjunctive interventions:

a) Dry needling and ESWT
b) ESWT and custom made orthoses
c) ESWT and a corticosteroid injections
d) Dry needling and strengthening exercises

3. According to the levels of evidence criteria adopted by Van Tulder, defined in this study, a moderate level of evidence describes:

a) Consistent findings from multiple high-quality RCTs
b) Findings from a single high-quality RCT
c) Findings from a single high-quality study with an inadequate sample size
d) Consistent findings from multiple low quality studies

4. Which of the following statements is true regarding the effectiveness of treatments for plantar heel pain:

a) There is moderate evidence that plantar fascia stretching is superior to radial ESWT for first step pain in the short term
b) There is strong evidence for the efficacy of custom foot orthoses versus sham for pain in the long term
c) There is limited evidence for the efficacy of focused ESWT for overall pain in the long term
d) There is strong evidence for the efficacy of calf stretching in the medium term

5. As part of the ‘core’ approach to plantar heel pain, individualised decisions about education content should consider:

a) Load management, pain education, stretching and taping
b) Load management, pain education, ESWT and custom orthoses
c) Load management, pain education, footwear and long-term conditions
d) Load management, pain education, taping and dry needling
